# SMC1B is present in mammalian somatic cells and interacts with mitotic cohesin proteins

**DOI:** 10.1038/srep18472

**Published:** 2015-12-17

**Authors:** Linda Mannini, Francesco Cucco, Valentina Quarantotti, Clelia Amato, Mara Tinti, Luigi Tana, Annalisa Frattini, Domenico Delia, Ian D. Krantz, Rolf Jessberger, Antonio Musio

**Affiliations:** 1 Istituto di Ricerca Genetica e Biomedica, Consiglio Nazionale delle Ricerche, Pisa, Italy; 2 Azienda Ospedaliero Universitaria Pisana, U.O. Fisica Sanitaria, Pisa, Italy; 3 Istituto di Ricerca Genetica e Biomedica, Consiglio Nazionale delle Ricerche, Milan, Italy; 4 Dipartimento di Medicina Clinica e Sperimentale, Università degli Studi dell’Insubria, Varese, Italy; 5 Fondazione IRCCS Istituto Nazionale Tumori, Department of Experimental Oncology, Milan, Italy; 6 Division of Human Genetics, The Children’s Hospital of Philadelphia and the Perelman School of Medicine at the University of Pennsylvania, Philadelphia, USA; 7 Institute of Physiological Chemistry, Technische Universität Dresden, Dresden, Germany

## Abstract

Cohesin is an evolutionarily conserved protein complex that plays a role in many biological processes: it ensures faithful chromosome segregation, regulates gene expression and preserves genome stability. In mammalian cells, the mitotic cohesin complex consists of two structural maintenance of chromosome proteins, SMC1A and SMC3, the kleisin protein RAD21 and a fourth subunit either STAG1 or STAG2. Meiotic paralogs in mammals were reported for SMC1A, RAD21 and STAG1/STAG2 and are called SMC1B, REC8 and STAG3 respectively. It is believed that SMC1B is only a meiotic-specific cohesin member, required for sister chromatid pairing and for preventing telomere shortening. Here we show that SMC1B is also expressed in somatic mammalian cells and is a member of a mitotic cohesin complex. In addition, *SMC1B* safeguards genome stability following irradiation whereas its ablation has no effect on chromosome segregation. Finally, unexpectedly *SMC1B* depletion impairs gene transcription, particularly at genes mapping to clusters such as *HOX* and *PCDHB*. Genome-wide analyses show that cluster genes changing in expression are enriched for cohesin-SMC1B binding.

Mitotic sister chromatid cohesion is ensured by cohesin, an evolutionarily conserved complex composed of four members, SMC1A, SMC3, RAD21 and STAG1/2. In addition to its canonical role, cohesin also regulates gene expression, and acts in DNA repair and maintenance of genome stability^1^,^2^,^3^. Due to the involvement of cohesin in these various biological processes that are critical for proliferation and differentiation, it is not surprising that mutations in cohesin and cohesin loading and regulatory factors are associated with cohesinopathies and cancer. In fact, mutations in *NIPBL, SMC1A, SMC3, RAD21, HDAC8* lead to Cornelia de Lange syndrome (CdLS)^4^,^5^,^6^,^7^,^8^,^9^,^10^. CdLS is a developmental disorder characterized by cognitive impairment, facial dysmorphism, pre- and post-natal growth delay and upper extremity anomalies^11^. CdLS cell lines show no defects in sister chromatid cohesion^12^,^13^ suggesting that impairments in cohesion^12^,^13^ are not likely to be the molecular basis for CdLS. Experimental evidence supports the notion that cohesin takes part in gene transcription, through long-range interactions with regulatory elements associated with promoters and enhancers^14^,^15^,^16^ or with CTCF^17^,^18^,^19^,^20^. Furthermore, a modest reduction in cohesin levels leads to gene transcription without affecting sister chromatid cohesion^21^. Somatic mutations in genes that regulate sister chromatid cohesion have been recently identified in human cancers including colon cancer, urothelial bladder cancer and acute myeloid leukemia^22^,^23^,^24^,^25^,^26^. Besides mutations, cohesin pathway genes have been found down- or upregulated in many human cancers^3^.

Meiotic chromosome segregation is more complex than mitotic chromosome segregation, since chromosomes pass through two cycles of segregation at meiosis I and II. Importantly, mammalian meiocytes express two distinct versions of cohesin complexes, which feature either SMC1A or SMC1B. It is currently thought that SMC1B is a meiosis-specific cohesin member. *Smc1b*^*−/−*^ mice are sterile due meiotic failure^27^ and it has been shown that SMC1B determines meiotic chromatin loop/axes organization, contributes to sister chromatid cohesion and chromosome synapsis, and protects telomeres from rearrangement^28^,^29^,^30^. There is no obvious somatic phenotype of *Smc1b*^*−/−*^ mice.

Here, we present evidence that SMC1B is also expressed in both somatic mouse tissues and human primary fibroblasts and associates with SMC3 and RAD21, making it a member of the mitotic cohesin complex. In addition, we find that SMC1B participates in cellular response to DNA damage, whereas *SMC1B* silencing does not affect both chromosome number and morphology. Notably, we revealed that *SMC1B* depletion leads to gene expression dysregulation; in particular, significant changes occurred in genes mapping to clusters. Finally, ChIP-seq data show that dysregulated cluster genes are enriched for cohesin-SMC1B.

Starting out by addressing the question of whether SMC1B is expressed in mammalian cells, we showed that SMC1B participates in maintaining genome stability, and revealed a new role for cohesin-SMC1B in gene expression.

## Results

### SMC1B is expressed in somatic mouse tissues and interacts with mitotic core cohesin proteins

**Figure 1:**
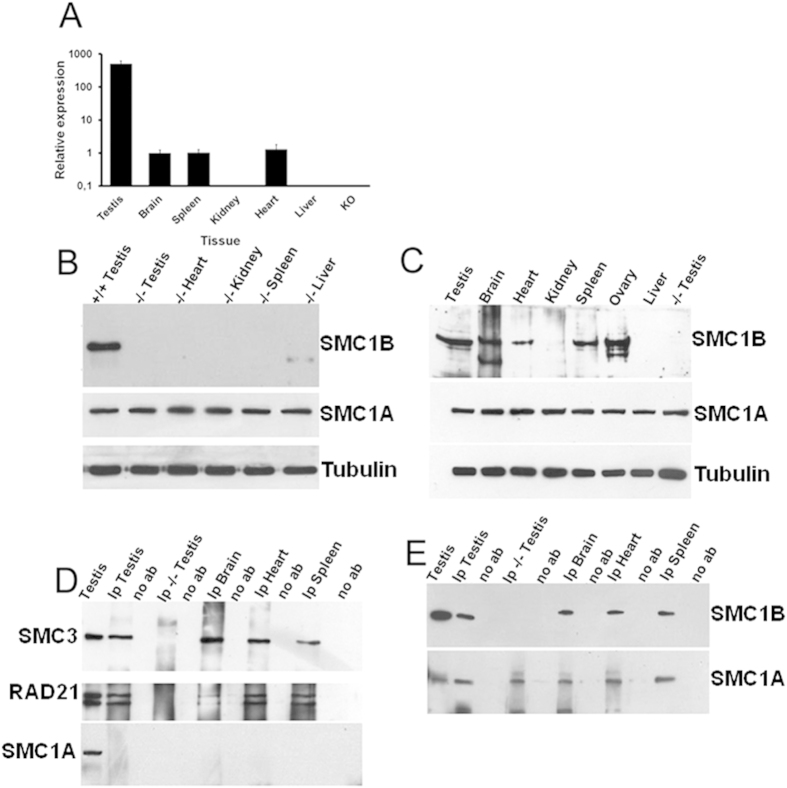
*Smc1b* is expressed in both germ and somatic mouse cells. (**A**) In addition to testes, *Smc1b* is also transcribed in brain, spleen and heart mouse tissues when analyzed by RT-qPCR. No expression was detected in kidney, liver and *Smc1b*−/− testis (as negative control). (**B**) The specificity of 308–432 polyclonal antibody was assayed using protein extracted from different *Smc1b* knock-out mouse tissues. No signal was detected in testis, heart, kidney, spleen or liver. A clear signal was visualized in wild-type mouse tissue. (**C**) *Smc1b* RNA is also translated and bands are visualized in brain, heart, and spleen, in addition to reproductive tissues (testis and ovary). No band observed in kidney, liver or *Smc1b*−/− testis. An antibody against tubulin was used as loading control. (**D**) SMC1B co-IPed with SMC3 and RAD21 in testis, brain, heart and spleen whereas it did not co-IP in *Smc1b*−/− testis or without no antibody (no Ab). Total protein extract from wild-type testis tissue was used as positive control. SMC1A does not co-IP with SMC1B. (**E**) SMC3 was found to be co-precipitated with both SMC1A and SMC1B. Western blot images regarding flow-through are in Supplementary Fig. S3.

We asked whether SMC1B is indeed meiosis-specific or may be also expressed in primary mammalian somatic cells. A variety of mouse tissues were used to extract both RNA and proteins. As expected, we found that *Smc1b* is highly expressed in the testes when analyzed by quantitative reverse-transcription PCR (RT-qPCR), but surprisingly it is also expressed in brain, heart and spleen (Fig. 1A). Previously published works did not report the expression of *Smc1b* in primary somatic cells^31^, though its expression has been shown in embryonic stem cells^32^. The mechanism underlying the differential expression of *Smc1b* in somatic cells is uncertain. It is possible to speculate that *Smc1b* promoter is less accessible to the transcription machinery in brain, heart, and spleen than in kidney and liver or that its expression depends on several factors including mouse strain, gender and/or age. Because of the high degree of similarity between *Smc1a* and *Smc1b*, we sequenced the qPCR products. Results showed that RT-qPCR specifically amplified only the *Smc1b* gene (Supplementary Fig. S1A–C). This result prompted us to investigate whether the *Smc1b* transcripts are also translated. In addition to the commercially available and published antibodies (see Material and Methods), we produced an antibody against the amino acid fragment 308–432 of the SMC1B protein, a conserved region in both human and mouse proteins (Supplementary Table S1). The specificity of the produced antibody was assayed by Western blot using *Smc1b* knock-out samples. No signal was detected in testes, heart, kidney, spleen or liver (Fig. 1B). Western blotting experiments showed that SMC1B is expressed in brain, heart, spleen, as well as in positive control tissue samples, testes and ovaries. The signal intensities were quite distinct, since heart extracts showed only a faint band, while brain and spleen showed a more intense signal and testis and ovary a very strong signal. No signal was detected in *Smc1b* knock-out, liver and kidney samples (Fig. 1C). This data was also corroborated by using a previously published antibody^31^ (Supplementary Fig. S2A), further confirming the specificity of our antibody. In addition, we showed that SMC1B is present only in nuclear extracts from a variety of mouse tissues (Supplementary Fig. S2B,C). This is the first observation that SMC1B protein is also expressed in somatic cells. A prior study using GFP expressed under the control of a 256 bp *Smc1b* promoter region was not expressed in liver and kidney when tissue cells from wild-type mice were analyzed by cytofluorimeter; however, a small peak was seen in spleen cells and was considered to be autofluorescence^29^. In light of our result, the spleen cytofluorimeter signal may in fact be due to the specific activity of the *Smc1b* promoter fragment. In addition, bands corresponding to *Smc1b* transcripts were detected in a number of primary mouse tissues analyzed by RT-qPCR^33^. Stronger and very similar signals were detected in all samples when the same Western blotting membrane used for SMC1B signal was incubated with an antibody against SMC1A (Fig. 1C). To assess whether SMC1B is a component of mitotic cohesin, we performed co-immunoprecipitation (co-IP) of SMC1B with other cohesin components, identified by Western blotting and probing for cohesin members. The co-IP experiments showed the physical association of SMC1B with both SMC3 and RAD21 in brain, heart and spleen extracts. No SMC3 or RAD21 signal was detected in control Western blotting using IgG-coated beads (Fig. 1D). We never observed SMC1A co-precipitating with SMC1B (Fig. 1D). In addition, both SMC1A and SMC1B co-precipitated with anti SMC3 (Fig. 1E). Interestingly, when samples from the flow-through were electrophoresed and visualized by Western blot using anti-SMC1B antibody, no signal corresponding to SMC1B was detected (Supplementary Fig. S3), suggesting that all SMC1B is engaged in the cohesin complex.

Altogether, these results suggest that SMC1B protein is expressed in both germ cells and somatic mammalian cells and associates with other cohesin proteins. This is in agreement with the suggestion of an SMC1B-RAD21 complex in meiocytes^34^.

### SMC1B is involved in DNA damage response but its depletion has no effect on cohesion

**Figure 2:**
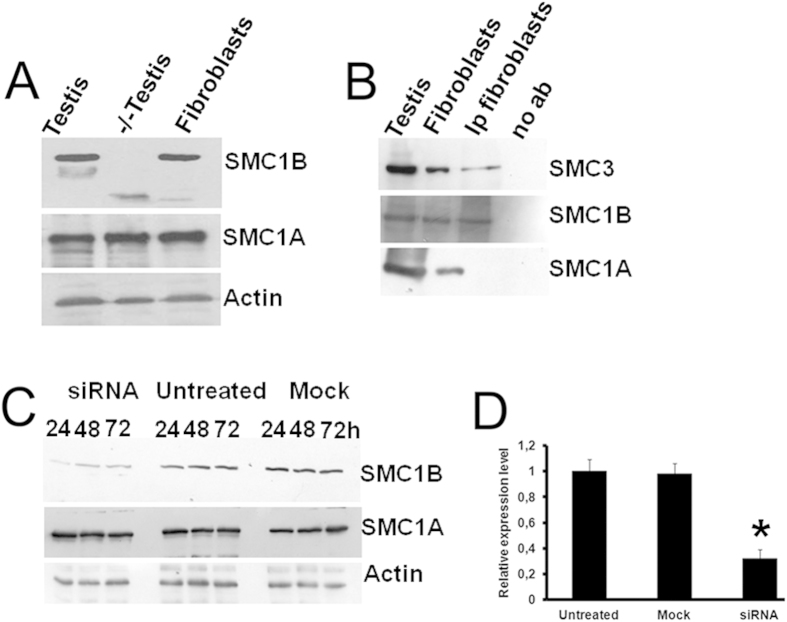
*SMC1B* is expressed in human fibroblasts. (**A**) Total protein extracts from wild-type testis tissue and *Smc1b* knockout testes were respectively used as positive and negative controls. (**B**) SMC1B was found to interact physically with SMC3 cohesin factor by IP also in human fibroblast whereas no signal was detected for SMC1A. Total testis and fibroblast extracts were used as control. The rabbit polyclonal antibody against the fragment 308–432 was used. (**C**) Western blotting showing the downregulation of *SMC1B* in human fibroblasts treated with 20 nM of smart pool siRNA. siRNA treatment had no effect on *SMC1A* expression. (**D**) *SMC1B* silencing was also confirmed by RT-qPCR following 24 hr siRNA treatment. *p < 0.05.

We next tested whether SMC1B is also expressed in human primary fibroblasts. A band corresponding to SMC1B was identified by Western blotting in human primary fibroblasts. As expected, signals corresponding to SMC1A were also evident (Fig. 2A). We confirmed that SMC1B interacts with the SMC3 core cohesin subunit in human fibroblasts by co-IP (Fig. 2B).

In order to investigate the possible functions of *SMC1B*, we depleted *SMC1B* using short interfering RNA (siRNA) in human primary fibroblasts. Western blots showed that specific inhibition of SMC1B synthesis was obtained 24 h after transfection. No decrease in SMC1B synthesis was observed in both untreated control and mock cells (Fig. 2C). Re-probing the same plot with SMC1A antibody showed that siRNA treatment had no effect on SMC1A synthesis, whereas an anti-actin antibody confirmed equal loading (Fig. 2C). The downregulation of *SMC1B* was also confirmed by RT-qPCR and the reduction in the transcription level was statistically significant (P < 0.05) when compared with both untreated and mock cells (Fig. 2D). We then investigated the progression of the cell cycle following *SMC1B* depletion. Silenced cells were stained with propidium iodide and analyzed by flow cytometry. Results showed that cell cycle progression following *SMC1B* depletion was similar to the control cell line (Supplementary Fig. S4).

**Figure 3:**
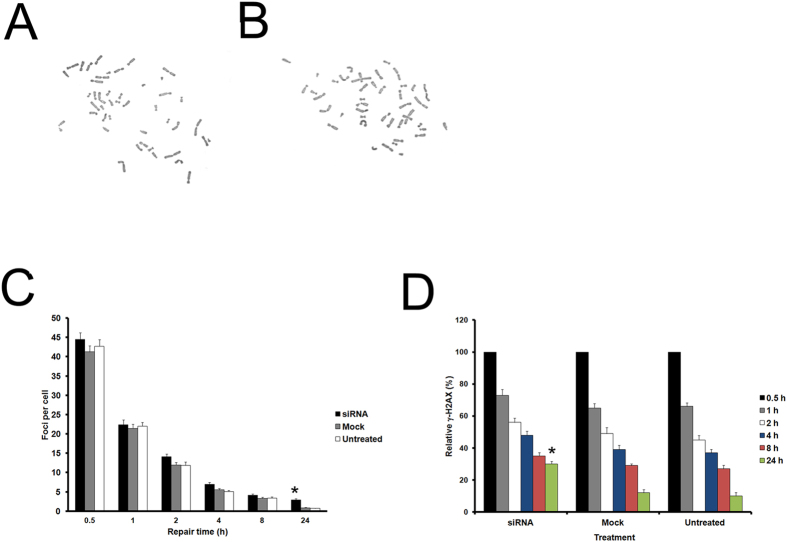
Effects of *SMC1B* depletion in human primary fibroblasts. (**A**) siRNA treatment had no effect on either chromosome number or morphology. (**B**) Diploid control metaphase. (**C**) γ-H2AX analysis in untreated control, mock and siRNA-treated cells irradiated with 5 Gy. The mean number of foci/cells for different repair times is shown. Error bars represent the SE from the analysis of 300 cells from three independent experiments. (**D**) Relative γ-H2AX phosphorylation levels determined after irradiation with 5 Gy by flow cytometry in control, mock and siRNA-treated cells for different repair times. Values for the fluorescence signal intensity were normalized to the value of the corresponding 0.5 h sample arbitrarily set as 100%. Both the number of foci/cell and fluorescence intensity was found to be statistically significant different 24 h after irradiation. *p < 0.05.

The classic role of cohesin is to ensure correct chromosome segregation, so we then investigated whether or not *SMC1B* depletion would result in aneuploidy. One hundred Giemsa-stained metaphase spreads from *SMC1B* downregulated and mock cells were analyzed. As shown in Table 1, the frequency of aneuploid cells was identical (3%) in both *SMC1B-*depleted and mock cells. Furthermore, siRNA-treated cells showed chromosomes with normal morphology and normally paired centromeres (Fig. 3A) when compared to control chromosomes (Fig. 3B). In addition, we investigated other markers of chromosomal instability to further substantiate this data. Micronuclei formations can arise as a consequence of defects in the segregation process. We did not observe an increase in the number of micronuclei present in *SMC1B* silenced cells compared with control cells (data not shown). Many studies indicate that cohesin plays a role in maintenance of genome stability and DNA repair^35^,^36^,^37^,^38^. Therefore, we tested whether *SMC1B* silencing affects genome stability. We found that the frequency of spontaneous chromosome aberrations was similar in siRNA-treated and control cells, 0.03 ± 0.17 and 0.04 ± 0.19 respectively (Table 1). To investigate whether *SMC1B* depletion increases DNA damage, γ-H2AX focus formation was examined at several time points after irradiation with 5 Gy. Using a fluorescent antibody specific for γ-H2AX, foci were very rare in siRNA-treated and in both control and mock cells (data not shown). Thirty minutes after irradiation, the number of foci observed in *SMC1B*-depleted cells was similar to those in control and mock cells. The mean values were 44.48, 41.24 and 42.7 foci per cell for *SMC1B*-silenced, mock and untreated cells respectively (Fig. 3C). This difference was not statistically significant (data not shown). After 1 h, about 50% of DSBs were repaired in both control and irradiated cells. For repair times of 2 h and longer, siRNA-treated cells showed more foci than control cells for all time points and this difference was statistically significant (P < 0.05) 24 h after irradiation (Fig. 3C). Notably, the kinetics of foci disappearance after 5 Gy closely resembles the kinetics of relative γ-H2AX phosphorylation levels (Fig. 3D). It has been shown that ATM phosphorylates SMC1A on serine 957 and 966 following ionizing irradiation^35^. Serine or threonine followed by a glutamine residue is the requisite target sequence for phosphorylation by ATM^39^. SMC1B contains seven SQ residues and eight TQ residues. To investigate whether ATM directly phosphorylates SMC1B after ionizing irradiation, we treated cells with 10 Gy and performed co-IP experiments 1 h after exposure. We never observed ATM co-precipitating with SMC1B, whereas we showed the physical association of ATM with SMC1A (Supplementary Fig. S5).

Altogether, these results suggest that cohesin-SMC1B it is not essential for maintaining centromere cohesion whereas it plays a part in genome stability maintenance by allowing DNA repair in response to ionizing irradiation.

### *SMC1B* silencing impairs gene transcription

**Figure 4:**
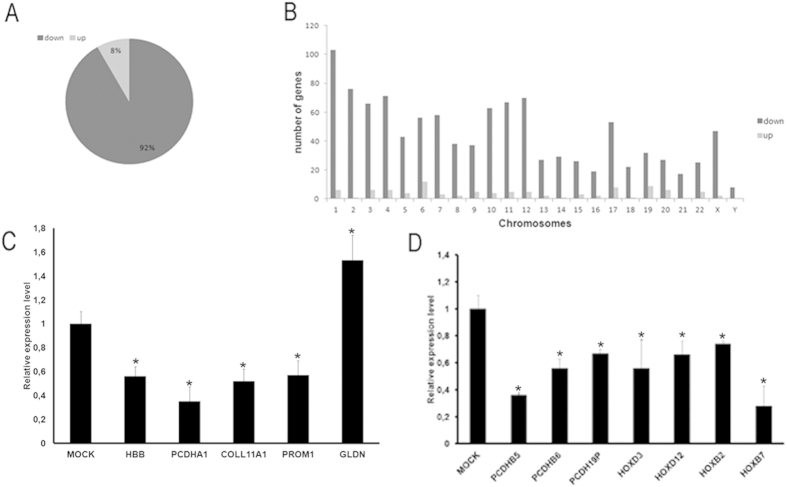
*SMC1B* depletion causes gene dysregulation. (**A**) In human primary fibroblasts the inhibition of *SMC1B* led to gene expression dysregulation, 92% of genes were downregulated (black) and 8% were upregulated (grey). (**B**) Chromosome 1 showed the highest number of downregulated genes (103) while Y chromosome the lower (8). No upregulated gene was found on chromosomes 21 and Y. (**C**) Microarray data was validated for five genes, *COL11A1, GLDN, HBB, PCDHA1* and *PROM1,* by RT-qPCR. (**D**) The dysregulation of *HOXD3, HOXD12, HOXB2, HOXB7, PCDHB5, PCDHB6, PCDHB19* and *PCDHB* was validated by RT-qPCR. *p < 0.05.

**Figure 5:**
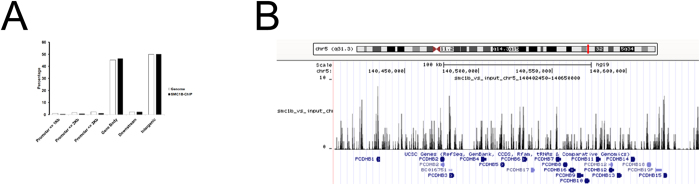
Genome-wide distribution of cohesin-SMC1B binding sites. (**A**) SMC1B binding in normal human fibroblast cells represented as percentage of sites detected at promoter, downstream, gene body and intergenic regions. Cohesin-SMC1B peaks were aligned to RefSeq gene annotations by the use of CEAS tool. We compared binding of SMC1B to a selected region to the average genome-wide binding. (**B)** Genomic binding of SMC1B at *PCDHB* cluster on chromosome 5 as determined by ChIP-sequencing.

Beyond its role in sister chromatid cohesion, emerging data show that cohesin plays a pivotal role in regulating gene expression^15^,^16^,^40^,^41^,^42^. To gain insight into the possible role of *SMC1B* in transcriptional regulation, we performed expression profiling of *SMC1B* downregulated cells. Transcriptome analysis was performed using Agilent microarrays. The 1,209 probe sets, corresponding to 1,178 genes, displayed statistically significant differences after *SMC1B* depletion, with a False Discovery Rate (FDR) <0.001. In particular, of the 1,178 mis-regulated genes 1,080 (92%) were downregulated. Of the aberrantly regulated genes 98 (8%) were upregulated (Table 2, Fig. 4A,B). Validation of microarray data was performed by RT-qPCR for a subset of five of the most statistically significantly dysregulated genes: *COL11A1, GLDN, HBB, PCDHA1* and *PROM1* (Fig. 4C). The transcriptional effects were small with fold changes ranging from 1.06 to 4.21 and from −1.03 to −5.62 for up- and downregulated genes respectively. Dysregulated genes encoding proteins belonging to virtually all functional categories were found (Supplementary Table S2), with no specific enrichment of any particular functional sets (by Gene Spring GX 11.0, data not shown). Though automated analysis failed to identify any specific functional class, manual examination revealed that significant changes occurred in a few genes mapping to clusters including; *HOXD3* and *HOXD12* from the 9-gene cluster on chromosome 2, *HOXB2* and *HOXB7* from the 11-gene cluster on chromosome 17 and *PCDHB5, PCDHB6* and *PCDHB19*, from the 19-gene *PCDHB* cluster on chromosome 5, all of which were downregulated. All of these mis-regulations were validated by RT-qPCR (Fig. 4D). Next, we performed chromatin immunoprecipitation followed by massive parallel DNA sequencing (ChIP-seq) in primary fibroblasts using an SMC1B antibody. Genome-wide analysis of the sequenced tags using MACS peak algorithm with a cutoff P-value of ≤0.0001 and FDR < 0.05 revealed 5943 SMC1B binding sites. ChIP-seq data was validated for a subset of these regions by RT-qPCR (Supplementary Fig. S6). Cohesin-SMC1B association with transcriptional elements was determined using RefSeq gene annotations by a cis-regulatory element annotation system (CEAS)^43^. Genome-wide analysis indicated that genomic regions containing the highest density of cohesin-SMC1B are mainly at gene body and intergenic regions (Fig. 5A). In addition, ChIP-seq data showed that SMC1B maps at the *PCDHB* (Fig. 5B) and *HOX* clusters (Supplementary Fig. S7). These observations suggest a possible role of *SMC1B* in long-range chromosomal regulatory interactions – a key function of mitotic cohesion^44^.

### Mutational screening of *SMC1B* in CdLS

Mutations in cohesin genes are responsible for CdLS, and recent data supports the disruption of cohesin’s role in the regulation of gene expression as the main pathogenetic mechanism resulting in CdLS and a growing group of disorders known as the cohesinopathies^11^,^45^. The finding that *SMC1B* appears to be involved in mitotic cohesin function and that its downregulation affects gene expression led us to hypothesize that it could be causative for CdLS when mutated. To test this we sequenced all exons of *SMC1B* and their adjacent intronic sequences (harboring critical splice site elements) in 120 unrelated CdLS probands without an identifiable mutation in one of the five known disease genes (primers are listed in Supplementary Table S3). *SMC1B* sequencing identified nineteen polymorphisms (5 amino acid changes with no effect on protein activity, according to the Sorting Intolerant From Tolerant program (SIFT, http://sift-dna.org) (data not shown), 9 neutral changes and 5 intronic variants) (Supplementary Table S4). We also identified one proband of Caucasian origin with a heterozygous c.2078G > A missense mutation in exon 13 leading to a p.R693H amino acid change mapping in the coiled-coil domain of SMC1B protein. The effect of the p.R693H mutation is probably damaging, according to the SIFT program (data not shown). In addition, analysis of protein sequences for humans and a number of animal models aligned by the ClustalW method (http://www.ebi.ac.uk/Tools/msa/clustalw2) showed that the mutated residue affects evolutionarily conserved amino acids (data not shown). This change was also present in the unaffected mother. The nucleotide change is absent from >400 normal chromosomes, and is not described in the 1000 genomes project database while it is reported with a very low frequency (3:10,000) in Exome Sequence Project (ESP5400), which includes only subjects with pathological conditions, including cancer. The identification of the same *SMC1B* mutation in both CdLS patient and the unaffected mother argues against its involvement in CdLS. However, *SMC1B* mutational screening in a larger CdLS patients cohort will elucidate this issue.

## Discussion

Cohesin regulates many processes that occur in chromosomes, such as segregation, DNA repair, condensation, nuclear architecture, and gene expression^1^,^2^,^46^. The mitotic core cohesin complex is composed of four subunits: SMC1, SMC3, RAD21 and STAG1/2. During meiotic cell divisions, meiosis-specific isoforms of several of the cohesin subunits are incorporated into distinct meiotic cohesin complexes. In particular, there are two SMC1 variants (A and B) and SMC1B is thought to be specific to meiocytes^47^,^48^. Our results, besides confirming that *SMC1B* is highly expressed in germ cells, showed the first clear evidence that it is also expressed in somatic mammalian cells, though to a lesser degree than in germ cells, as was marginally suggested in a previous study^33^. In addition, expression of *SMC1B* as assayed by microarray and RNA-seq analyses has been reported in many human tissues in the Genecards database (www.genecards.org), further demonstrating that its expression is not limited to meiocytes.

By using γ-H2AX foci analysis as an approach for DSB repair measurement, we showed that *SMC1B* ablation leads to DNA repair defects in human primary fibroblasts. In fact, though *SMC1B*-silenced cells repair most DSBs with a trend similar to that of control cells, a small fraction of irradiation-induced breaks remains unrepaired. These findings agree with the γ-H2AX fluorescence intensity. It is plausible to assume that DNA repair may depend on the complexity of the damage at individual sites and the lack of *SMC1B* interferes with repair processes so that residual γ-H2AX foci mark permanent DNA damage. It is well-known that SMC1A is phosphorylated by ATM after irradiation^35^. We found no physical association between SMC1B and ATM. These initial insights raise new questions about molecular mechanisms involving DNA damage response mediated by SMC1B.

Beyond genome stability maintenance, we present evidence that cohesin-SMC1B also takes part in gene expression. We found that 1178 genes were dysregulated after siRNA treatment and most of them (92%) were downregulated. In addition, we identified the genomic sites occupied by SMC1B by genome-wide analysis. We found that cohesin-SMC1B has a greater propensity to localize at intergenic and gene body regions. In addition, some of dysregulated genes belonged to gene clusters such as HOX and PCDHB, suggesting that SMC1B could be involved in the transcription regulation of clusters. This notion is further supported by the finding that these cluster genes were enriched for SMC1B. It is worth noting that gene expression profiling data showed that seven genes, namely *Pcdhb7, 16, 17, 19, 20, 21* and *22*, belonging to the *Pcdhb* cluster were dysregulated in the *Nipbl*^+/−^ mouse cells^49^. A similar result has been recently obtained in *Sa1* knock-out mice. In fact, *Sa1*-null cells showed downregulation of many members of the *Pcdha, b*, and *g* clusters^50^. These findings are consistent with the specific mitotic cohesin complexes, including cohesin-SMC1B, coordinate transcriptional regulation of gene clusters.

The relevance of the observation that *SMC1B* depletion impaired gene expression *in vitro* is tempered by the finding that the *Smc1b* knockout mouse is viable and does not manifest a phenotype (with the exception of sterility)^27^. We can speculate that in somatic cells *in vivo*, where the levels of SMC1B are very low, the putative function of SMC1B could be compensated by SMC1A. In contrast, in meiocytes where the levels of SMC1B are very high, the depletion of SMC1B results in infertility.

Mutations affecting several cohesion factors have been identified in CdLS patients, indicating that abnormal cohesin function is responsible for the anomalies associated with this syndrome^11^. The screening of *SMC1B* in our cohort composed by patients with CdLS or CdLS-like phenotype allowed us the identification of the c.2078G > A missense mutation leading to a p.R693H amino acid change. However, the same mutation has been also identified in the unaffected mother. It is unclear whether this is a rare benign variant or potentially pathogenic with either reduced penetrance or representing a recessive allele with a second mutation that was not detected by our sequence analysis. These findings suggest that *SMC1B* is not a major cause of CdLS; however, its contribution to causing human disease when mutated (e.g., resulting in a cohesinopathy with different features than those seen in CdLS) still needs to be further evaluated.

Our finding that *SMC1B* is expressed in somatic cells and is involved in both genome stability and gene expression is likely relevant for oncologic processes. In fact, mutations in *SMC1B* have been recently identified in urothelial bladder cancer^51^ and the Catalogue Of Somatic Mutation In Cancer database (COSMIC, http://cancer.sanger.ac.uk/cancergenome/projects/cosmic) contains 163 unique mutations in *SMC1B*. In addition, polymorphisms in the *SMC1B*-binding site for miRNA are associated with early-stage head and neck cancer^52^ and oropharyngeal cancer^53^. Since we found that *SMC1B* silencing led to gene expression dysregulation, it is plausible cohesin-SMC1B deregulation by mutation or epimutation may provide a potential source of destabilizing changes for allowing the transformation of a normal somatic cell into a cancer cell.

In summary, in addition to providing the first evidence that SMC1B protein is present in primary somatic tissues and that SMC1B can associate with mitotic cohesin proteins, our studies suggest a novel role for mitotic SMC1B in transcriptional regulation that is distinct from its well-characterized roles in maintaining meiotic sister chromatid cohesion.

## Material and Methods

### Cell culture

Normal human primary fibroblasts, established by skin biopsy from a 4-year-old Caucasian child undergoing surgical procedure^54^, were grown in Dulbecco’s minimal essential medium (DMEM, Gibco BRL) supplemented with 10% fetal calf serum and antibiotics in a humidified 5% CO_2_ atmosphere.

### Mouse tissues

B6C3 wild-type 3-month-old mice and *Smc1b* knock-out mice were used to obtain a variety of tissues: testis, ovary, brain, heart, spleen, kidney and liver.

### siRNA treatment

Smart pool siRNA against *SMC1B* was purchased from Dharmacon. Human primary fibroblasts (at 40–60% confluence) were transfected with 20 nM *SMC1B* siRNA by using Oligofectamine Reagent (Invitrogen) at the 16^th^ passage. Cells were analyzed for aneuploidy and genome stability 48 h post-transfection.

### Cytogenetic analysis

Colcemid was added to the cultures for 90 min, followed by a 20-min incubation in 0.075 M KCl at 37 °C and multiple changes of Carnoy’s fixative. Cells were dropped onto cleaned and wet slides. One hundred metaphases were analyzed. Chromosome aneuploidy and aberrations were visualized by staining slides in Giemsa stain and detected by direct microscope visualization.

### Irradiation and γ-H2AX foci

For investigating DNA repair following irradiation, asynchronous fibroblasts were exposed to 5 Gy (10 Gy for studying the hypothetic interaction between ATM and SMC1B) by a linear accelerator (Siemens Primus) with a 6 MV photon energy source. Cells were fixed in 2% paraformaldehyde for 10 min, permeabilized for 5 min on ice in 0.2% Triton X-100 and blocked in PBS with 1% BSA for 30 min at room temperature. Thereafter, cells were incubated with anti-γ-H2AX antibody (Trevigen) for 1 h, washed in PBS, 1% BSA and incubated with Alexa Fluor 488-conjugated goat anti-rabbit secondary antibody (Molecular Probes) for 1 h.

γ-H2AX foci were scored manually throughout the cell nuclei and the average number of foci per cell was calculated from at least 300 cells per dose/time point. Experimental data represent the average of three independent experiments.

### Antibodies

Published, and commercially available antibodies used in this study are as follows: rabbit polyclonal anti-ATM (Abcam), rabbit polyclonal anti-SMC1A (Bethyl), rabbit polyclonal anti-SMC3 (Bethyl), rabbit polyclonal anti-RAD21 (Bethyl), rabbit polyclonal anti-Actin (Bethyl) and mouse monoclonal anti-Tubulin (Sigma-Aldrich). In addition, we generated a rabbit polyclonal antibody against the fragment 308–432 (Supplementary Table S1) amino acids of SMC1B protein. The fragment 308–432 was subcloned into *Escherichia coli* containing the plasmid p2N. The His-tagged protein was over-expressed and purified by Immobilized Metal Chelating Chromatography (IMAC) in denaturing buffer, a robust method for purifying histidine-tagged recombinant proteins. Rabbits were boosted subsequently 4 times with protein (200 μg/rabbit) mixed with IFA at 2-weeks interval. After 3 boostings, polyclonal antibody serum was tested by enzyme-linked immunosorbent assay (ELISA).

### Immunoprecipitation

To perform immunoprecipitation experiments, a volume containing 800 μg of total protein extracts from mouse tissues or human fibroblasts was dissolved in 1 mL of incubation buffer. The solution were precleared with 20 μL Dynabeads protein A (Invitrogen) for 1 h. The supernatants were then incubated overnight at 4 °C with 5 μg of ATM, SMC1B or SMC3 antibody coupled to the 40 μL Dynabeads protein A. Samples were boiled in sample buffer and separated by SDS–PAGE.

### Whole protein extracts and subcellular fractionation

Whole protein extracts from mouse tissues and human fibroblast cells were resuspended with lysis buffer (Tris HCl pH 8,0, 25 μM, NaCl 55 μM, EDTA 1 μM, Protease Inhibitor Cocktail, Sigma- Aldrich) and protein concentration was estimated by the Bradford Protein Assay (Thermo Scientific). Proteins, 20–60 μg per lane, were separated by SDS-PAGE.

Subcellular fractionation was performed as previously described, with minor modification^55^. Briefly, samples were resuspended in 500 μl of buffer A (250 mM sucrose, 50 mM Tris–HCl, pH 7.4, 5 mM MgCl2, Protease Inhibitor Cocktail, Sigma-Aldrich) and homogenized for 2 min by 30 Hz using Tissuelyser (Qiagen). The homogenates were centrifuged at 800 *g* for 15 min at 4 °C. The pellets were processed for nuclear extraction whereas the supernatants were used for subsequent isolation of cytosolic fraction. The pellet was resuspended in 200 μl of buffer B (20 mM HEPES pH 7.9, 1.5 mM MgCl2, 0.5 M NaCl, 0.2 mM EDTA, 20% glycerol, 1% Triton-X-100, Protease Inhibitor Cocktail, Sigma-Aldrich) and incubated on ice for 30 min. The nuclei were lysated with 10–20 passages through an 18-gauge needle and finally the lysate was centrifuged at 9,000 g for 30 min and the nuclear fraction resulted in the supernatant. Both nuclear and cytosolic proteins, 20 μg per lane, were separated by SDS-PAGE.

### Western blotting

The proteins were transferred to nitrocellulose membranes (Amersham) and incubated with the primary antibody, ATM, RAD21, SMC1A, SMC1B or SMC3. After removal of the unbound primary antibody, membranes were incubated with secondary antibody-peroxidase conjugate (Sigma), processed for detection by chemiluminescence (Amersham) and imaged on Biomax film (Kodak). Actin and Tubulin antibodies were used as internal control.

### Flow cytometry

Samples in suspension were fixed in 70% ethanol at 4 °C, then treated with 1μg/ml RNaseA (Sigma) at 37 °C for 20 min, stained with 5μg/ml propidium iodide (Sigma) and analyzed for DNA content (25,000 cells/sample) with a FACScalibur flow cytometer (Becton Dickinson).

### Quantitative reverse-transcription PCR analysis (RT-qPCR)

RT-qPCR was carried out to validate the expression of *SMC1B* in both mouse tissues and human fibroblasts (primers listed in Supplementary Table S5) and in order to confirm microarray data following *SMC1B* depletion (primers listed in Supplementary Table S6). RT-qPCR was performed using QuantiTecT SYBR Green PCR mix (Qiagen) on the Rotor Gene 3000 (Corbett). Each sample was run in duplicate and repeated at least three times. Specific primers, were used to assay the relative enrichment and normalized with respect to *HPRT* and *Actin* for human and mouse cDNA respectively. The results are expressed as a fold enrichment relative to control cells.

### Agilent expression array hybridization

Total RNA from mock and *SMC1B* siRNA depleted human fibroblasts cells was extracted with the SV Total RNA Isolation System (Promega). Synthesis of cyanine-labeled cRNA was performed using Quick Amplification Labeling Two Color kit (Agilent) and hybridizations of the labeled cRNA were performed using oligo microarrays containing about 28,000 probe sets covering the whole human genome by Gene Expression Hybridization (Agilent). Images were quantified with Feature Extraction 10.5 (Agilent) and microarray data were normalized using locally weighted scatterplot smoothing (LOWESS) algorithm. Microarray data were screened according to expression level, fold change and standard deviation by GeneSpring 11.0 software. Student’s t-test was used to test for statistical significance in gene expression between mock and *SMC1B-*siRNA depleted human fibroblasts. A False Discovery Rate (Benjamini-Hochberg) was used to correct *p*-value. Data with a corrected *p*-value of <0.001 was considered statistically significant.

### Genome-wide localization of cohesin-SMC1B by ChIP-sequencing

ChIP was performed in human primary fibroblasts with custom rabbit polyclonal antibody against SMC1B. Cells were cross-linked by 1% formaldeide for 10 min at room temperature, resuspended in lysis buffer. Each sample was incubated with Dynabeads protein A (Invitrogen) previously bound with 10 ug of specific antibody. Next, the beads were washed with low salt buffer, high salt buffer and eluted overnight at 65 °C. The eluates were incubated with proteinase K and the purificate with QIAquick Purification Kit (Qiagen).

### Library preparation and sequencing

DNA recovered from the ChIP procedure was quantified using the Qubit 2.0 Fluorometer (Invitrogen) and the quality was tested by the Agilent 2100 Bioanalyzer (Agilent Technologies). The DNA was then processed, including end repair, adaptor ligation, and size selection, using an Ovation® Ultralow System V2 1–16 (Nugen) sample prep kit following the manufacturer’s instructions. Final DNA libraries were validated and processed with Illumina cBot for cluster generation on the flowcell, following the manufacturer’s instructions and sequenced on single-end 50 bp mode at the on HiSeq2500 (Illumina) at a depth of approximately 30–50 million sequences per sample. The CASAVA 1.8.2 version of the Illumina pipeline was used to processed raw data for both format conversion and de-multiplexing.

### ChIP-seq bioinformatics analysis

Raw sequence files were subjected to quality control analysis using FastQC (http://www.bioinformatics.babraham.ac.uk/projects/fastqc/). In order to avoid low quality data, adapters were removed by Cutadapt^56^ and lower quality bases were trimmed by ERNE such that the quality criteria of quality value per base (Phred score) was at least 35 and the read length was at least 30 bp. The quality-checked reads were mapped to the human reference genome GRCh37/hg19 using Bowtie 2.0.2^57^. Only uniquely mapping reads were used for the peak calling by MACS2^58^ with 0.05 FDR used as a cut-off value, and with standard parameters for shifting model calculations. MACS2 was also used for all comparisons between input track as control and each one of the data sets as treatment. Binding site overlaps between different sample sets were obtained using custom UNIX shell scripting. Genome-wide analysis of enrichment of chromosomal features and chromosomal distribution of ChIP regions were determined using CEAS package^43^.

ChIP-seq data was validated by RT-qPCR. Each sample was run in duplicate and repeated at least three times. Corresponding primers of the selected genes are described in Table S7. Input was determined relative to three genomic regions (chr5:140618351-140618463; chr9:116184241-116184471; chr11:8642689-8642807) that does not bind SMC1B.

Genome mapping of SMC1B binding regions were visualized using the UCSC interface (https://genome.ucsc.edu/).

### Accession

Next generation sequencing data are available on NCBI (SRP057544) DataSets.

### *SMC1B* mutation analysis

DNA was extracted from peripheral blood lymphocytes by a standard non-organic extraction procedure. All subjects negative for *NIPBL, SMC1A, HDAC8* and *RAD21* mutations were analyzed for *SMC1B*. This study was conducted according to the principles expressed in the Declaration of Helsinki. Written informed consents were obtained from all enrolled subjects. The study was approved by the IRB at The Children’s Hospital of Philadelphia.

Primer pairs (Supplementary Table S3) were designed to amplify exons, exon/intron boundaries and short flanking intronic sequences. Amplified PCR products were purified (Qiagen) and sequenced.

### Statistical analysis

Results were analyzed by Student’s *t*-test. *P*-values of <0.05 were considered statistically significant.

## Additional Information

**How to cite this article**: Mannini, L. *et al.* SMC1B is present in mammalian somatic cells and interacts with mitotic cohesin proteins. *Sci. Rep.* **5**, 18472; doi: 10.1038/srep18472 (2015).

## Supplementary Material

Supplementary Information

## Figures and Tables

**Table 1:** Number of aneuploid cells and spontaneous chromosome aberrations following siRNA treatment against *SMC1B*.

Treatment	Diploid cells	Aneuploid cells	Chromosome aberrations	No. cells
Mock	97	3	3	100
siRNA *SMC1B*	97	3	4	100

**Table 2:** Number of differentially expressed genes in *SMC1B* depleted cells.

	Total genes	Upregulated genes	Downregulated genes
Total probes	1209	99	1110
Non redundant-genes	1178	98	1080
