# Supplementary material for: SMC1B is present in mammalian somatic cells and interacts with mitotic cohesin proteins
Source: Sci Rep. 2015 Dec 17;5:18472. doi: 10.1038/srep18472 (PMC4682075; doi:10.1038/srep18472)
Supplement: Supplementary Information [file srep18472-s1.pdf]

Supplementary information

SMC1B is present in mammalian somatic cells and interacts with mitotic cohesin proteins

Linda Mannini, Francesco Cucco, Valentina Quarantotti, Clelia Amato, Mara Tinti, Luigi Tana,

Annalisa Frattini, Domenico Delia, Ian D. Krantz, Rolf Jessberger, Antonio Musio

Table S1. Alignment of human and mouse SMC1B protein sequences. In gray the amino acid sequence selected for the antibody generation.

```
hSMC1B    MAHLELLLVENFKSWRGRQVIGPFRRFTCIIGPNGSGKSNVMDALSFVMGEKIANLRVKN 60
mSMC1B    MGHLELLLVENFKSWRGRQVIGPFKRFTCIIGPNGSGKSNVMDALSFVMGEKTTNLRVKN 60
          *.**********************:*************************** :******

hSMC1B    IQELIHGAHIGKPISSSASVKIIYVEESGEEKTFARIIRGGCSEFRFNDNLVSRSVYIAE 120
mSMC1B    IQELIHGAHTGKPVSSSASVTIIYIEDSGEEKTFTRIIRGGCSEYHFGDKPVSRSVYVAQ 120
          ********* ***:******.***:*:*******:*********::*.*: ******:*:

hSMC1B    LEKIGIIVKAQNCLVFQGTVESISVKKPKERTQFFEEISTSGELIGEYEEKKRKLQKAEE 180
mSMC1B    LENIGIIVKAQNCLVFQGTVESISMKKPKERTQFFEEISTSGEFIGEYEAKKKKLQKAEE 180
          **:*********************:******************:***** **:*******

hSMC1B    DAQFNFNKKKNIAAERRQAKLEKEEAERYQSLLEELKMNKIQLQLFQLYHNEKKIHLLNT 240
mSMC1B    DAQFHFNVKKNVAAERKHAKIEKEEAEHYQNLLEELKINKIQLMLFQLYYNEEKINVLNT 240
          ****:** ***:****::**:******:**.******:***** *****:**:**::***

hSMC1B    KLEHVNRDLSVKRESLSHHENIVKARKKEHGMLTRQLQQTEKELKSVETLLNQKRPQYIK 300
mSMC1B    ELEQMDGNLSVVKDTLSHHENIFKAKKKDYGMLTRQLQQTAKELKSVEAILNQKRPQYIK 300
          :**::: :*** :::*******.**:**::********** *******::**********

hSMC1B    AKENTSHHLKKLDVAKKSIKDSEKQCSKQEDDIKALETELADLDAAWRSFEKQIEEEILH 360
mSMC1B    AKENTSHHLKKLDLSKKLITDNEKQCSKQEDGIRALVAELADLDRAWKSFEKQMEEKILQ 360
          *************::** *.*.*********.*:** :****** **:*****:**:**:

hSMC1B    KKRDIELEASQLDRYKELKEQVRKKVATMTQQLEKLQWEQKTDEERLAFEKRRHGEVQGN 420
mSMC1B    KGRDIELENSQLDRYKLLKEQVRRKVGIMTQQLEKLQWEQKAEKERLAFEKRRHGDTQGN 420
          * ****** ******* ******:**. *************:::***********:.***

hSMC1B    LKQIKEQIEDHKKRIEKLEEYTKTCMDCLKEKKQQEETLVDEIEKTKSRMSEFNEELNLI 480
mSMC1B    LKQIKEQIEEHKKRIEKLEEYTKTCMDCLEDKKQQEEALKKEIENTKSRMSEVNEELSLI 480
          *********:*******************::******:* .***:*******.****.**

hSMC1B    RSELQNAGIDTHEGKRQQKRAEVLEHLKRLYPDSVFGRLFDLCHPIHKKYQLAVTKVFGR 540
mSMC1B    RNELQNAGIDNHEGKRQQKRAEVLEHLKRLYPDSVFGRLLDLCHPIHKKYQLAVTKLFGR 540
          *.********.****************************:****************:***

hSMC1B    FITAIVVASEKVAKDCIRFLKEERAEPETFLALDYLDIKPINERLRELKGCKMVIDVIKT 600
mSMC1B    YMVAIVVASEKIAKDCIRFLKAERAEPETFLALDYLDIKPINERLREIKGCKMMIDVIKT 600
          ::.********:********* *************************:*****:******

hSMC1B    QFPQLKKVIQFVCGNGLVCETMEEARHIALSGPERQKTVALDGTLFLKSGVISGGSSDLK 660
mSMC1B    QFPQLKKVIQFVCGNGLVCETVEEARHIAFGGPERRKAVALDGTLFLKSGVISGGSSDLK 660
          *********************:*******:.****:*:**********************

hSMC1B    YKARCWDEKELKNLRDRRSQKIQELKGLMKTLRKETDLKQIQTLIQGTQTRLKYSQNELE 720
mSMC1B    HKALCWDEKELHNLRDKRSQLVQELKELMKTLRKETDLKQIQTLVQGTNTRLKYSQNELE 720
          :** *******:****:*** :**** *****************:***:***********

hSMC1B    MIKKKHLVAFYQEQSQLQSELLNIESQCIMLSEGIKERQRRIKEFQEKIDKVEDDIFQHF 780
mSMC1B    MIKKKHLATFYREQSQLQSELLNIDSQCTMLSEGINKQQQKIEEFQDKIDEVEDDIFQDF 780
          *******.:**:************:*** ******:::*::*:***:***:*******.*

hSMC1B    CEEIGVENIREFENKHVKRQQEIDQKRYFYKKMLTRLNVQLEYSRSHLKKKLNKINTLKE 840
mSMC1B    CEEIGVENIREFENKHVKQQQENDQKRLEFEKQKTRLNIQLEYSRNQLKKKLNNIDTLKT 840
          ******************:*** ****  ::*  ****:******.:******:*:*** 

hSMC1B    TIQKGSEDIDHLKKAEENCLQTVNELMAKQQQLKDIRVTQNSSAEKVQTQIEEERKKFLA 900
mSMC1B    TIQKGKEDIDNLKKTEEECLKIVEELMVKQEQIKEVLATQSSNIEKIHIQIEEERKKVLA 900
          *****.****:***:**:**: *:***.**:*:*:: .**.*.* **:: ********.**

hSMC1B    VDREVGKLQKEVVSIQTSLEQKRLEKHNLLLDCKVQDIEIILLSGSLDDIIEVEMGTEAE 960
mSMC1B    VDREVGKLQKEVVIIQGSLEQKLLEKHNLLLDCKVQDIDISLVLGSLEDIIEMEL-TETE 959
          ************* ** ***** ***************:* *: ***:****:*: **:*
```

```
hSMC1B    STQATIDIYEKEEAFEIDYSSLKEDLKALQSDQEIEAHLRLLLQQVASQEDILLKTAAPN 1020
mSMC1B    STQATADIYEKEASIQIDYSPLREDLKALQSDKEVEAHLTLLLQQVASQENTLLKTTAPN 1019
          ***** ****** :::****.*:*********:*:**** **********: ****:***

hSMC1B    LRALENLKTVRDKFQESTDAFEASRKEARLCRQEFEQVKKRRYDLFTQCFEHVSISIDQI 1080
mSMC1B    LRAQENLKTVRDKFQESADVFEASRKEARICRQEFEQVKRRRYDAFSQCFEHISVSIDQI 1079
          *** *************:*.*********:*********:**** *:*****:*:*****

hSMC1B    YKKLCRNNSAQAFLSPENPEEPYLEGISYNCVAPGKRFMPMDNLSGGEKCVAALALLFAV 1140
mSMC1B    YKKLCRNNSAQAFLSPENPEEPYLDGISYNCVAPGKRFMPMDNLSGGEKCVAALALLFAV 1139
          ************************:***********************************

hSMC1B    HSFRPAPFFVLDEVDAALDNTNIGKVSSYIKEQTQDQFQMIVISLKEEFYSRADALIGIY 1200
mSMC1B    HSFRPAPFFVLDEVDAALDNTNIGKVSSYIKEQSQEQFQMIIISLKEEFYSKADALIGVY 1199
          *********************************:*:*****:*********:******:*

hSMC1B    PEYDDCMFSRVLTLDLSQYPDTEGQESSKRHGESR-------------- 1235
mSMC1B    PEHNECMFSHVLTLDLSKYPDTEDQEGSRSHRKPRVPRVSMSPKSPQSR 1248
          **:::****:*******:*****.**.*: * :.*
```

Table S2. Dysregulated genes in *SMC1B* depleted cells by microarray analysis (p<0.001)

GeneSymbol	FC	P value	Description
POMC	-1,2142	3,26E-07	Homo sapiens proopiomelanocortin (POMC), transcript variant 1, mRNA [NM_001035256]
NOG	-1,27711	2,69E-06	Homo sapiens noggin (NOG), mRNA [NM_005450]
DLG1	-1,24231	6,04E-06	Homo sapiens discs, large homolog 1 (Drosophila) (DLG1), transcript variant 2, mRNA [NM_004087]
JAM2	-1,25326	6,55E-06	junctional adhesion molecule 2 [Source:HGNC Symbol;Acc:14686] [ENST00000480456]
PI4KB	-1,19037	7,71E-06	Homo sapiens phosphatidylinositol 4-kinase, catalytic, beta (PI4KB), transcript variant 2, mRNA [NM_001198773]
MYBL1	-1,46316	1,05E-05	Homo sapiens v-myb myeloblastosis viral oncogene homolog (avian)-like 1 (MYBL1), mRNA [NM_001144755]
TACO1	1,142974	1,23E-05	Homo sapiens translational activator of mitochondrially encoded cytochrome c oxidase I, mRNA [NM_016360]
RMND5B	1,181918	1,37E-05	Homo sapiens required for meiotic nuclear division 5 homolog B (S, cerevisiae) (RMND5B), mRNA [NM_022762]
PYCR1	-1,29733	1,37E-05	Homo sapiens pyrroline-5-carboxylate reductase 1 (PYCR1), transcript variant 2, mRNA [NM_153824]
MMP1	-2,14646	1,45E-05	Homo sapiens matrix metallopeptidase 1 (interstitial collagenase) (MMP1), mRNA [NM_002421]
C9orf142	1,218434	1,48E-05	Homo sapiens chromosome 9 open reading frame 142 (C9orf142), mRNA [NM_183241]
USP17L2	-4,45449	1,74E-05	Homo sapiens ubiquitin specific peptidase 17-like 2 (USP17L2), mRNA [NM_201402]
FAM55C	-1,45042	1,91E-05	Homo sapiens family with sequence similarity 55, member C (FAM55C), mRNA [NM_001134456]
HSD11B1L	1,115628	2,10E-05	Homo sapiens hydroxysteroid (11-beta) dehydrogenase 1-like (HSD11B1L), transcript variant b, mRNA [NM_198706]
RPL21	1,404046	2,21E-05	Homo sapiens ribosomal protein L21 (RPL21), mRNA [NM_000982]
LCE4A	-4,28966	2,47E-05	Homo sapiens late cornified envelope 4A (LCE4A), mRNA [NM_178356]
MIF	-2,13647	2,51E-05	Homo sapiens macrophage migration inhibitory factor (glycosylation-inhibiting factor) (MIF), mRNA [NM_002415]
BHLHA9	-1,75242	2,55E-05	Homo sapiens basic helix-loop-helix family, member a9 (BHLHA9), mRNA [NM_001164405]
C1orf129	-4,47018	2,65E-05	Homo sapiens chromosome 1 open reading frame 129 (C1orf129), transcript variant 2, mRNA [NM_025063]
RNF167	1,330603	2,74E-05	Homo sapiens ring finger protein 167 (RNF167), mRNA [NM_015528]
LOC646736	-4,37412	3,32E-05	Homo sapiens similar to Alu subfamily SX sequence contamination warning entry, mRNA, [BC017935]
C11orf30	-1,18021	3,34E-05	Homo sapiens chromosome 11 open reading frame 30 (C11orf30), mRNA [NM_020193]
ESR2	-4,38236	3,49E-05	Homo sapiens estrogen receptor 2 (ER beta) (ESR2), transcript variant e, mRNA [NM_001214903]
TOR1AIP2	-1,19708	3,93E-05	Homo sapiens torsin A interacting protein 2 (TOR1AIP2), transcript variant 2, mRNA [NM_145034]
RPS20P27	-1,83561	4,12E-05	Homo sapiens ribosomal protein S20 pseudogene 27, mRNA (cDNA clone IMAGE:5549882), [BC071734]
IDH3B	1,181201	4,13E-05	Homo sapiens isocitrate dehydrogenase 3 (NAD+) beta, mRNA [NM_174855]
C7orf33	-4,74353	4,19E-05	Homo sapiens chromosome 7 open reading frame 33 (C7orf33), mRNA [NM_145304]

C10orf105	-4,53274	4,28E-05	Homo sapiens chromosome 10 open reading frame 105 (C10orf105), transcript variant 1, mRNA [NM_001164375]
PAX6	-4,74989	4,45E-05	Homo sapiens paired box 6 (PAX6), transcript variant 1, mRNA [NM_000280]
FEZF2	-4,75437	4,53E-05	Homo sapiens FEZ family zinc finger 2 (FEZF2), mRNA [NM_018008]
LOC401022	-4,29437	4,87E-05	Homo sapiens uncharacterized LOC401022 (LOC401022), non-coding RNA [NR_033979]
SNX10	-4,74295	5,11E-05	Homo sapiens sorting nexin 10 (SNX10), transcript variant 1, mRNA [NM_001199835]
PDE8B	-1,82615	5,13E-05	Homo sapiens phosphodiesterase 8B (PDE8B), transcript variant 1, mRNA [NM_003719]
C1orf98	-4,76528	5,27E-05	Homo sapiens chromosome 1 open reading frame 98 (C1orf98), non-coding RNA [NR_040064]
RPL21	-1,7522	5,30E-05	Homo sapiens ribosomal protein L21 (RPL21), mRNA [NM_000982]
FAM149B1	-4,72018	5,49E-05	family with sequence similarity 149, member B1 [Source:HGNC Symbol;Acc:29162] [ENST00000242505]
ARMC7	1,228109	5,49E-05	Homo sapiens armadillo repeat containing 7 (ARMC7), mRNA [NM_024585]
RPL27	-1,93597	5,52E-05	Homo sapiens ribosomal protein L27 (RPL27), mRNA [NM_000988]
EIF3E	-4,6151	5,63E-05	eukaryotic translation initiation factor 3, subunit E [Source:HGNC Symbol;Acc:3277] [ENST00000522088]
EDDM3B	-4,30835	5,66E-05	Homo sapiens epididymal protein 3B (EDDM3B), mRNA [NM_022360]
RTN4	-1,98467	5,67E-05	Homo sapiens reticulon 4 (RTN4), transcript variant 1, mRNA [NM_020532]
LOC100128869	-5,52386	5,85E-05	PREDICTED: Homo sapiens hypothetical protein LOC100128869 (LOC100128869), mRNA [XM_001719518]
DSC3	-4,69784	5,91E-05	Homo sapiens desmocollin 3 (DSC3), transcript variant Dsc3b, mRNA [NM_024423]
FGF12	-4,72022	6,21E-05	Homo sapiens fibroblast growth factor 12 (FGF12), transcript variant 2, mRNA [NM_004113]
LOC284191	-3,83311	6,31E-05	Homo sapiens cDNA FLJ39879 fis, clone SPLEN2016069, [AK097198]
DISP2	-4,54053	6,38E-05	Homo sapiens mRNA; cDNA DKFZp761D0615 (from clone DKFZp761D0615), [AL359580]
LINC00281	-4,73019	6,38E-05	Homo sapiens long intergenic non-protein coding RNA 281 (LINC00281), non-coding RNA [NR_027278]
TIGD7	-1,2812	6,45E-05	Homo sapiens tigger transposable element derived 7 (TIGD7), mRNA [NM_033208]
PGK2	-4,23701	6,46E-05	Homo sapiens phosphoglycerate kinase 2 (PGK2), mRNA [NM_138733]
CYB5R3	-2,13493	6,49E-05	Homo sapiens cytochrome b5 reductase 3 (CYB5R3), transcript variant 2, mRNA [NM_007326]
LOC340239	-3,89217	6,70E-05	Homo sapiens cDNA clone IMAGE:30378049, [BC090890]
DIAPH3	-1,43572	6,73E-05	Homo sapiens diaphanous homolog 3 (Drosophila) (DIAPH3), transcript variant 1, mRNA [NM_001042517]
MED12L	-4,70994	6,77E-05	Homo sapiens mediator complex subunit 12-like (MED12L), mRNA [NM_053002]
COX7B2	-4,72895	6,80E-05	Homo sapiens cytochrome c oxidase subunit VIIb2 (COX7B2), mRNA [NM_130902]
ERBB4	-4,28766	6,97E-05	Homo sapiens v-erb-a erythroblastic leukemia viral oncogene homolog 4 (avian) (ERBB4), mRNA [NM_005235]
MEIS1	-4,47037	7,00E-05	Meis homeobox 1 [Source:HGNC Symbol;Acc:7000] [ENST00000409517]
FAM190A	-4,70903	7,01E-05	Homo sapiens family with sequence similarity 190, member A (FAM190A), transcript variant 2, mRNA [NM_207491]

GNAI2	-2,0756	7,45E-05	Homo sapiens guanine nucleotide binding protein (G protein) , mRNA [NM_002070]
RPL14	-2,10786	7,46E-05	Homo sapiens ribosomal protein L14 (RPL14), transcript variant 1, mRNA [NM_001034996]
SLC5A12	-4,72961	7,52E-05	Homo sapiens solute carrier family 5 (sodium/glucose cotransporter), member 12 (SLC5A12), mRNA [NM_178498]
TRA2B	-1,58507	7,53E-05	transformer 2 beta homolog (Drosophila) [Source:HGNC Symbol;Acc:10781] [ENST00000342294]
C10orf129	-4,51717	7,53E-05	chromosome 10 open reading frame 129 [Source:HGNC Symbol;Acc:31665] [ENST00000327739]
GCET2	-4,66875	7,56E-05	Homo sapiens germinal center expressed transcript 2 (GCET2), transcript variant 3, mRNA [NM_001190259]
LIPK	-4,67117	7,66E-05	Homo sapiens lipase, family member K (LIPK), mRNA [NM_001080518]
UGT2B4	-4,33387	7,71E-05	Homo sapiens UDP glucuronosyltransferase 2 family, polypeptide B4 (UGT2B4), mRNA [NM_021139]
C14orf105	-5,18444	7,84E-05	Homo sapiens chromosome 14 open reading frame 105 (C14orf105), mRNA [NM_018168]
FTSJD2	1,416657	7,91E-05	Homo sapiens FtsJ methyltransferase domain containing 2 (FTSJD2), mRNA [NM_015050]
GABRA1	-4,7481	8,14E-05	Homo sapiens gamma-aminobutyric acid (GABA) A receptor, alpha 1 (GABRA1), mRNA [NM_001127644]
C6orf10	-4,5427	8,14E-05	Homo sapiens chromosome 6 open reading frame 10 (C6orf10), mRNA [NM_006781]
C12orf39	-4,75357	8,17E-05	chromosome 12 open reading frame 39 [Source:HGNC Symbol;Acc:28139] [ENST00000256969]
SCN1A	-4,70488	8,29E-05	Homo sapiens sodium channel, voltage-gated, type I, alpha subunit (SCN1A), mRNA [NM_001202435]
RPS10	-2,0798	8,30E-05	Homo sapiens ribosomal protein S10 (RPS10), transcript variant 2, mRNA [NM_001014]
HTT	1,099624	8,39E-05	Homo sapiens huntingtin (HTT), mRNA [NM_002111]
SMCHD1	-4,6955	8,42E-05	Homo sapiens structural maintenance of chromosomes flexible hinge domain containing 1, mRNA [NM_015295]
LOC728175	-4,68855	8,52E-05	Homo sapiens uncharacterized LOC728175 (LOC728175), non-coding RNA [NR_040108]
OR5AN1	-4,6858	8,67E-05	Homo sapiens olfactory receptor, family 5, subfamily AN, member 1 (OR5AN1), mRNA [NM_001004729]
EEF1A1	-2,20205	8,79E-05	Homo sapiens eukaryotic translation elongation factor 1 alpha 1 (EEF1A1), mRNA [NM_001402]
RPL35A	-1,86915	8,80E-05	Homo sapiens ribosomal protein L35a (RPL35A), mRNA [NM_000996]
FAM163A	-4,24939	8,81E-05	Homo sapiens family with sequence similarity 163, member A (FAM163A), mRNA [NM_173509]
CES1P1	-4,72643	8,86E-05	Homo sapiens carboxylesterase 1 pseudogene 1 (CES1P1), non-coding RNA [NR_003276]
QKI	-4,47836	8,92E-05	Homo sapiens QKI, KH domain containing, RNA binding (QKI), transcript variant 1, mRNA [NM_006775]
LOC729176	-4,65636	8,96E-05	Homo sapiens chromosome 15 open reading frame 29 pseudogene (LOC729176), non-coding RNA [NR_003954]
LOC286359	-4,36914	9,04E-05	Homo sapiens uncharacterized LOC286359 (LOC286359), non-coding RNA [NR_026847]
C10orf92	-4,20181	9,04E-05	Homo sapiens chromosome 10 open reading frame 92 (C10orf92), mRNA [NM_001200049]
AP1S2	-4,67677	9,12E-05	adaptor-related protein complex 1, sigma 2 subunit [Source:HGNC Symbol;Acc:560] [ENST00000380291]
HERC3	-4,68065	9,15E-05	Homo sapiens hect domain and RLD 3, mRNA (cDNA clone IMAGE:6050308), complete cds, [BC038960]
DNAJB8-AS1	-4,6636	9,16E-05	Homo sapiens DNAJB8 antisense RNA 1 (non-protein coding) (DNAJB8-AS1), non-coding RNA [NR_037890]
AKR1D1	-4,7678	9,28E-05	Homo sapiens aldo-keto reductase family 1, member D1, mRNA [NM_005989]

NXPH1	-4,20958	9,34E-05	Homo sapiens neurexophilin 1 (NXPH1), mRNA [NM_152745]
TMEM150C	-2,44409	9,37E-05	Homo sapiens transmembrane protein 150C (TMEM150C), mRNA [NM_001080506]
SLC26A7	-4,66932	9,49E-05	Homo sapiens solute carrier family 26, member 7 (SLC26A7), transcript variant 1, mRNA [NM_052832]
MAGEB4	-4,66656	9,52E-05	Homo sapiens melanoma antigen family B, 4 (MAGEB4), mRNA [NM_002367]
UBE2D4	-1,1103	9,61E-05	Homo sapiens ubiquitin-conjugating enzyme E2D 4 (putative) (UBE2D4), mRNA [NM_015983]
LOC100128164	-4,73306	9,68E-05	Homo sapiens four and a half LIM domains 1 pseudogene (LOC100128164), non-coding RNA [NR_024409]
LOC157931	-4,67079	9,69E-05	Homo sapiens cDNA FLJ32712 fis, clone TESTI2000745, [AK057274]
CCDC70	-4,66595	9,70E-05	Homo sapiens coiled-coil domain containing 70 (CCDC70), mRNA [NM_031290]
ANKRD30A	-4,71709	9,78E-05	Homo sapiens ankyrin repeat domain 30A (ANKRD30A), mRNA [NM_052997]
DNAJC13	1,149457	9,80E-05	Homo sapiens DnaJ (Hsp40) homolog, subfamily C, member 13 (DNAJC13), mRNA [NM_015268]
RLN2	-4,60634	9,90E-05	Homo sapiens relaxin 2 (RLN2), transcript variant 2, mRNA [NM_005059]
EIF3L	1,29043	9,95E-05	Homo sapiens eukaryotic translation initiation factor 3, subunit L (EIF3L), transcript variant 1, mRNA [NM_016091]
PLEKHM2	-1,23908	9,95E-05	Homo sapiens pleckstrin homology domain containing, family M member 2, mRNA [NM_015164]
CHMP5	-1,24969	9,97E-05	Homo sapiens charged multivesicular body protein 5 (CHMP5), transcript variant 1, mRNA [NM_016410]
LOC644145	-4,67281	9,98E-05	Homo sapiens exocyst complex component 1 pseudogene (LOC644145), non-coding RNA [NR_003935]
ANGPTL3	-4,68002	1,00E-04	Homo sapiens angiopoietin-like 3 (ANGPTL3), mRNA [NM_014495]
SUSD4	-4,66041	1,01E-04	sushi domain containing 4 [Source:HGNC Symbol;Acc:25470] [ENST00000342943]
HSFY1P1	-4,66172	1,02E-04	Homo sapiens heat shock transcription factor, Y-linked 1 pseudogene 1 (HSFY1P1), non-coding RNA [NR_003607]
SLC12A5	-4,65825	1,02E-04	Homo sapiens solute carrier family 12 (potassium/chloride transporter), member 5 (SLC12A5), mRNA [NM_020708]
LGI2	-4,68813	1,02E-04	Homo sapiens leucine-rich repeat LGI family, member 2 (LGI2), mRNA [NM_018176]
FHL1	-4,65093	1,03E-04	four and a half LIM domains 1 [Source:HGNC Symbol;Acc:3702] [ENST00000370674]
LRP2BP	-4,66699	1,03E-04	Homo sapiens LRP2 binding protein (LRP2BP), mRNA [NM_018409]
GMDS	-4,69394	1,05E-04	GDP-mannose 4,6-dehydratase [Source:HGNC Symbol;Acc:4369] [ENST00000380805]
LIPF	-4,64446	1,05E-04	Homo sapiens lipase, gastric (LIPF), transcript variant 2, mRNA [NM_004190]
HFM1	-4,66837	1,06E-04	Homo sapiens HFM1, ATP-dependent DNA helicase homolog (S, cerevisiae) (HFM1), mRNA [NM_001017975]
UNQ6975	-4,389	1,06E-04	Homo sapiens putative uncharacterized protein UNQ6975/PRO21958 (UNQ6975), non-coding RNA [NR_033831]
LOC90834	-4,69172	1,06E-04	Homo sapiens uncharacterized protein BC001742 (LOC90834), non-coding RNA [NR_026993]
OR51F2	-4,65261	1,07E-04	Homo sapiens olfactory receptor, family 51, subfamily F, member 2 (OR51F2), mRNA [NM_001004753]
GABRA4	-4,6577	1,07E-04	Homo sapiens gamma-aminobutyric acid (GABA) A receptor, alpha 4 (GABRA4), mRNA [NM_000809]
CLVS1	-4,66217	1,08E-04	Homo sapiens clavesin 1 (CLVS1), mRNA [NM_173519]

MMP13	-4,66751	1,08E-04	Homo sapiens matrix metallopeptidase 13 (collagenase 3) (MMP13), mRNA [NM_002427]
LOC100129617	-4,62231	1,08E-04	Homo sapiens uncharacterized LOC100129617 (LOC100129617), non-coding RNA [NR_045112]
LOC286002	-4,65149	1,09E-04	Homo sapiens uncharacterized LOC286002 (LOC286002), non-coding RNA [NR_028137]
LHFPL1	-4,65037	1,09E-04	Homo sapiens lipoma HMGIC fusion partner-like 1 (LHFPL1), mRNA [NM_178175]
FAM150A	-4,33102	1,09E-04	Homo sapiens family with sequence similarity 150, member A (FAM150A), mRNA [NM_207413]
NDST3	-4,66454	1,09E-04	N-deacetylase/N-sulfotransferase (heparan glucosaminyl) 3 [Source:HGNC Symbol;Acc:7682] [ENST00000394488]
IGSF6	-4,20229	1,10E-04	Homo sapiens immunoglobulin superfamily, member 6 (IGSF6), mRNA [NM_005849]
LOC400568	-4,65031	1,10E-04	Homo sapiens cDNA clone IMAGE:5176687, [BC043554]
KIAA1239	-4,63677	1,11E-04	Homo sapiens KIAA1239 (KIAA1239), mRNA [NM_001144990]
SLITRK5	-4,65103	1,11E-04	Homo sapiens SLIT and NTRK-like family, member 5 (SLITRK5), mRNA [NM_015567]
CXCL1	-2,03445	1,11E-04	Homo sapiens chemokine (C-X-C motif) ligand 1 (melanoma growth stimulating activity, alpha), mRNA [NM_001511]
KIF1C	1,316106	1,11E-04	Homo sapiens kinesin family member 1C (KIF1C), mRNA [NM_006612]
TTN	-3,93242	1,11E-04	Homo sapiens titin (TTN), transcript variant novex-3, mRNA [NM_133379]
TMEM59	-4,67029	1,11E-04	transmembrane protein 59 [Source:HGNC Symbol;Acc:1239] [ENST00000371337]
WDR17	-4,70111	1,12E-04	Homo sapiens WD repeat domain 17 (WDR17), transcript variant 1, mRNA [NM_170710]
CALHM2	-4,12669	1,12E-04	calcium homeostasis modulator 2 [Source:HGNC Symbol;Acc:23493] [ENST00000393235]
TMPRSS11A	-4,64706	1,12E-04	Homo sapiens transmembrane protease, serine 11A (TMPRSS11A), transcript variant 1, mRNA [NM_182606]
UGT2A3	-4,66242	1,13E-04	Homo sapiens UDP glucuronosyltransferase 2 family, polypeptide A3 (UGT2A3), mRNA [NM_024743]
GDI2	-4,68644	1,14E-04	GDP dissociation inhibitor 2 [Source:HGNC Symbol;Acc:4227] [ENST00000380127]
CSRP2BP	-1,19466	1,14E-04	Homo sapiens CSRP2 binding protein (CSRP2BP), transcript variant 1, mRNA [NM_020536]
GYPA	-4,66592	1,14E-04	Homo sapiens glycophorin A (MNS blood group) (GYPA), mRNA [NM_002099]
MED14	-4,25024	1,15E-04	mediator complex subunit 14 [Source:HGNC Symbol;Acc:2370] [ENST00000324817]
SNORA77	-4,19151	1,16E-04	DB361496 MAMMA1 Homo sapiens cDNA clone MAMMA1001810 3', mRNA sequence [DB361496]
SGMS2	1,068424	1,16E-04	Homo sapiens sphingomyelin synthase 2 (SGMS2), transcript variant 1, mRNA [NM_152621]
RIMS2	-4,17923	1,16E-04	Homo sapiens regulating synaptic membrane exocytosis 2 (RIMS2), transcript variant 2, mRNA [NM_014677]
NPAS3	-4,64548	1,16E-04	Homo sapiens neuronal PAS domain protein 3 (NPAS3), transcript variant 1, mRNA [NM_001164749]
MSH4	-4,33	1,16E-04	Homo sapiens mutS homolog 4 (E, coli) (MSH4), mRNA [NM_002440]
SH2D1A	-4,66489	1,17E-04	Homo sapiens SH2 domain containing 1A (SH2D1A), transcript variant 1, mRNA [NM_002351]
OR6S1	-4,5504	1,17E-04	Homo sapiens olfactory receptor, family 6, subfamily S, member 1 (OR6S1), mRNA [NM_001001968]
SMOC2	-4,34053	1,17E-04	Homo sapiens SPARC related modular calcium binding 2 (SMOC2), transcript variant 1, mRNA [NM_022138]

LOC128322	-2,45878	1,17E-04	PREDICTED: Homo sapiens nuclear transport factor 2-like (LOC128322), mRNA [XM_001716411]
TRIM35	1,25975	1,18E-04	Homo sapiens tripartite motif containing 35 (TRIM35), transcript variant 2, mRNA [NM_171982]
SEC14L5	-4,18402	1,18E-04	Homo sapiens SEC14-like 5 (S, cerevisiae) (SEC14L5), mRNA [NM_014692]
ARL13A	-4,64485	1,18E-04	Homo sapiens ADP-ribosylation factor-like 13A (ARL13A), transcript variant 1, mRNA [NM_001162490]
CUBN	-5,16006	1,18E-04	cubilin (intrinsic factor-cobalamin receptor) [Source:HGNC Symbol;Acc:2548] [ENST00000377823]
C10orf140	-4,09912	1,18E-04	Homo sapiens chromosome 10 open reading frame 140 (C10orf140), mRNA [NM_207371]
TRPV4	-1,51024	1,18E-04	Homo sapiens transient receptor potential cation channel, subfamily V, member 4 (TRPV4), mRNA [NM_147204]
C6orf195	-4,74223	1,19E-04	Homo sapiens chromosome 6 open reading frame 195 (C6orf195), mRNA [NM_152554]
LOC100131497	-4,64128	1,19E-04	human full-length cDNA 5-PRIME end of clone CS0CAP002YE20 of Thymus of Homo sapiens (human), [BX248745]
PKP2	-4,08355	1,20E-04	Homo sapiens plakophilin 2 (PKP2), transcript variant 2b, mRNA [NM_004572]
CADM2	-4,24665	1,20E-04	Homo sapiens cell adhesion molecule 2 (CADM2), transcript variant 1, mRNA [NM_001167674]
OR10V1	-4,64317	1,21E-04	Homo sapiens olfactory receptor, family 10, subfamily V, member 1 (OR10V1), mRNA [NM_001005324]
TCTA	1,241441	1,21E-04	Homo sapiens T-cell leukemia translocation altered gene (TCTA), mRNA [NM_022171]
FAM110C	-4,64084	1,21E-04	Homo sapiens family with sequence similarity 110, member C (FAM110C), mRNA [NM_001077710]
TTTY9A	-4,68781	1,22E-04	Homo sapiens testis-specific transcript, Y-linked 9A (non-protein coding) (TTTY9A), non-coding RNA [NR_001530]
C15orf2	-4,65683	1,22E-04	Homo sapiens chromosome 15 open reading frame 2 (C15orf2), mRNA [NM_018958]
GSTA7P	-4,64182	1,22E-04	Homo sapiens glutathione S-transferase alpha 7, pseudogene (GSTA7P), non-coding RNA [NR_033760]
GPR64	-4,08463	1,22E-04	Homo sapiens G protein-coupled receptor 64 (GPR64), transcript variant 1, mRNA [NM_001079858]
RPL37A	-1,75509	1,23E-04	Homo sapiens ribosomal protein L37a (RPL37A), mRNA [NM_000998]
LOC400655	-4,67425	1,23E-04	Homo sapiens cDNA clone IMAGE:4825594, [BC047643]
C17orf104	-4,67613	1,24E-04	Homo sapiens chromosome 17 open reading frame 104 (C17orf104), mRNA [NM_001145080]
CCDC141	-4,63953	1,24E-04	coiled-coil domain containing 141 [Source:HGNC Symbol;Acc:26821] [ENST00000295723]
PLN	-4,64118	1,24E-04	Homo sapiens phospholamban (PLN), mRNA [NM_002667]
RPS29	-1,32037	1,25E-04	Homo sapiens ribosomal protein S29 (RPS29), transcript variant 2, mRNA [NM_001030001]
PIRT	-4,64549	1,25E-04	Homo sapiens phosphoinositide-interacting regulator of transient receptor potential channels, mRNA [NM_001101387]
GSTTP1	-4,19869	1,25E-04	Homo sapiens glutathione S-transferase theta pseudogene 1 (GSTTP1), non-coding RNA [NR_003081]
SLC5A10	-1,77304	1,26E-04	Homo sapiens solute carrier family 5 (sodium/glucose cotransporter), member 10 (SLC5A10), mRNA [NM_152351]
LOC400654	-4,10396	1,27E-04	Homo sapiens uncharacterized LOC400654 (LOC400654), non-coding RNA [NR_033983]
MYH1	-4,63922	1,28E-04	Homo sapiens myosin, heavy chain 1, skeletal muscle, adult (MYH1), mRNA [NM_005963]
OR4C11	-4,63551	1,29E-04	Homo sapiens olfactory receptor, family 4, subfamily C, member 11 (OR4C11), mRNA [NM_001004700]
TBC1D3B	-4,15498	1,29E-04	Homo sapiens TBC1 domain family, member 3B (TBC1D3B), mRNA [NM_001001417]

RPL35	-1,69858	1,30E-04	Homo sapiens ribosomal protein L35 (RPL35), mRNA [NM_007209]
SMEK3P	-5,19232	1,30E-04	Homo sapiens SMEK homolog 3, suppressor of mek1 (Dictyostelium) pseudogene, non-coding RNA [NR_002784]
IFNA14	-4,34655	1,31E-04	Homo sapiens interferon, alpha 14 (IFNA14), mRNA [NM_002172]
CACNB4	-4,17285	1,32E-04	Homo sapiens calcium channel, voltage-dependent, beta 4 subunit (CACNB4), mRNA [NM_001005747]
AMOT	-4,68419	1,33E-04	Homo sapiens angiomotin (AMOT), transcript variant 1, mRNA [NM_001113490]
LOC100131354	-4,43165	1,33E-04	PREDICTED: Homo sapiens hypothetical protein LOC100131354 (LOC100131354), mRNA [XM_001719406]
ARMCX6	1,323627	1,33E-04	Homo sapiens armadillo repeat containing, X-linked 6 (ARMCX6), transcript variant 1, mRNA [NM_019007]
RNF17	-4,66141	1,34E-04	Homo sapiens ring finger protein 17 (RNF17), transcript variant 1, mRNA [NM_031277]
NEUROG2	-4,66893	1,34E-04	Homo sapiens neurogenin 2 (NEUROG2), mRNA [NM_024019]
A2M	1,359791	1,34E-04	Homo sapiens alpha-2-macroglobulin (A2M), mRNA [NM_000014]
PROM1	-5,10416	1,34E-04	Homo sapiens prominin 1 (PROM1), transcript variant 6, mRNA [NM_001145850]
MARVELD3	-4,25369	1,35E-04	Homo sapiens MARVEL domain containing 3 (MARVELD3), transcript variant 2, mRNA [NM_052858]
PCDP1	-4,63198	1,35E-04	Homo sapiens primary ciliary dyskinesia protein 1 (PCDP1), mRNA [NM_001029996]
SCGB2A1	-5,2232	1,35E-04	Homo sapiens secretoglobin, family 2A, member 1 (SCGB2A1), mRNA [NM_002407]
LOC727915	-4,60245	1,35E-04	AGENCOURT_14276929 NIH_MGC_180 Homo sapiens , mRNA sequence [CD359326]
SRG7	-4,38193	1,36E-04	Homo sapiens spermatogenesis-related protein 7 (SRG7), non-coding RNA [NR_034168]
ADAM30	-4,63182	1,36E-04	Homo sapiens ADAM metallopeptidase domain 30 (ADAM30), mRNA [NM_021794]
KLB	-4,6248	1,36E-04	Homo sapiens klotho beta (KLB), mRNA [NM_175737]
NUDT16P1	-5,17341	1,37E-04	Homo sapiens nudix (nucleoside diphosphate linked moiety X)-type motif 16 pseudogene 1 [NR_002949]
FTH1	-1,91821	1,37E-04	Homo sapiens ferritin, heavy polypeptide 1 (FTH1), mRNA [NM_002032]
CCNC	-4,65901	1,37E-04	Homo sapiens cyclin C, mRNA (cDNA clone IMAGE:4250420), partial cds, [BC026272]
LOC100128699	-4,111	1,38E-04	Homo sapiens cDNA FLJ43882 fis, clone TESTI4009034, [AK125870]
PAK7	-4,63006	1,39E-04	Homo sapiens p21 protein (Cdc42/Rac)-activated kinase 7 (PAK7), transcript variant 1, mRNA [NM_020341]
OR8B2	-4,71499	1,39E-04	Homo sapiens olfactory receptor, family 8, subfamily B, member 2 (OR8B2), mRNA [NM_001005468]
C12orf39	-5,12101	1,40E-04	Homo sapiens chromosome 12 open reading frame 39 (C12orf39), mRNA [NM_030572]
COL11A1	-5,20081	1,41E-04	Homo sapiens collagen, type XI, alpha 1 (COL11A1), transcript variant B, mRNA [NM_080629]
TCF7L2	-4,79166	1,41E-04	Homo sapiens transcription factor 7-like 2 (T-cell specific, HMG-box) (TCF7L2), mRNA [NM_030756]
GREB1	-4,6906	1,41E-04	Homo sapiens growth regulation by estrogen in breast cancer 1 (GREB1), transcript variant a, mRNA [NM_014668]
MIR600HG	-4,69292	1,42E-04	Homo sapiens MIR600 host gene (non-protein coding) (MIR600HG), non-coding RNA [NR_026677]
EFCAB9	3,738979	1,43E-04	Homo sapiens EF-hand calcium binding domain 9 (EFCAB9), mRNA [NM_001171183]
BRWD1	-4,01707	1,44E-04	Homo sapiens bromodomain and WD repeat domain containing 1 (BRWD1), mRNA [NM_033656]

ZNF676	-4,63392	1,44E-04	Homo sapiens zinc finger protein 676 (ZNF676), mRNA [NM_001001411]
ZNF644	-1,53531	1,46E-04	Homo sapiens zinc finger protein 644 (ZNF644), transcript variant 1, mRNA [NM_201269]
L3MBTL4	-4,54916	1,46E-04	Homo sapiens l(3)mbt-like 4 (Drosophila) (L3MBTL4), mRNA [NM_173464]
MTFP1	1,320967	1,46E-04	Homo sapiens mitochondrial fission process 1 , nuclear gene encoding mitochondrial protein,mRNA [NM_016498]
OR52N4	-4,63912	1,47E-04	Homo sapiens olfactory receptor, family 52, subfamily N, member 4 (OR52N4), mRNA [NM_001005175]
TPI1	1,29383	1,48E-04	Homo sapiens triosephosphate isomerase 1 (TPI1), transcript variant 1, mRNA [NM_000365]
BEND2	-4,62987	1,49E-04	Homo sapiens BEN domain containing 2 (BEND2), transcript variant 1, mRNA [NM_153346]
LMO3	-5,1672	1,49E-04	Homo sapiens LIM domain only 3 (rhombotin-like 2) (LMO3), transcript variant 1, mRNA [NM_018640]
EWSR1	-1,14548	1,49E-04	Homo sapiens Ewing sarcoma breakpoint region 1 (EWSR1), transcript variant 1, mRNA [NM_013986]
SERPINB12	-3,94905	1,50E-04	Homo sapiens serpin peptidase inhibitor, clade B (ovalbumin), member 12 (SERPINB12), mRNA [NM_080474]
DNAJB7	-4,62503	1,50E-04	Homo sapiens DnaJ (Hsp40) homolog, subfamily B, member 7 (DNAJB7), mRNA [NM_145174]
OR6F1	-4,67959	1,51E-04	Homo sapiens olfactory receptor, family 6, subfamily F, member 1 (OR6F1), mRNA [NM_001005286]
LOC339260	-5,08522	1,52E-04	Homo sapiens cDNA clone IMAGE:5494257, partial cds, [BC041488]
ABHD13	-4,66643	1,52E-04	Homo sapiens abhydrolase domain containing 13 (ABHD13), mRNA [NM_032859]
GUCY1A3	-4,6621	1,52E-04	Homo sapiens guanylate cyclase 1, soluble, alpha 3 (GUCY1A3), transcript variant 6, mRNA [NM_001130686]
REG1P	-4,17595	1,52E-04	Homo sapiens regenerating islet-derived 1 pseudogene (REG1P), non-coding RNA [NR_002714]
IL2	-4,31745	1,53E-04	Homo sapiens interleukin 2 (IL2), mRNA [NM_000586]
TF	-4,66166	1,54E-04	Homo sapiens transferrin (TF), mRNA [NM_001063]
LMNA	-2,23608	1,54E-04	Homo sapiens lamin A/C (LMNA), transcript variant 2, mRNA [NM_005572]
LOC100505695	-5,0889	1,54E-04	PREDICTED: Homo sapiens hypothetical LOC100505695 (LOC100505695), miscRNA [XR_132489]
ZPBP2	-4,62455	1,56E-04	Homo sapiens zona pellucida binding protein 2 (ZPBP2), transcript variant 2, mRNA [NM_199321]
TEX11	-4,15173	1,56E-04	Homo sapiens testis expressed 11 (TEX11), transcript variant 1, mRNA [NM_001003811]
PCDH17	-4,38186	1,57E-04	Homo sapiens protocadherin 17 (PCDH17), mRNA [NM_001040429]
CXXC4	-4,18619	1,57E-04	Homo sapiens CXXC finger protein 4 (CXXC4), mRNA [NM_025212]
OR52B6	-4,6924	1,59E-04	Homo sapiens olfactory receptor, family 52, subfamily B, member 6 (OR52B6), mRNA [NM_001005162]
ISG15	-2,25492	1,59E-04	Homo sapiens ISG15 ubiquitin-like modifier (ISG15), mRNA [NM_005101]
SLC35B4	-1,27597	1,60E-04	Homo sapiens solute carrier family 35, member B4 (SLC35B4), mRNA [NM_032826]
SVIP	-4,62647	1,61E-04	Homo sapiens small VCP/p97-interacting protein (SVIP), mRNA [NM_148893]
PRPF4B	1,201503	1,62E-04	Homo sapiens PRP4 pre-mRNA processing factor 4 homolog B (yeast) (PRPF4B), mRNA [NM_003913]
OR10S1	-4,50095	1,62E-04	Homo sapiens olfactory receptor, family 10, subfamily S, member 1 (OR10S1), mRNA [NM_001004474]
LOC100131599	-4,64777	1,62E-04	Homo sapiens cDNA FLJ44233 fis, clone THYMU3006963, [AK126221]

CR1L	-4,71486	1,63E-04	Homo sapiens complement component (3b/4b) receptor 1-like (CR1L), mRNA [NM_175710]
ZFP28	-4,65807	1,63E-04	Homo sapiens zinc finger protein 28 homolog (mouse) (ZFP28), mRNA [NM_020828]
GAPDHS	-4,61871	1,64E-04	Homo sapiens glyceraldehyde-3-phosphate dehydrogenase, spermatogenic (GAPDHS), mRNA [NM_014364]
C6orf103	-4,8092	1,64E-04	Homo sapiens chromosome 6 open reading frame 103 (C6orf103), mRNA [NM_024694]
GAD2	-5,17358	1,64E-04	Homo sapiens glutamate decarboxylase 2 (pancreatic islets and brain, 65kDa) (GAD2), mRNA [NM_001134366]
HERC3	-4,62032	1,65E-04	Homo sapiens hect domain and RLD 3 (HERC3), mRNA [NM_014606]
TMEM56	-4,6406	1,66E-04	Homo sapiens transmembrane protein 56 (TMEM56), transcript variant 1, mRNA [NM_001199679]
PSMG4	-4,74345	1,66E-04	Homo sapiens cDNA FLJ38364 fis, clone FEBRA2000909, [AK095683]
AMZ2P1	-4,58549	1,68E-04	Homo sapiens archaelysin family metallopeptidase 2 pseudogene 1 (AMZ2P1), non-coding RNA [NR_026903]
PDRG1	1,291006	1,69E-04	Homo sapiens p53 and DNA-damage regulated 1 (PDRG1), mRNA [NM_030815]
TCL6	-4,01928	1,70E-04	Homo sapiens T-cell leukemia/lymphoma 6, mRNA, complete cds, [BC041075]
HBS1L	-4,61523	1,70E-04	Homo sapiens HBS1-like (S, cerevisiae) (HBS1L), transcript variant 1, mRNA [NM_006620]
PAX9	-4,6285	1,70E-04	Homo sapiens paired box 9 (PAX9), mRNA [NM_006194]
LLGL1	-5,15459	1,71E-04	Homo sapiens lethal giant larvae homolog 1 (Drosophila) (LLGL1), mRNA [NM_004140]
ABLIM2	-5,16411	1,71E-04	Homo sapiens actin binding LIM protein family, member 2 (ABLIM2), transcript variant 7, mRNA [NM_001130088]
OR4K13	-4,62979	1,72E-04	Homo sapiens olfactory receptor, family 4, subfamily K, member 13 (OR4K13), mRNA [NM_001004714]
KCNA2	-4,64143	1,72E-04	Homo sapiens potassium voltage-gated channel, shaker-related subfamily, member 2, mRNA [NM_001204269]
LOC439950	-4,63763	1,73E-04	Homo sapiens cDNA clone IMAGE:4826362, [BC046381]
POU2AF1	-4,62281	1,74E-04	Homo sapiens POU class 2 associating factor 1 (POU2AF1), mRNA [NM_006235]
FLJ26245	-4,65831	1,74E-04	Homo sapiens uncharacterized LOC400533 (FLJ26245), non-coding RNA [NR_033985]
MARCH1	-4,62879	1,76E-04	Homo sapiens membrane-associated ring finger (C3HC4) 1 (MARCH1), transcript variant 2, mRNA [NM_017923]
C17orf57	-4,6949	1,76E-04	Homo sapiens chromosome 17 open reading frame 57 (C17orf57), transcript variant B, mRNA [NM_001195192]
CD69	-4,26422	1,76E-04	Homo sapiens CD69 molecule (CD69), transcript variant 1, mRNA [NM_001781]
LOC100133106	-4,61852	1,76E-04	PREDICTED: Homo sapiens VCEW9374 (LOC100133106), miscRNA [XR_110065]
GKN2	-4,07016	1,77E-04	Homo sapiens gastrokine 2 (GKN2), mRNA [NM_182536]
LOC348761	-4,66419	1,78E-04	Homo sapiens uncharacterized LOC348761 (LOC348761), non-coding RNA [NR_033879]
LOC100134167	-4,64625	1,78E-04	PREDICTED: Homo sapiens uncharacterized protein C2orf27-like (LOC100134167), miscRNA [XR_133543]
LOC645485	-4,73531	1,79E-04	Homo sapiens, clone IMAGE:5741993, mRNA, [BC039526]
SCGB1D4	-4,61228	1,79E-04	Homo sapiens secretoglobin, family 1D, member 4 (SCGB1D4), mRNA [NM_206998]
GATA4	-5,15252	1,80E-04	Homo sapiens GATA binding protein 4 (GATA4), mRNA [NM_002052]
LOXHD1	-4,78908	1,80E-04	Homo sapiens lipoxygenase homology domains 1 (LOXHD1), transcript variant 2, mRNA [NM_001145472]

NKAIN3	-4,63163	1,80E-04	Homo sapiens Na+/K+ transporting ATPase interacting 3 (NKAIN3), mRNA [NM_173688]
MED29	-4,68371	1,80E-04	Homo sapiens mediator complex subunit 29 (MED29), mRNA [NM_017592]
GIMAP1	-4,64277	1,81E-04	Homo sapiens GTPase, IMAP family member 1 (GIMAP1), mRNA [NM_130759]
GLRX5	1,113632	1,82E-04	Homo sapiens glutaredoxin 5 (GLRX5), nuclear gene encoding mitochondrial protein, mRNA [NM_016417]
KCNA1	-4,65696	1,82E-04	Homo sapiens potassium voltage-gated channel, shaker-related subfamily, mRNA [NM_000217]
MS4A8B	-4,60738	1,82E-04	Homo sapiens membrane-spanning 4-domains, subfamily A, member 8B (MS4A8B), mRNA [NM_031457]
SULT6B1	-4,65083	1,83E-04	Homo sapiens sulfotransferase family, cytosolic, 6B, member 1 (SULT6B1), mRNA [NM_001032377]
USH2A	-3,31469	1,83E-04	Homo sapiens Usher syndrome 2A (autosomal recessive, mild) (USH2A), transcript variant 1, mRNA [NM_007123]
LOC400927	-4,10359	1,83E-04	Homo sapiens cDNA FLJ42070 fis, clone SYNOV2012326, [AK124064]
LOC100190938	-5,1471	1,84E-04	Homo sapiens uncharacterized LOC100190938 (LOC100190938), transcript variant 2, non-coding RNA [NR_024462]
CRB2	-5,13715	1,84E-04	crumbs homolog 2 (Drosophila) [Source:HGNC Symbol;Acc:18688] [ENST00000359999]
KLRC3	-4,16539	1,84E-04	Homo sapiens killer cell lectin-like receptor subfamily C, member 3 (KLRC3), mRNA [NM_007333]
MRPL30	-4,31468	1,85E-04	mitochondrial ribosomal protein L30 [Source:HGNC Symbol;Acc:14036] [ENST00000409145]
NSD1	-4,57755	1,86E-04	Homo sapiens nuclear receptor binding SET domain protein 1 (NSD1), transcript variant 2, mRNA [NM_022455]
RNF167	1,260713	1,87E-04	Homo sapiens ring finger protein 167 (RNF167), mRNA [NM_015528]
NDUFV2	1,113534	1,87E-04	Homo sapiens NADH dehydrogenase (ubiquinone) flavoprotein 2, 24kDa, mRNA [NM_021074]
ZNF648	-4,60155	1,88E-04	Homo sapiens zinc finger protein 648 (ZNF648), mRNA [NM_001009992]
KIAA1841	-1,27275	1,89E-04	Homo sapiens KIAA1841 (KIAA1841), transcript variant 2, mRNA [NM_032506]
C1orf150	-4,62028	1,89E-04	Homo sapiens chromosome 1 open reading frame 150 (C1orf150), mRNA [NM_145278]
SREBF2	-1,13159	1,89E-04	Homo sapiens sterol regulatory element binding transcription factor 2 (SREBF2), mRNA [NM_004599]
GOSR2	-1,25392	1,91E-04	Homo sapiens golgi SNAP receptor complex member 2 (GOSR2), transcript variant A, mRNA [NM_004287]
TSSC1	-4,76626	1,91E-04	tumor suppressing subtransferable candidate 1 [Source:HGNC Symbol;Acc:12383] [ENST00000443925]
C12orf48	-4,65107	1,92E-04	Homo sapiens chromosome 12 open reading frame 48, mRNA [BC018903]
RPL23	-2,00919	1,92E-04	Homo sapiens ribosomal protein L23 (RPL23), mRNA [NM_000978]
LAMP1	-1,87987	1,92E-04	Homo sapiens lysosomal-associated membrane protein 1 (LAMP1), mRNA [NM_005561]
PAK1IP1	1,186723	1,92E-04	Homo sapiens PAK1 interacting protein 1 (PAK1IP1), mRNA [NM_017906]
SHARPIN	-1,14667	1,93E-04	Homo sapiens SHANK-associated RH domain interactor (SHARPIN), transcript variant 1, mRNA [NM_030974]
MMP3	-2,03032	1,93E-04	Homo sapiens matrix metallopeptidase 3 (stromelysin 1, progelatinase) (MMP3), mRNA [NM_002422]
ADH1B	-4,73038	1,96E-04	Homo sapiens alcohol dehydrogenase 1B (class I), beta polypeptide (ADH1B), mRNA [NM_000668]
LINC00032	-4,61411	1,96E-04	Homo sapiens long intergenic non-protein coding RNA 32 (LINC00032), non-coding RNA [NR_026679]
BRI3	-1,87181	1,96E-04	Homo sapiens brain protein I3 (BRI3), transcript variant 1, mRNA [NM_015379]

KCNH2	-4,77331	1,98E-04	Homo sapiens potassium voltage-gated channel, subfamily H (eag-related), member 2, mRNA [NM_000238]
GEMIN8P4	-4,62972	1,98E-04	Homo sapiens gem (nuclear organelle) associated protein 8 pseudogene 4 (GEMIN8P4) [NR_002830]
ITIH5	-4,30875	1,98E-04	Homo sapiens inter-alpha-trypsin inhibitor heavy chain family, member 5 (ITIH5), mRNA [NM_001001851]
LINC00052	-4,61391	1,99E-04	Homo sapiens long intergenic non-protein coding RNA 52 (LINC00052), non-coding RNA [NR_026869]
MICU1	-5,12038	1,99E-04	mitochondrial calcium uptake 1 [Source:HGNC Symbol;Acc:1530] [ENST00000476605]
TAAR9	-4,64593	1,99E-04	Homo sapiens trace amine associated receptor 9 (gene/pseudogene) (TAAR9), mRNA [NM_175057]
HOXB2	-1,53011	1,99E-04	Homo sapiens homeobox B2 (HOXB2), mRNA [NM_002145]
LOC285181	-4,73496	1,99E-04	Homo sapiens cDNA FLJ36538 fis, clone TRACH2005159, [AK093857]
MYO3B	-4,67024	1,99E-04	Homo sapiens myosin IIIB (MYO3B), transcript variant 2, mRNA [NM_138995]
OR5B12	-4,67915	2,00E-04	Homo sapiens olfactory receptor, family 5, subfamily B, member 12 (OR5B12), mRNA [NM_001004733]
PLCZ1	-4,15357	2,00E-04	Homo sapiens phospholipase C, zeta 1 (PLCZ1), mRNA [NM_033123]
ATG4C	-5,1233	2,00E-04	Homo sapiens ATG4 autophagy related 4 homolog C (S, cerevisiae) (ATG4C), mRNA [NM_032852]
TBPL1	1,26387	2,01E-04	Homo sapiens TBP-like 1 (TBPL1), mRNA [NM_004865]
DNAJC3	-4,63016	2,01E-04	Homo sapiens DnaJ (Hsp40) homolog, subfamily C, member 3, mRNA (cDNA clone IMAGE:5218144), [BC033823]
LOC400643	-4,20299	2,01E-04	Homo sapiens uncharacterized LOC400643 (LOC400643), non-coding RNA [NR_034100]
GRIP2	-4,672	2,02E-04	Homo sapiens glutamate receptor interacting protein 2 (GRIP2), mRNA [NM_001080423]
TEX9	-5,12421	2,02E-04	Homo sapiens testis expressed 9 (TEX9), mRNA [NM_198524]
CTNNA3	-4,6465	2,02E-04	catenin (cadherin-associated protein), alpha 3 [Source:HGNC Symbol;Acc:2511] [ENST00000472963]
QRSL1	-5,11134	2,03E-04	glutaminyl-tRNA synthase (glutamine-hydrolyzing)-like 1 [Source:HGNC Symbol;Acc:21020] [ENST00000369044]
LOC286149	-4,69033	2,04E-04	PREDICTED: Homo sapiens hypothetical LOC286149 (LOC286149), miscRNA [XR_132545]
ARID3A	-1,46793	2,04E-04	Homo sapiens AT rich interactive domain 3A (BRIGHT-like) (ARID3A), mRNA [NM_005224]
MAGEB6	-4,73205	2,05E-04	Homo sapiens melanoma antigen family B, 6 (MAGEB6), mRNA [NM_173523]
TBP	1,116009	2,06E-04	Homo sapiens TATA box binding protein (TBP), transcript variant 1, mRNA [NM_003194]
GNGT1	-4,35406	2,06E-04	Homo sapiens guanine nucleotide binding protein, gamma transducing activity polypeptide 1 , mRNA [NM_021955]
RAD9B	-5,09502	2,06E-04	Homo sapiens RAD9 homolog B (S, pombe) (RAD9B), mRNA [NM_152442]
SLC26A8	-3,97838	2,07E-04	Homo sapiens solute carrier family 26, member 8 (SLC26A8), transcript variant 1, mRNA [NM_052961]
DEFB133	-4,80892	2,08E-04	Homo sapiens defensin, beta 133 (DEFB133), mRNA [NM_001166478]
BOD1L	-1,75164	2,08E-04	Homo sapiens biorientation of chromosomes in cell division 1-like (BOD1L), mRNA [NM_148894]
DAGLA	-4,60519	2,09E-04	Homo sapiens diacylglycerol lipase, alpha (DAGLA), mRNA [NM_006133]
HOXD12	-4,64403	2,10E-04	Homo sapiens homeobox D12 (HOXD12), mRNA [NM_021193]

TRDN	-4,62099	2,11E-04	Homo sapiens triadin (TRDN), transcript variant 2, mRNA [NM_001251987]
KCND3	-4,02663	2,11E-04	Homo sapiens potassium voltage-gated channel, Shal-related subfamily, member 3 (KCND3), mRNA [NM_004980]
LOC645434	-3,48211	2,13E-04	Homo sapiens uncharacterized LOC645434 (LOC645434), non-coding RNA [NR_033919]
MTTP	-4,98791	2,13E-04	Homo sapiens microsomal triglyceride transfer protein (MTTP), mRNA [NM_000253]
FAM164A	-4,69825	2,14E-04	Homo sapiens family with sequence similarity 164, member A (FAM164A), mRNA [NM_016010]
GOLGA8E	-4,04358	2,14E-04	Homo sapiens golgin A8 family, member E (GOLGA8E), non-coding RNA [NR_033350]
LOC389043	-5,09899	2,14E-04	Homo sapiens uncharacterized LOC389043 (LOC389043), non-coding RNA [NR_036499]
RPTN	-4,66196	2,15E-04	Homo sapiens repetin (RPTN), mRNA [NM_001122965]
LOC100509860	-4,358	2,15E-04	PREDICTED: Homo sapiens hypothetical protein LOC100509860 (LOC100509860), mRNA [XM_003119816]
DKFZP586I1420	1,217739	2,17E-04	Homo sapiens uncharacterized protein DKFZp586I1420 (DKFZP586I1420), non-coding RNA [NR_002186]
FBXO22	-1,32892	2,17E-04	Homo sapiens F-box protein 22 (FBXO22), transcript variant 1, mRNA [NM_147188]
TRIML1	-4,60988	2,17E-04	Homo sapiens tripartite motif family-like 1 (TRIML1), mRNA [NM_178556]
FLJ41350	-4,61088	2,18E-04	Homo sapiens uncharacterized LOC399806 (FLJ41350), non-coding RNA [NR_029380]
FLJ43390	-4,07779	2,19E-04	Homo sapiens uncharacterized LOC646113 (FLJ43390), non-coding RNA [NR_015358]
SREBF1	-1,21631	2,21E-04	Homo sapiens sterol regulatory element binding transcription factor 1 (SREBF1), mRNA [NM_001005291]
SLC47A1	-4,98919	2,22E-04	Homo sapiens solute carrier family 47, member 1 (SLC47A1), mRNA [NM_018242]
ATP6V0C	-1,6609	2,24E-04	Homo sapiens ATPase, H+ transporting, lysosomal 16kDa, V0 subunit c (ATP6V0C), , mRNA [NM_001694]
SLC9A10	-4,63602	2,25E-04	Homo sapiens solute carrier family 9, member 10 (SLC9A10), mRNA [NM_183061]
BIN2	-4,62134	2,26E-04	Homo sapiens bridging integrator 2 (BIN2), mRNA [NM_016293]
C14orf48	-4,59534	2,27E-04	Homo sapiens chromosome 14 open reading frame 48 (C14orf48), transcript variant 3, non-coding RNA [NR_024184]
DMPK	1,28195	2,28E-04	Homo sapiens dystrophia myotonica-protein kinase (DMPK), transcript variant 2, mRNA [NM_004409]
LOC407835	1,211251	2,28E-04	Homo sapiens mitogen-activated protein kinase kinase 2 pseudogene (LOC407835), non-coding RNA [NR_002144]
CRISP1	-4,91498	2,29E-04	Homo sapiens cysteine-rich secretory protein 1 (CRISP1), transcript variant 1, mRNA [NM_001131]
ENKUR	-4,67849	2,29E-04	Homo sapiens enkurin, TRPC channel interacting protein (ENKUR), mRNA [NM_145010]
CASC3	1,157559	2,30E-04	Homo sapiens cancer susceptibility candidate 3 (CASC3), mRNA [NM_007359]
TAS2R38	-4,67235	2,30E-04	Homo sapiens taste receptor, type 2, member 38 (TAS2R38), mRNA [NM_176817]
AQP6	-4,65043	2,31E-04	Homo sapiens aquaporin 6, kidney specific (AQP6), mRNA [NM_001652]
CLTA	1,2425	2,31E-04	Homo sapiens clathrin, light chain A (CLTA), transcript variant 2, mRNA [NM_007096]
CHRDL2	-4,63546	2,31E-04	Homo sapiens chordin-like 2 (CHRDL2), mRNA [NM_015424]
LOC100129603	-4,70331	2,31E-04	Homo sapiens hypothetical protein LOC389457, mRNA (cDNA clone IMAGE:5267367), [BC038729]

CLN8	-4,70387	2,32E-04	Homo sapiens ceroid-lipofuscinosis, neuronal 8 (epilepsy, progressive with mental retardation), mRNA [NM_018941]
SLC5A8	-5,12689	2,32E-04	Homo sapiens solute carrier family 5 (iodide transporter), member 8 (SLC5A8), mRNA [NM_145913]
CCT3	-4,55727	2,32E-04	chaperonin containing TCP1, subunit 3 (gamma) [Source:HGNC Symbol;Acc:1616] [ENST00000368256]
LOC100132352	-4,1036	2,32E-04	Homo sapiens FSHD region gene 1 pseudogene (LOC100132352), non-coding RNA [NR_034006]
SERPINH1	-1,71773	2,32E-04	Homo sapiens serpin peptidase inhibitor, clade H (heat shock protein 47), member 1, mRNA [NM_001207014]
CAGE1	-4,68339	2,33E-04	Homo sapiens cancer antigen 1 (CAGE1), transcript variant 2, mRNA [NM_001170693]
IRF8	-4,60824	2,33E-04	Homo sapiens interferon regulatory factor 8 (IRF8), mRNA [NM_002163]
PRDM14	-4,44108	2,33E-04	Homo sapiens PR domain containing 14 (PRDM14), mRNA [NM_024504]
WDR17	-4,65812	2,34E-04	Homo sapiens WD repeat domain 17 (WDR17), transcript variant 1, mRNA [NM_170710]
C7orf31	-4,19641	2,34E-04	Homo sapiens chromosome 7 open reading frame 31 (C7orf31), mRNA [NM_138811]
OR13C2	-4,60412	2,34E-04	Homo sapiens olfactory receptor, family 13, subfamily C, member 2 (OR13C2), mRNA [NM_001004481]
EBLN1	-4,60576	2,35E-04	Homo sapiens endogenous Bornavirus-like nucleoprotein 1 (EBLN1), mRNA [NM_001199938]
NOM1	-4,62421	2,35E-04	Homo sapiens cDNA FLJ16401 fis, clone UTERU2004299, [AK131362]
REEP6	1,131823	2,36E-04	Homo sapiens receptor accessory protein 6 (REEP6), mRNA [NM_138393]
MAP4K2	-1,18192	2,36E-04	Homo sapiens mitogen-activated protein kinase kinase kinase kinase 2 (MAP4K2), mRNA [NM_004579]
NETO1	-4,7459	2,36E-04	Homo sapiens neuropilin (NRP) and tolloid (TLL)-like 1 (NETO1), transcript variant 1, mRNA [NM_138999]
FAM160A1	-4,60422	2,36E-04	Homo sapiens family with sequence similarity 160, member A1 (FAM160A1), mRNA [NM_001109977]
RPPH1	-4,73899	2,36E-04	Homo sapiens fmRA sequence [CA413366]
TNFRSF13B	-5,46835	2,36E-04	Homo sapiens tumor necrosis factor receptor superfamily, member 13B (TNFRSF13B), mRNA [NM_012452]
ABHD12B	-4,62339	2,37E-04	Homo sapiens abhydrolase domain containing 12B (ABHD12B), transcript variant 1, mRNA [NM_001206673]
KCNK18	-4,27956	2,37E-04	Homo sapiens potassium channel, subfamily K, member 18 (KCNK18), mRNA [NM_181840]
EFHB	-4,60427	2,38E-04	Homo sapiens cDNA clone IMAGE:5295205, with apparent retained intron, [BC043212]
ZNF695	-4,57361	2,38E-04	Homo sapiens zinc finger protein 695 (ZNF695), transcript variant 1, mRNA [NM_020394]
SRP14	-1,66311	2,39E-04	Homo sapiens signal recognition particle 14kDa (homologous Alu RNA binding protein), mRNA [NM_003134]
FAM69C	-4,0897	2,39E-04	Homo sapiens family with sequence similarity 69, member C (FAM69C), mRNA [NM_001044369]
SYNDIG1	-1,66081	2,39E-04	Homo sapiens synapse differentiation inducing 1 (SYNDIG1), mRNA [NM_024893]
NAALAD2	-4,589	2,40E-04	Homo sapiens N-acetylated alpha-linked acidic dipeptidase 2 (NAALAD2), mRNA [NM_005467]
LOC401442	-4,62391	2,40E-04	PREDICTED: Homo sapiens hypothetical LOC401442 (LOC401442), miscRNA [XR_110089]
STK17B	-4,55422	2,40E-04	Homo sapiens serine/threonine kinase 17b (apoptosis-inducing), mRNA, partial cds, [BC052561]
TLE1	-4,54709	2,42E-04	transducin-like enhancer of split 1 (E(sp1) homolog, Drosophila) [Source:HGNC Symbol;Acc:11837] [ENST00000376484]

MLANA	-4,59851	2,42E-04	Homo sapiens melan-A (MLANA), mRNA [NM_005511]
ARMC4	-5,07433	2,43E-04	armadillo repeat containing 4 [Source:HGNC Symbol;Acc:25583] [ENST00000239715]
FGFR2	-4,25619	2,44E-04	Homo sapiens fibroblast growth factor receptor 2 (FGFR2), transcript variant 2, mRNA [NM_022970]
RPS13	-1,61759	2,44E-04	Homo sapiens ribosomal protein S13 (RPS13), mRNA [NM_001017]
PLEKHA7	-3,6765	2,45E-04	Homo sapiens pleckstrin homology domain containing, family A member 7 (PLEKHA7), mRNA [NM_175058]
ABCA17P	-4,67714	2,46E-04	Homo sapiens ATP-binding cassette, sub-family A (ABC1), member 17, pseudogene (ABCA17P),[NR_003574]
OR4C12	-3,9705	2,46E-04	Homo sapiens olfactory receptor, family 4, subfamily C, member 12 (OR4C12), mRNA [NM_001005270]
CD300LD	-5,05253	2,47E-04	Homo sapiens CD300 molecule-like family member d (CD300LD), mRNA [NM_001115152]
TPD52L3	-5,08637	2,47E-04	Homo sapiens tumor protein D52-like 3 (TPD52L3), transcript variant 1, mRNA [NM_033516]
MATN3	-4,24807	2,47E-04	Homo sapiens matrilin 3 (MATN3), mRNA [NM_002381]
LOC100506127	-4,6188	2,49E-04	Homo sapiens hypothetical LOC387790, mRNA (cDNA clone IMAGE:4811654), [BC040665]
LOC100506272	-4,7196	2,49E-04	PREDICTED: Homo sapiens hypothetical LOC100506272 (LOC100506272), miscRNA [XR_109813]
RUFY3	-4,59683	2,49E-04	Homo sapiens RUN and FYVE domain containing 3 (RUFY3), transcript variant 1, mRNA [NM_001037442]
TGM4	-4,68529	2,50E-04	Homo sapiens transglutaminase 4 (prostate) (TGM4), mRNA [NM_003241]
LOC100129322	-4,61452	2,50E-04	Homo sapiens cDNA FLJ45776 fis, clone NETRP2004090, [AK127678]
BANF1	1,368748	2,50E-04	Homo sapiens barrier to autointegration factor 1 (BANF1), transcript variant 1, mRNA [NM_003860]
DPY19L4	-4,18557	2,51E-04	Homo sapiens dpy-19-like 4 (C, elegans) (DPY19L4), mRNA [NM_181787]
LINC00488	-4,00927	2,51E-04	Homo sapiens long intergenic non-protein coding RNA 488 (LINC00488), non-coding RNA [NR_026767]
SOX2-OT	-4,55903	2,54E-04	Homo sapiens SOX2 overlapping transcript (non-protein coding) (SOX2-OT), non-coding RNA [NR_004053]
FAM194B	-4,58846	2,54E-04	Homo sapiens family with sequence similarity 194, member B (FAM194B), mRNA [NM_182542]
MGMT	1,279605	2,54E-04	Homo sapiens O-6-methylguanine-DNA methyltransferase (MGMT), mRNA [NM_002412]
GLDN	1,474072	2,55E-04	Homo sapiens gliomedin (GLDN), mRNA [NM_181789]
AP2S1	-1,6943	2,55E-04	Homo sapiens adaptor-related protein complex 2, sigma 1 subunit (AP2S1), mRNA [NM_004069]
C21orf128	-4,59329	2,55E-04	Homo sapiens chromosome 21 open reading frame 128 (C21orf128), non-coding RNA [NR_027243]
SLC3A1	-4,63968	2,55E-04	Homo sapiens SLC3A1 variant F (SLC3A1) mRNA, complete cds, alternatively spliced, [DQ023516]
MYO5B	-5,04487	2,56E-04	Homo sapiens myosin VB (MYO5B), mRNA [NM_001080467]
KCNQ5	-4,64831	2,57E-04	potassium voltage-gated channel, KQT-like subfamily, member 5 [Source:HGNC Symbol;Acc:6299] [ENST00000370392]
LOC729047	-4,73067	2,57E-04	Homo sapiens cDNA clone IMAGE:5271023, [BC039368]
LOC642852	-1,24494	2,57E-04	Homo sapiens uncharacterized LOC642852 (LOC642852), non-coding RNA [NR_026943]
TBPL2	-5,09192	2,57E-04	Homo sapiens TATA box binding protein like 2 (TBPL2), mRNA [NM_199047]
MANSC4	-4,60927	2,58E-04	Homo sapiens MANSC domain containing 4 (MANSC4), mRNA [NM_001146221]

LOC644961	-1,75678	2,58E-04	Homo sapiens mRNA; cDNA DKFZp686I06131 (from clone DKFZp686I06131), [BX648289]
GSTP1	-1,57672	2,59E-04	Homo sapiens glutathione S-transferase pi 1 (GSTP1), mRNA [NM_000852]
GAL3ST3	-4,63405	2,59E-04	Homo sapiens galactose-3-O-sulfotransferase 3 (GAL3ST3), mRNA [NM_033036]
LINC00304	-5,0408	2,59E-04	Homo sapiens long intergenic non-protein coding RNA 304 (LINC00304), non-coding RNA [NR_024347]
SLC11A1	-5,04348	2,60E-04	Homo sapiens solute carrier family 11 , member 1 [Source:HGNC Symbol;Acc:10907] [ENST00000469799]
HBB	-4,58603	2,61E-04	hemoglobin, beta [Source:HGNC Symbol;Acc:4827] [ENST00000380315]
OR5AR1	-4,69387	2,62E-04	Homo sapiens olfactory receptor, family 5, subfamily AR, member 1 (OR5AR1), mRNA [NM_001004730]
FBXO11	-4,63733	2,63E-04	Homo sapiens F-box protein 11 [Source:HGNC Symbol;Acc:13590] [ENST00000434234]
BCL11A	-3,96342	2,63E-04	Homo sapiens B-cell CLL/lymphoma 11A (zinc finger protein) (BCL11A), transcript variant 1, mRNA [NM_022893]
GM140	-4,81131	2,63E-04	Homo sapiens gm140 mRNA, partial cds, [AF387616]
WSB1	-1,2154	2,64E-04	Homo sapiens WD repeat and SOCS box containing 1 (WSB1), transcript variant 1, mRNA [NM_015626]
LOC387895	-4,66421	2,64E-04	Homo sapiens cDNA clone IMAGE:5267655, [BC038732]
LOC283174	-4,69619	2,66E-04	Homo sapiens uncharacterized LOC283174 (LOC283174), non-coding RNA [NR_024344]
LOC285205	-4,81187	2,66E-04	Homo sapiens uncharacterized LOC285205 (LOC285205), non-coding RNA [NR_015394]
KIAA0319	-3,98723	2,66E-04	Homo sapiens KIAA0319 (KIAA0319), transcript variant 1, mRNA [NM_014809]
IFNA8	-4,60676	2,67E-04	Homo sapiens interferon, alpha 8 (IFNA8), mRNA [NM_002170]
APAF1	-1,48419	2,67E-04	Homo sapiens apoptotic peptidase activating factor 1 (APAF1), transcript variant 3, mRNA [NM_181861]
TMEM128	1,108372	2,67E-04	Homo sapiens transmembrane protein 128 (TMEM128), mRNA [NM_032927]
XCL1	-5,03941	2,68E-04	Homo sapiens chemokine (C motif) ligand 1 (XCL1), mRNA [NM_002995]
CCS	1,253966	2,68E-04	Homo sapiens copper chaperone for superoxide dismutase (CCS), mRNA [NM_005125]
CLEC2D	-4,65026	2,68E-04	Homo sapiens C-type lectin domain family 2, member D (CLEC2D), transcript variant 2, mRNA [NM_001004419]
ZNF208	-4,14662	2,68E-04	Homo sapiens zinc finger protein 208 (ZNF208), mRNA [NM_007153]
LOC650293	-4,57819	2,68E-04	Homo sapiens seven transmembrane helix receptor (LOC650293), mRNA [NM_001040071]
MED27	-5,20246	2,68E-04	Homo sapiens mediator complex subunit 27 [Source:HGNC Symbol;Acc:2377] [ENST00000474263]
ARVCF	-4,75776	2,69E-04	Homo sapiens armadillo repeat gene deleted in velocardiofacial syndrome (ARVCF), mRNA [NM_001670]
TP63	-4,1093	2,70E-04	Homo sapiens tumor protein p63 (TP63), transcript variant 1, mRNA [NM_003722]
IL31	-5,02929	2,70E-04	Homo sapiens interleukin 31 (IL31), mRNA [NM_001014336]
PCDH9	-5,04921	2,71E-04	Homo sapiens protocadherin 9 (PCDH9), transcript variant 1, mRNA [NM_203487]
REG4	-3,96618	2,71E-04	Homo sapiens regenerating islet-derived family, member 4 (REG4), transcript variant 2, mRNA [NM_032044]
AMACR	1,139237	2,72E-04	Homo sapiens alpha-methylacyl-CoA racemase, mRNA [NM_001167595]
UBE2QL1	-4,62308	2,73E-04	Homo sapiens ubiquitin-conjugating enzyme E2Q family-like 1 (UBE2QL1), mRNA [NM_001145161]

JAKMIP3	-5,02264	2,75E-04	Homo sapiens Janus kinase and microtubule interacting protein 3 (JAKMIP3), mRNA [NM_001105521]
FAM55A	-4,64633	2,75E-04	Homo sapiens family with sequence similarity 55, member A (FAM55A), mRNA [NM_152315]
IL4	-3,78397	2,76E-04	Homo sapiens interleukin 4 (IL4), transcript variant 1, mRNA [NM_000589]
LOC645984	-4,04924	2,76E-04	Homo sapiens cDNA FLJ38117 fis, clone D3OST2003797, [AK095436]
FLJ13744	-3,90999	2,77E-04	Homo sapiens cDNA FLJ13744 fis, clone PLACE3000230, [AK023806]
FLJ33581	-5,14133	2,77E-04	Homo sapiens uncharacterized LOC400839 (FLJ33581), non-coding RNA [NR_040102]
OR6C70	-4,61926	2,78E-04	Homo sapiens olfactory receptor, family 6, subfamily C, member 70 (OR6C70), mRNA [NM_001005499]
LOC284889	1,22031	2,78E-04	Homo sapiens uncharacterized LOC284889 (LOC284889), non-coding RNA [NR_038911]
FSCB	-4,68593	2,78E-04	Homo sapiens fibrous sheath CABYR binding protein (FSCB), mRNA [NM_032135]
NCOR1	-4,64744	2,78E-04	Homo sapiens nuclear receptor corepressor 1 (NCOR1), transcript variant 2, mRNA [NM_001190438]
NKX2-2	-4,68899	2,79E-04	Homo sapiens NK2 homeobox 2 (NKX2-2), mRNA [NM_002509]
KCNA2	-4,60463	2,80E-04	Homo sapiens potassium voltage-gated channel, shaker-related subfamily, member 2 (KCNA2), mRNA [NM_004974]
KLRD1	-4,68844	2,81E-04	Homo sapiens killer cell lectin-like receptor subfamily D, member 1 (KLRD1), mRNA [NM_002262]
OR2L13	-4,64049	2,81E-04	Homo sapiens olfactory receptor, family 2, subfamily L, member 13 (OR2L13), mRNA [NM_175911]
OR5J2	-4,73923	2,81E-04	Homo sapiens olfactory receptor, family 5, subfamily J, member 2 (OR5J2), mRNA [NM_001005492]
C7orf57	-4,3681	2,81E-04	Homo sapiens chromosome 7 open reading frame 57 (C7orf57), mRNA [NM_001100159]
RAG2	-3,84788	2,82E-04	Homo sapiens recombination activating gene 2 (RAG2), transcript variant 1, mRNA [NM_000536]
OR7C1	-4,7607	2,82E-04	Homo sapiens olfactory receptor, family 7, subfamily C, member 1 (OR7C1), mRNA [NM_198944]
CACNA1H	-1,6817	2,82E-04	Homo sapiens calcium channel, voltage-dependent, T type, alpha 1H subunit (CACNA1H), mRNA [NM_021098]
EFCAB11	-5,04107	2,82E-04	EF-hand calcium binding domain 11 [Source:HGNC Symbol;Acc:20357] [ENST00000555872]
CLEC2A	-4,7894	2,83E-04	Homo sapiens C-type lectin domain family 2, member A (CLEC2A), mRNA [NM_001130711]
DKFZp686D0853	-4,69879	2,84E-04	Homo sapiens uncharacterized LOC401613 (DKFZp686D0853), non-coding RNA [NR_033974]
CD93	-3,93445	2,84E-04	Homo sapiens CD93 molecule (CD93), mRNA [NM_012072]
TTTY4C	-5,00662	2,84E-04	Homo sapiens testis-specific transcript, Y-linked 4C (non-protein coding) (TTTY4C), non-coding RNA [NR_002177]
DEFB113	-5,02531	2,84E-04	Homo sapiens defensin, beta 113 (DEFB113), mRNA [NM_001037729]
RPL23A	-1,73149	2,84E-04	Homo sapiens ribosomal protein L23a (RPL23A), mRNA [NM_000984]
BRCC3	-5,03304	2,85E-04	BRCA1/BRCA2-containing complex, subunit 3 [Source:HGNC Symbol;Acc:24185] [ENST00000399026]
ACTL8	-2,38942	2,85E-04	Homo sapiens actin-like 8 (ACTL8), mRNA [NM_030812]
SEPT3	-4,59878	2,85E-04	Homo sapiens septin 3 (SEPT3), transcript variant A, mRNA [NM_145733]
LOC647323	-5,12347	2,85E-04	Homo sapiens uncharacterized LOC647323 (LOC647323), non-coding RNA [NR_033944]

LOC284100	-5,0025	2,85E-04	Homo sapiens tyrosine 3-monooxygenase/tryptophan 5-monooxygenase activation protein, [NR_024178]
ADH7	-4,76665	2,85E-04	Homo sapiens alcohol dehydrogenase 7 (class IV), mu or sigma polypeptide (ADH7), mRNA [NM_000673]
CCDC68	-4,74434	2,87E-04	Homo sapiens coiled-coil domain containing 68 (CCDC68), transcript variant 1, mRNA [NM_025214]
ARMC3	-4,5929	2,88E-04	Homo sapiens armadillo repeat containing 3 (ARMC3), mRNA [NM_173081]
TMEM119	1,25679	2,89E-04	Homo sapiens transmembrane protein 119 (TMEM119), mRNA [NM_181724]
ODF2L	-5,02589	2,89E-04	Homo sapiens outer dense fiber of sperm tails 2-like (ODF2L), transcript variant 3, mRNA [NM_001184765]
SULT1B1	-4,28173	2,89E-04	Homo sapiens sulfotransferase family, cytosolic, 1B, member 1 (SULT1B1), mRNA [NM_014465]
GRIK3	-4,72493	2,90E-04	Homo sapiens glutamate receptor, ionotropic, kainate 3 (GRIK3), mRNA [NM_000831]
LILRB4	-4,65235	2,91E-04	Homo sapiens leukocyte immunoglobulin-like receptor, subfamily B , mRNA [NM_006847]
LOC100128979	-4,57519	2,91E-04	Homo sapiens hypothetical LOC100128979 (LOC100128979), miscRNA [XR_109205]
PPP1R2P9	-4,62407	2,92E-04	Homo sapiens protein phosphatase 1, regulatory (inhibitor) subunit 2 pseudogene 9 (PPP1R2P9), [NR_002191]
BAAT	-4,6908	2,92E-04	Homo sapiens bile acid CoA: amino acid N-acyltransferase (glycine N-choloyltransferase), mRNA [NM_001701]
SLC22A2	-4,99481	2,92E-04	Homo sapiens solute carrier family 22 , [Source:HGNC Symbol;Acc:10966] [ENST00000366952]
RPL37A	-1,66176	2,92E-04	Homo sapiens ribosomal protein L37a (RPL37A), mRNA [NM_000998]
BTBD8	-4,74982	2,92E-04	Homo sapiens BTB (POZ) domain containing 8 (BTBD8), mRNA [NM_183242]
MARK2	-1,18993	2,93E-04	Homo sapiens MAP/microtubule affinity-regulating kinase 2 (MARK2), transcript variant 4, mRNA [NM_001039469]
WIPF3	-4,65702	2,94E-04	WAS/WASL interacting protein family, member 3 [Source:HGNC Symbol;Acc:22004] [ENST00000409123]
CT62	-4,47902	2,94E-04	Homo sapiens cancer/testis antigen 62 (CT62), mRNA [NM_001102658]
LOC643723	-4,63056	2,95E-04	Homo sapiens uncharacterized LOC643723 (LOC643723), non-coding RNA [NR_038845]
ASB4	-4,96707	2,96E-04	Homo sapiens ankyrin repeat and SOCS box containing 4 (ASB4), transcript variant 1, mRNA [NM_016116]
FAM13C	-4,6672	2,97E-04	Homo sapiens family with sequence similarity 13, member C (FAM13C), transcript variant 2, mRNA [NM_001001971]
GUCY2F	-4,11854	2,97E-04	Homo sapiens guanylate cyclase 2F, retinal (GUCY2F), mRNA [NM_001522]
RCE1	1,197074	2,98E-04	Homo sapiens RCE1 homolog, prenyl protein peptidase (S, cerevisiae) (RCE1), mRNA [NM_005133]
ANKRD44	-5,01051	2,98E-04	ankyrin repeat domain 44 [Source:HGNC Symbol;Acc:25259] [ENST00000328737]
INE2	-4,66101	2,98E-04	Homo sapiens inactivation escape 2 (non-protein coding) (INE2), non-coding RNA [NR_002725]
PRB4	-4,71263	2,99E-04	Homo sapiens proline-rich protein BstNI subfamily 4 (PRB4), mRNA [NM_002723]
SERPINB6	1,24651	2,99E-04	Homo sapiens serpin peptidase inhibitor, clade B (ovalbumin), member 6 (SERPINB6), , mRNA [NM_001195291]
HHLA2	-4,6705	3,00E-04	Homo sapiens HERV-H LTR-associating 2 (HHLA2), mRNA [NM_007072]
CLEC5A	-5,00359	3,00E-04	Homo sapiens C-type lectin domain family 5, member A (CLEC5A), mRNA [NM_013252]
RAD21L1	-4,69826	3,01E-04	Homo sapiens RAD21-like 1 (S, pombe) (RAD21L1), mRNA [NM_001136566]

OR52E2	-4,77647	3,01E-04	Homo sapiens olfactory receptor, family 52, subfamily E, member 2 (OR52E2), mRNA [NM_001005164]
ST6GAL2	-4,68037	3,01E-04	Homo sapiens ST6 beta-galactosamide alpha-2,6-sialyltranferase 2 (ST6GAL2), mRNA [NM_032528]
PRSS58	-4,09379	3,01E-04	Homo sapiens protease, serine, 58 (PRSS58), mRNA [NM_001001317]
LOC285629	-4,71543	3,02E-04	DB296219 BNGH42 Homo sapiens cDNA clone BNGH42003641 3', mRNA sequence [DB296219]
SLED1	-4,58859	3,02E-04	Homo sapiens proteoglycan 3 pseudogene (SLED1), non-coding RNA [NR_003542]
ELF5	-4,00271	3,03E-04	Homo sapiens E74-like factor 5 (ets domain transcription factor) (ELF5), transcript variant 1, mRNA [NM_198381]
PROX1	-4,70131	3,04E-04	Homo sapiens prospero homeobox 1 (PROX1), mRNA [NM_002763]
TMED11P	-4,68055	3,04E-04	Homo sapiens transmembrane emp24 protein transport domain containing 11, pseudogene, [NR_033768]
CYP4F3	-4,18066	3,05E-04	Homo sapiens cytochrome P450, family 4, subfamily F, polypeptide 3 (CYP4F3), , mRNA [NM_000896]
LOC285758	-4,73786	3,06E-04	Homo sapiens uncharacterized LOC285758 (LOC285758), non-coding RNA [NR_038863]
DDX4	-4,67912	3,06E-04	Homo sapiens DEAD (Asp-Glu-Ala-Asp) box polypeptide 4 (DDX4), transcript variant 1, mRNA [NM_024415]
KCNC2	-4,69567	3,07E-04	Homo sapiens potassium voltage-gated channel, Shaw-related subfamily, member 2 (KCNC2), mRNA [NM_139137]
C19orf75	-4,02378	3,08E-04	Homo sapiens chromosome 19 open reading frame 75 (C19orf75), mRNA [NM_173635]
PRH2	-4,81222	3,08E-04	Homo sapiens proline-rich protein HaeIII subfamily 2 (PRH2), transcript variant 1, mRNA [NM_005042]
HERC2P4	1,373399	3,08E-04	Homo sapiens hect domain and RLD 2 pseudogene 4 (HERC2P4), non-coding RNA [NR_002827]
CLEC12A	-4,86071	3,10E-04	Homo sapiens C-type lectin domain family 12, member A (CLEC12A), transcript variant 1, mRNA [NM_138337]
POM121L10P	-4,6255	3,11E-04	Homo sapiens POM121 membrane glycoprotein-like 10, pseudogene (POM121L10P), non-coding RNA [NR_024593]
SUCNR1	-4,99122	3,12E-04	Homo sapiens succinate receptor 1 (SUCNR1), mRNA [NM_033050]
ECHDC1	-4,99165	3,12E-04	enoyl CoA hydratase domain containing 1 [Source:HGNC Symbol;Acc:21489] [ENST00000368287]
GPR1	-5,05119	3,12E-04	Homo sapiens G protein-coupled receptor 1 (GPR1), transcript variant 1, mRNA [NM_005279]
LOC154872	-4,5198	3,13E-04	Homo sapiens uncharacterized LOC154872 (LOC154872), mRNA [NM_001024603]
LOC442132	-4,9627	3,13E-04	Homo sapiens golgin A6 family-like 1 pseudogene (LOC442132), non-coding RNA [NR_033906]
ITGB2	-4,28657	3,14E-04	Homo sapiens integrin, beta 2 (complement component 3 receptor 3 and 4 subunit) (ITGB2), mRNA [NM_000211]
WHAMMP3	-5,00343	3,15E-04	Homo sapiens WAS protein homolog ass. with actin, golgi membranes , microtubules pseudogene 3, [NR_003521]
STIL	-1,4205	3,15E-04	Homo sapiens SCL/TAL1 interrupting locus (STIL), transcript variant 1, mRNA [NM_001048166]
HSP90B1	-1,61404	3,16E-04	Homo sapiens heat shock protein 90kDa beta (Grp94), member 1 (HSP90B1), mRNA [NM_003299]
FGG	-4,78696	3,16E-04	Homo sapiens fibrinogen gamma chain (FGG), transcript variant gamma-A, mRNA [NM_000509]
PDZD9	-4,62672	3,16E-04	Homo sapiens PDZ domain containing 9 (PDZD9), transcript variant 1, mRNA [NM_173806]
GRM6	-4,69495	3,18E-04	Homo sapiens glutamate receptor, metabotropic 6 (GRM6), mRNA [NM_000843]
CLEC7A	-5,01056	3,18E-04	Homo sapiens C-type lectin domain family 7, member A (CLEC7A), transcript variant 1, mRNA [NM_197947]

C6orf146	-4,45573	3,19E-04	Homo sapiens chromosome 6 open reading frame 146 (C6orf146), mRNA [NM_173563]
TMEM154	-5,11443	3,19E-04	Homo sapiens transmembrane protein 154 (TMEM154), mRNA [NM_152680]
SPAG11B	-3,85118	3,21E-04	Homo sapiens sperm associated antigen 11B (SPAG11B), transcript variant A, mRNA [NM_016512]
SLC17A4	-4,56383	3,21E-04	Homo sapiens solute carrier family 17 (sodium phosphate), member 4 (SLC17A4), mRNA [NM_005495]
DLX6	-4,63947	3,22E-04	Homo sapiens distal-less homeobox 6 (DLX6), mRNA [NM_005222]
DEFB125	-3,81623	3,23E-04	Homo sapiens defensin, beta 125 (DEFB125), mRNA [NM_153325]
ABCA12	1,54381	3,24E-04	Homo sapiens ATP-binding cassette, sub-family A (ABC1), member 12 (ABCA12), mRNA [NM_173076]
SKCG-1	-4,81918	3,24E-04	PREDICTED: Homo sapiens sporadic kidney cancer gene 1 (SKCG-1), miscRNA [XR_110517]
C10orf136	-4,55298	3,24E-04	Homo sapiens chromosome 10 open reading frame 136 (C10orf136), non-coding RNA [NR_033923]
TARP	-4,80347	3,24E-04	Homo sapiens TCR gamma alternate reading frame protein (TARP), mRNA [NM_001003799]
TARS2	-4,59974	3,25E-04	threonyl-tRNA synthetase 2, mitochondrial (putative) [Source:HGNC Symbol;Acc:30740] [ENST00000369053]
LYPLA2	-1,27353	3,25E-04	Homo sapiens lysophospholipase II (LYPLA2), mRNA [NM_007260]
CNTN1	-4,27705	3,25E-04	Homo sapiens contactin 1 (CNTN1), transcript variant 1, mRNA [NM_001843]
HRNR	-4,95318	3,25E-04	Homo sapiens hornerin (HRNR), mRNA [NM_001009931]
TMEM147	1,436085	3,26E-04	Homo sapiens transmembrane protein 147 (TMEM147), transcript variant 1, mRNA [NM_032635]
DAOA	-4,94174	3,26E-04	Homo sapiens D-amino acid oxidase activator (DAOA), transcript variant 1, mRNA [NM_172370]
SEPT7P2	-4,63724	3,26E-04	Homo sapiens septin 7 pseudogene 2 (SEPT7P2), non-coding RNA [NR_024271]
ODF1	-4,93071	3,27E-04	Homo sapiens outer dense fiber of sperm tails 1 (ODF1), mRNA [NM_024410]
NUP210P1	-4,06166	3,27E-04	Homo sapiens nucleoporin 210kDa pseudogene 1 (NUP210P1), non-coding RNA [NR_034158]
GCNT2	-4,6144	3,28E-04	Homo sapiens glucosaminyl (N-acetyl) transferase 2, I-branching enzyme (I blood group), mRNA [NM_001491]
DPY19L2P1	-4,96164	3,29E-04	Homo sapiens dpy-19-like 2 pseudogene 1 (C, elegans) (DPY19L2P1), non-coding RNA [NR_002833]
EFCAB5	-4,63027	3,29E-04	Homo sapiens EF-hand calcium binding domain 5 (EFCAB5), transcript variant 1, mRNA [NM_198529]
SHISA9	-5,08389	3,29E-04	Homo sapiens shisa homolog 9 (Xenopus laevis) (SHISA9), transcript variant 1, mRNA [NM_001145204]
ZIK1	1,280713	3,31E-04	Homo sapiens zinc finger protein interacting with K protein 1 homolog (mouse) (ZIK1), mRNA [NM_001010879]
OR2H1	-4,9853	3,32E-04	Homo sapiens olfactory receptor, family 2, subfamily H, member 1 (OR2H1), mRNA [NM_030883]
OR5D18	-4,93954	3,32E-04	Homo sapiens olfactory receptor, family 5, subfamily D, member 18 (OR5D18), mRNA [NM_001001952]
GAPDH	-1,74006	3,33E-04	Homo sapiens glyceraldehyde-3-phosphate dehydrogenase (GAPDH), mRNA [NM_002046]
FLJ42875	-4,67625	3,33E-04	Homo sapiens mRNA; cDNA DKFZp761G0122 (from clone DKFZp761G0122), [AL713743]
LOC100129055	-4,96564	3,33E-04	Homo sapiens cyclin Y-like pseudogene (LOC100129055), non-coding RNA [NR_024524]
LOC388630	-4,59742	3,33E-04	Homo sapiens UPF0632 protein A (LOC388630), mRNA [NM_001194986]
QRFPR	-4,63604	3,34E-04	Homo sapiens pyroglutamylated RFamide peptide receptor (QRFPR), mRNA [NM_198179]

TTC32	-4,92802	3,34E-04	tetratricopeptide repeat domain 32 [Source:HGNC Symbol;Acc:32954] [ENST00000402414]
AJAP1	-3,69355	3,34E-04	Homo sapiens adherens junctions associated protein 1 (AJAP1), transcript variant 2, mRNA [NM_001042478]
ACSBG1	-4,70046	3,35E-04	acyl-CoA synthetase bubblegum family member 1 [Source:HGNC Symbol;Acc:29567] [ENST00000258873]
OLAH	-4,55671	3,35E-04	oleoyl-ACP hydrolase [Source:HGNC Symbol;Acc:25625] [ENST00000378225]
TTL	-4,60937	3,35E-04	Homo sapiens tubulin tyrosine ligase (TTL), transcript variant TTL-T, non-coding RNA [NR_024505]
LPL	-4,14783	3,35E-04	Homo sapiens lipoprotein lipase (LPL), mRNA [NM_000237]
IL9	-4,70233	3,36E-04	Homo sapiens interleukin 9 (IL9), mRNA [NM_000590]
RASSF1	-1,62205	3,36E-04	Ras association (RalGDS/AF-6) domain family member 1 [Source:HGNC Symbol;Acc:9882] [ENST00000494145]
MCF2L	-5,15592	3,36E-04	MCF,2 cell line derived transforming sequence-like [Source:HGNC Symbol;Acc:14576] [ENST00000397036]
C21orf54	-4,68231	3,36E-04	Homo sapiens chromosome 21 open reading frame 54 (C21orf54), non-coding RNA [NR_024102]
RPS28	-2,03371	3,37E-04	Homo sapiens ribosomal protein S28 (RPS28), mRNA [NM_001031]
SPANXN3	-4,59842	3,37E-04	Homo sapiens SPANX family, member N3 (SPANXN3), mRNA [NM_001009609]
LOC100128264	-4,87175	3,37E-04	Homo sapiens uncharacterized LOC100128264 (LOC100128264), non-coding RNA [NR_038945]
LOC100124692	-4,66935	3,39E-04	Homo sapiens maltase-glucoamylase (alpha-glucosidase) pseudogene , non-coding RNA [NR_003717]
ZNF569	-5,09657	3,39E-04	Homo sapiens zinc finger protein 569 (ZNF569), mRNA [NM_152484]
OR5H2	-4,93763	3,39E-04	Homo sapiens olfactory receptor, family 5, subfamily H, member 2 (OR5H2), mRNA [NM_001005482]
IQCJ	-4,7076	3,39E-04	Homo sapiens IQ motif containing J (IQCJ), transcript variant 2, mRNA [NM_001042706]
CTNNA2	-4,50318	3,39E-04	Homo sapiens catenin (cadherin-associated protein), alpha 2 (CTNNA2), transcript variant 1, mRNA [NM_004389]
LOC285943	-4,81843	3,40E-04	PREDICTED: Homo sapiens hypothetical LOC285943 (LOC285943), miscRNA [XR_108755]
CCDC34	-4,73235	3,41E-04	Homo sapiens coiled-coil domain containing 34 (CCDC34), transcript variant 2, mRNA [NM_080654]
COL6A3	-1,2053	3,42E-04	Homo sapiens collagen, type VI, alpha 3 (COL6A3), transcript variant 3, mRNA [NM_057165]
A2ML1	-4,02485	3,42E-04	Homo sapiens alpha-2-macroglobulin-like 1 (A2ML1), mRNA [NM_144670]
LOC339535	-4,70915	3,42E-04	Homo sapiens uncharacterized LOC339535 (LOC339535), non-coding RNA [NR_015407]
MYZAP	-1,36785	3,42E-04	Homo sapiens myocardial zonula adherens protein (MYZAP), transcript variant 1, mRNA [NM_001018100]
HSFY2	-4,95303	3,43E-04	Homo sapiens heat shock transcription factor, Y linked 2 (HSFY2), transcript variant 2, mRNA [NM_001001877]
LOC440925	-4,90721	3,44E-04	Homo sapiens uncharacterized LOC440925 (LOC440925), non-coding RNA [NR_027433]
DLEU2L	-4,5611	3,44E-04	Homo sapiens deleted in lymphocytic leukemia 2-like (DLEU2L), non-coding RNA [NR_002771]
FLJ34503	-4,90343	3,44E-04	Homo sapiens uncharacterized FLJ34503 (FLJ34503), non-coding RNA [NR_027060]
TM4SF20	-4,24584	3,45E-04	Homo sapiens transmembrane 4 L six family member 20 (TM4SF20), mRNA [NM_024795]
C1orf185	-4,64561	3,45E-04	Homo sapiens chromosome 1 open reading frame 185 (C1orf185), mRNA [NM_001136508]
C10orf131	-4,79755	3,45E-04	Homo sapiens chromosome 10 open reading frame 131 (C10orf131), mRNA [NM_001130446]

VWC2	-4,91991	3,45E-04	Homo sapiens von Willebrand factor C domain containing 2 (VWC2), mRNA [NM_198570]
LOC100506898	-5,05016	3,46E-04	PREDICTED: Homo sapiens protein mago nashi homolog 2-like (LOC100506898), miscRNA [XR_132625]
TKTL2	-4,02663	3,46E-04	Homo sapiens transketolase-like 2 (TKTL2), mRNA [NM_032136]
PHLDB2	-4,59606	3,46E-04	Homo sapiens pleckstrin homology-like domain, family B, member 2 (PHLDB2), mRNA [NM_001134438]
RPL32	-1,63609	3,46E-04	Homo sapiens ribosomal protein L32 (RPL32), transcript variant 3, mRNA [NM_001007074]
CNTRL	-1,37152	3,47E-04	Homo sapiens centriolin (CNTRL), mRNA [NM_007018]
ODF2L	-4,67775	3,49E-04	Homo sapiens outer dense fiber of sperm tails 2-like (ODF2L), transcript variant 3, mRNA [NM_001184765]
SLC2A6	1,192809	3,51E-04	Homo sapiens solute carrier family 2 (facilitated glucose transporter), member 6 (SLC2A6), mRNA [NM_017585]
MAP3K15	-4,75341	3,51E-04	Homo sapiens mitogen-activated protein kinase kinase kinase 15 (MAP3K15), mRNA [NM_001001671]
ADAMTS20	-4,72337	3,51E-04	Homo sapiens ADAM metallopeptidase with thrombospondin type 1 motif, 20 (ADAMTS20), mRNA [NM_025003]
GABRB3	-4,89315	3,53E-04	Homo sapiens gamma-aminobutyric acid (GABA) A receptor, beta 3 (GABRB3), mRNA [NM_000814]
SLCO6A1	-4,97618	3,53E-04	Homo sapiens solute carrier organic anion transporter family, member 6A1 (SLCO6A1), mRNA [NM_173488]
TGM5	-3,78362	3,55E-04	Homo sapiens transglutaminase 5 (TGM5), transcript variant 1, mRNA [NM_201631]
UBE4B	-4,6783	3,55E-04	ubiquitination factor E4B [Source:HGNC Symbol;Acc:12500] [ENST00000377153]
DEFB114	-5,05012	3,56E-04	Homo sapiens defensin, beta 114 (DEFB114), mRNA [NM_001037499]
SLC14A1	-5,20712	3,57E-04	Homo sapiens solute carrier family 14 (urea transporter), member 1 (Kidd blood group), mRNA [NM_001146037]
TLE4	-4,97492	3,59E-04	Homo sapiens transducin-like enhancer of split 4 (E(sp1) homolog, Drosophila) [Source:HGNC Symbol;Acc:11840]
GTF2I	-4,99403	3,59E-04	general transcription factor IIi [Source:HGNC Symbol;Acc:4659] [ENST00000473333]
MYNN	-1,31733	3,60E-04	Homo sapiens myoneurin (MYNN), transcript variant 3, mRNA [NM_001185119]
SCRT1	-4,92251	3,60E-04	Homo sapiens scratch homolog 1, zinc finger protein (Drosophila) (SCRT1), mRNA [NM_031309]
C9orf84	-4,59717	3,60E-04	Homo sapiens chromosome 9 open reading frame 84 (C9orf84), transcript variant 1, mRNA [NM_173521]
PTH2R	-4,87484	3,61E-04	Homo sapiens parathyroid hormone 2 receptor (PTH2R), mRNA [NM_005048]
UST	1,242446	3,61E-04	Homo sapiens uronyl-2-sulfotransferase (UST), mRNA [NM_005715]
NRG3	-4,87207	3,62E-04	Homo sapiens neuregulin 3 (NRG3), transcript variant 1, mRNA [NM_001010848]
LRRIQ3	-4,87747	3,62E-04	Homo sapiens leucine-rich repeats and IQ motif containing 3 (LRRIQ3), mRNA [NM_001105659]
RAB39	-4,87243	3,62E-04	Homo sapiens RAB39, member RAS oncogene family (RAB39), mRNA [NM_017516]
APOBEC3A	-4,41155	3,63E-04	Homo sapiens apolipoprotein B mRNA editing enzyme, catalytic polypeptide-like 3A, mRNA [NM_145699]
TMEM196	-4,72896	3,63E-04	Homo sapiens transmembrane protein 196 (TMEM196), mRNA [NM_152774]
THAP5	-4,65643	3,64E-04	Homo sapiens THAP domain containing 5 (THAP5), transcript variant 2, mRNA [NM_182529]
ZBTB8B	-4,72038	3,64E-04	Homo sapiens zinc finger and BTB domain containing 8B (ZBTB8B), mRNA [NM_001145720]
EPHA6	-4,11909	3,65E-04	Homo sapiens EPH receptor A6 (EPHA6), transcript variant 1, mRNA [NM_001080448]

LOC100129894	-4,5758	3,65E-04	Homo sapiens cDNA FLJ37054 fis, clone BRACE2014108, [AK094373]
ACSM4	-4,88572	3,65E-04	Homo sapiens acyl-CoA synthetase medium-chain family member 4 (ACSM4), mRNA [NM_001080454]
C3orf15	-4,65823	3,66E-04	Homo sapiens chromosome 3 open reading frame 15 (C3orf15), mRNA [NM_033364]
PCDHB19P	-4,59064	3,66E-04	Homo sapiens protocadherin beta 19 pseudogene (PCDHB19P), non-coding RNA [NR_001282]
PCDHB6	-4,8619	3,67E-04	Homo sapiens protocadherin beta 6 (PCDHB6), mRNA [NM_018939]
RIMS1	-4,69491	3,67E-04	regulating synaptic membrane exocytosis 1 [Source:HGNC Symbol;Acc:17282] [ENST00000370419]
MKRN9P	-4,6711	3,67E-04	Homo sapiens makorin ring finger protein pseudogene 6, mRNA (cDNA clone IMAGE:5278542), [BC067894]
CHD5	-4,95799	3,67E-04	Homo sapiens chromodomain helicase DNA binding protein 5 (CHD5), mRNA [NM_015557]
TAPT1	-4,8738	3,67E-04	transmembrane anterior posterior transformation 1 [Source:HGNC Symbol;Acc:26887] [ENST00000505317]
LOC100131234	-4,74314	3,68E-04	Homo sapiens familial acute myelogenous leukemia related factor mRNA, complete cds, [EF413001]
WWTR1	-4,73437	3,69E-04	WW domain containing transcription regulator 1 [Source:HGNC Symbol;Acc:24042] [ENST00000474080]
OR4F15	-4,54516	3,69E-04	Homo sapiens olfactory receptor, family 4, subfamily F, member 15 (OR4F15), mRNA [NM_001001674]
LOC728716	-4,56277	3,69E-04	Homo sapiens uncharacterized LOC728716 (LOC728716), non-coding RNA [NR_040065]
CXADR	-4,91017	3,70E-04	Homo sapiens coxsackie virus and adenovirus receptor (CXADR), transcript variant 1, mRNA [NM_001338]
SPINK4	-3,88781	3,70E-04	Homo sapiens serine peptidase inhibitor, Kazal type 4 (SPINK4), mRNA [NM_014471]
ZNF229	-4,95083	3,71E-04	Homo sapiens zinc finger protein 229 (ZNF229), mRNA [NM_014518]
IGF2BP1	-1,47482	3,72E-04	Homo sapiens insulin-like growth factor 2 mRNA binding protein 1 (IGF2BP1), mRNA [NM_006546]
C12orf12	-4,20992	3,72E-04	Homo sapiens chromosome 12 open reading frame 12 (C12orf12), mRNA [NM_152638]
PMP2	-4,58514	3,73E-04	Homo sapiens peripheral myelin protein 2 (PMP2), mRNA [NM_002677]
VSNL1	-4,90413	3,74E-04	Homo sapiens visinin-like 1 (VSNL1), mRNA [NM_003385]
PCDHA1	-4,08373	3,74E-04	Homo sapiens protocadherin alpha 1 (PCDHA1), transcript variant 2, mRNA [NM_031410]
MAGEA1	-4,73629	3,74E-04	Homo sapiens melanoma antigen family A, 1 (directs expression of antigen MZ2-E) (MAGEA1), mRNA [NM_004988]
GDAP1	-3,84516	3,74E-04	Homo sapiens ganglioside-induced differentiation-associated protein 1 (GDAP1), mRNA [NM_018972]
LOC100507199	-4,92007	3,75E-04	Homo sapiens hypothetical LOC100507199, transcript variant 1, miscRNA [XR_110934]
GPR37	-4,72482	3,75E-04	Homo sapiens G protein-coupled receptor 37 (endothelin receptor type B-like) (GPR37), mRNA [NM_005302]
JAKMIP1	-3,8732	3,78E-04	Homo sapiens janus kinase and microtubule interacting protein 1 (JAKMIP1), mRNA [NM_144720]
FABP1	-4,8982	3,78E-04	fatty acid binding protein 1, liver [Source:HGNC Symbol;Acc:3555] [ENST00000495375]
OR6C2	-4,73689	3,79E-04	Homo sapiens olfactory receptor, family 6, subfamily C, member 2 (OR6C2), mRNA [NM_054105]
OR5K1	-4,72065	3,79E-04	Homo sapiens olfactory receptor, family 5, subfamily K, member 1 (OR5K1), mRNA [NM_001004736]
SNHG8	-4,62027	3,80E-04	AGENCOURT_6644733 NIH_MGC_122 Homo sapiens cDNA clone IMAGE:5766924 5', mRNA sequence [BM926530]

RGS6	-4,75301	3,80E-04	Homo sapiens regulator of G-protein signaling 6 (RGS6), transcript variant 4, mRNA [NM_001204418]
C7orf25	1,231901	3,80E-04	Homo sapiens chromosome 7 open reading frame 25 (C7orf25), transcript variant 2, mRNA [NM_024054]
CLEC7A	-4,54758	3,80E-04	Homo sapiens C-type lectin domain family 7, member A (CLEC7A), transcript variant 6, mRNA [NM_197954]
LOC400620	-3,7963	3,80E-04	Homo sapiens, clone IMAGE:3342755, mRNA, partial cds, [BC014643]
ZNF883	-5,08655	3,81E-04	Homo sapiens zinc finger protein 883 (ZNF883), mRNA [NM_001101338]
BCAS1	-3,86846	3,81E-04	Homo sapiens breast carcinoma amplified sequence 1 (BCAS1), mRNA [NM_003657]
ABCB5	-4,0294	3,81E-04	Homo sapiens ATP-binding cassette, sub-family B (MDR/TAP), member 5 (ABCB5), mRNA [NM_001163993]
LOC339926	-4,83442	3,81E-04	Homo sapiens uncharacterized LOC339926 (LOC339926), non-coding RNA [NR_038990]
FAM9C	-4,58329	3,81E-04	Homo sapiens family with sequence similarity 9, member C (FAM9C), mRNA [NM_174901]
PCDH15	-4,80083	3,82E-04	Homo sapiens mRNA; cDNA DKFZp667A1711 (from clone DKFZp667A1711), [AL834134]
LOC554201	-4,9401	3,82E-04	Homo sapiens uncharacterized LOC554201 (LOC554201), non-coding RNA [NR_038850]
PROZ	-4,94769	3,82E-04	Homo sapiens protein Z, vitamin K-dependent plasma glycoprotein (PROZ), mRNA [NM_003891]
CXorf1	-4,93698	3,83E-04	Homo sapiens chromosome X open reading frame 1 (CXorf1), mRNA [NM_004709]
LOC100189589	-4,76376	3,83E-04	Homo sapiens uncharacterized LOC100189589 (LOC100189589), non-coding RNA [NR_024463]
C10orf120	-4,55311	3,84E-04	Homo sapiens chromosome 10 open reading frame 120 (C10orf120), mRNA [NM_001010912]
PHOX2A	-4,76011	3,84E-04	Homo sapiens paired-like homeobox 2a (PHOX2A), mRNA [NM_005169]
CD96	-5,12897	3,85E-04	Homo sapiens CD96 molecule (CD96), transcript variant 1, mRNA [NM_198196]
B2M	-1,68068	3,86E-04	Homo sapiens beta-2-microglobulin (B2M), mRNA [NM_004048]
SLC10A7	-4,84454	3,86E-04	Homo sapiens solute carrier family 10 (sodium/bile acid cotransporter family), member 7, mRNA [NM_032128]
CHODL-AS1	-4,92317	3,86E-04	Homo sapiens CHODL antisense RNA 1 (non-protein coding) (CHODL-AS1), non-coding RNA [NR_024354]
LOC728543	-4,94594	3,86E-04	Homo sapiens cDNA FLJ35513 fis, clone SPLEN2000516, [AK092832]
SLCO1B1	-4,95171	3,87E-04	Homo sapiens solute carrier organic anion transporter family, member 1B1 (SLCO1B1), mRNA [NM_006446]
PRO1596	-4,74172	3,87E-04	Homo sapiens PRO3090 mRNA, complete cds, [AF119915]
VASP	-1,52909	3,88E-04	Homo sapiens vasodilator-stimulated phosphoprotein (VASP), mRNA [NM_003370]
PUM1	-1,26923	3,89E-04	Homo sapiens pumilio homolog 1 (Drosophila) (PUM1), transcript variant 1, mRNA [NM_001020658]
TMEM111	1,313819	3,89E-04	Homo sapiens transmembrane protein 111 (TMEM111), mRNA [NM_018447]
PRSS44	-4,39317	3,89E-04	PREDICTED: Homo sapiens protease, serine, 44 (PRSS44), mRNA [XM_001131213]
PTPN20A	-4,76761	3,89E-04	Homo sapiens protein tyrosine phosphatase, non-receptor type 20A (PTPN20A), mRNA [NM_001042387]
XKR6	-4,0258	3,90E-04	Homo sapiens partial mRNA for hypothetical protein (C8orf7 gene), [AJ301560]
STON1-GTF2A1L	-4,84371	3,90E-04	Homo sapiens STON1-GTF2A1L readthrough (STON1-GTF2A1L), transcript variant 2, mRNA [NM_001198593]
CLDN1	-1,28243	3,90E-04	Homo sapiens claudin 1 (CLDN1), mRNA [NM_021101]

TLR8	-4,94409	3,90E-04	Homo sapiens toll-like receptor 8 (TLR8), mRNA [NM_138636]
CEACAM3	-4,86005	3,91E-04	Homo sapiens carcinoembryonic antigen-related cell adhesion molecule 3 (CEACAM3), mRNA [NM_001815]
MX1	-1,78264	3,91E-04	Homo sapiens myxovirus (influenza virus) resistance 1, interferon-inducible protein p78, mRNA [NM_002462]
PLAC8	-3,95488	3,92E-04	Homo sapiens placenta-specific 8 (PLAC8), transcript variant 3, mRNA [NM_001130715]
SLC17A8	-4,77243	3,93E-04	Homo sapiens solute carrier family 17 (sodium-dependent inorganic phosphate cotransporter), mRNA [NM_139319]
FAM153A	-4,36097	3,94E-04	Homo sapiens family with sequence similarity 153, member A (FAM153A), mRNA [NM_173663]
PRPF6	1,263602	3,94E-04	Homo sapiens PRP6 pre-mRNA processing factor 6 homolog (S, cerevisiae) (PRPF6), mRNA [NM_012469]
C21orf91-OT1	-4,64319	3,96E-04	Homo sapiens C21orf91 overlapping transcript 1 (non-protein coding) (C21orf91-OT1), non-coding RNA [NR_038871]
OCR1	-4,84944	3,97E-04	Homo sapiens ovarian cancer-related protein 1 (OCR1) mRNA, complete cds, [AF314543]
C1orf61	-4,746	3,97E-04	chromosome 1 open reading frame 61 [Source:HGNC Symbol;Acc:30780] [ENST00000497824]
C3orf49	-4,77896	3,98E-04	Homo sapiens chromosome 3 open reading frame 49 (C3orf49), non-coding RNA [NR_026866]
C3orf80	-3,95029	3,98E-04	Homo sapiens chromosome 3 open reading frame 80 (C3orf80), mRNA [NM_001168214]
ABHD10	-4,72564	3,98E-04	Homo sapiens abhydrolase domain containing 10 (ABHD10), mRNA [NM_018394]
GBX1	-4,85692	3,98E-04	Homo sapiens gastrulation brain homeobox 1 (GBX1), mRNA [NM_001098834]
MGAT4C	-4,56844	3,98E-04	Homo sapiens mannosyl (alpha-1,3-)-glycoprotein beta-1,4-N-acetylglucosaminyltransferase, mRNA [NM_013244]
LOC646851	-4,69753	4,00E-04	Uncharacterized protein [Source:UniProtKB/TrEMBL;Acc:F5H1G0] [ENST00000540952]
DCHS2	-4,8784	4,00E-04	Homo sapiens dachsous 2 (Drosophila) (DCHS2), transcript variant 3, mRNA [NM_001142553]
ERMN	-4,89692	4,00E-04	Homo sapiens ermin, ERM-like protein (ERMN), transcript variant 2, mRNA [NM_020711]
LOC100134365	-4,77624	4,02E-04	PREDICTED: Homo sapiens hypothetical LOC100134365 (LOC100134365), miscRNA [XR_112366]
ZIC2	-4,56289	4,02E-04	Homo sapiens Zic family member 2 (ZIC2), mRNA [NM_007129]
HCN4	-4,93347	4,02E-04	Homo sapiens hyperpolarization activated cyclic nucleotide-gated potassium channel 4 (HCN4), mRNA [NM_005477]
CUL4A	1,117765	4,02E-04	Homo sapiens cullin 4A (CUL4A), transcript variant 1, mRNA [NM_001008895]
FLJ42842	-4,80492	4,03E-04	PREDICTED: Homo sapiens hypothetical FLJ42842 (FLJ42842), miscRNA [XR_109407]
DCAF8L1	-4,10414	4,03E-04	Homo sapiens DDB1 and CUL4 associated factor 8-like 1 (DCAF8L1), mRNA [NM_001017930]
TMC1	-4,76893	4,03E-04	Homo sapiens transmembrane channel-like 1 (TMC1), mRNA [NM_138691]
MED12L	-4,57979	4,04E-04	mediator complex subunit 12-like [Source:HGNC Symbol;Acc:16050] [ENST00000309237]
MTIF2	-4,89052	4,04E-04	mitochondrial translational initiation factor 2 [Source:HGNC Symbol;Acc:7441] [ENST00000404297]
SLIT1	-4,80234	4,04E-04	Homo sapiens slit homolog 1 (Drosophila) (SLIT1), mRNA [NM_003061]
GNPAT	1,359605	4,05E-04	Homo sapiens glyceronephosphate O-acyltransferase (GNPAT), mRNA [NM_014236]

FHAD1	-4,7471	4,06E-04	forkhead-associated (FHA) phosphopeptide binding domain 1 [Source:HGNC Symbol;Acc:29408] [ENST00000483120]
FLJ45139	-4,88106	4,06E-04	PREDICTED: Homo sapiens FLJ45139 protein (FLJ45139), miscRNA [XR_109674]
OR8K5	-3,86339	4,07E-04	Homo sapiens olfactory receptor, family 8, subfamily K, member 5 (OR8K5), mRNA [NM_001004058]
BTNL9	-4,8516	4,07E-04	Homo sapiens butyrophilin-like 9 (BTNL9), mRNA [NM_152547]
RNASE10	-4,90308	4,08E-04	Homo sapiens ribonuclease, RNase A family, 10 (non-active) (RNASE10), mRNA [NM_001012975]
MYO3A	-4,91874	4,09E-04	Homo sapiens myosin IIIA (MYO3A), mRNA [NM_017433]
TINAG	-4,683	4,09E-04	Homo sapiens tubulointerstitial nephritis antigen (TINAG), mRNA [NM_014464]
F11	-4,48763	4,09E-04	Homo sapiens coagulation factor XI (F11), mRNA [NM_000128]
SLC25A48	-4,8668	4,10E-04	solute carrier family 25, member 48 [Source:HGNC Symbol;Acc:30451] [ENST00000274513]
AFF2	-4,80683	4,10E-04	AF4/FMR2 family, member 2 [Source:HGNC Symbol;Acc:3776] [ENST00000370458]
LOC284648	-3,9744	4,10E-04	Homo sapiens uncharacterized LOC284648 (LOC284648), non-coding RNA [NR_036490]
FAM184B	-3,34903	4,11E-04	Homo sapiens family with sequence similarity 184, member B (FAM184B), mRNA [NM_015688]
MGC15885	-5,12934	4,11E-04	Homo sapiens uncharacterized protein MGC15885 (MGC15885), non-coding RNA [NR_026897]
GLRA3	-4,80257	4,11E-04	Homo sapiens glycine receptor, alpha 3 (GLRA3), transcript variant 1, mRNA [NM_006529]
TMEM121	-1,13258	4,12E-04	Homo sapiens transmembrane protein 121 (TMEM121), mRNA [NM_025268]
KRTAP8-1	-4,91746	4,12E-04	Homo sapiens keratin associated protein 8-1 (KRTAP8-1), mRNA [NM_175857]
ADM	-1,91045	4,13E-04	Homo sapiens adrenomedullin (ADM), mRNA [NM_001124]
TPH2	-4,90524	4,14E-04	Homo sapiens tryptophan hydroxylase 2 (TPH2), mRNA [NM_173353]
LOC100130642	-3,94356	4,15E-04	Homo sapiens cDNA FLJ42255 fis, clone TKIDN2009889, [AK124249]
C1orf74	1,226781	4,15E-04	Homo sapiens chromosome 1 open reading frame 74 (C1orf74), mRNA [NM_152485]
LOC339298	-4,75652	4,15E-04	Homo sapiens uncharacterized LOC339298 (LOC339298), non-coding RNA [NR_040034]
CXorf61	-4,44941	4,15E-04	Homo sapiens chromosome X open reading frame 61 (CXorf61), mRNA [NM_001017978]
LOC100129413	-5,07008	4,16E-04	PREDICTED: Homo sapiens hypothetical LOC100129413 (LOC100129413), miscRNA [XR_110460]
HGC6,3	-5,20922	4,16E-04	Homo sapiens uncharacterized LOC100128124 (HGC6,3), mRNA [NM_001129895]
C8orf86	-4,56592	4,16E-04	Homo sapiens chromosome 8 open reading frame 86 (C8orf86), mRNA [NM_207412]
ZFP64	-4,54595	4,17E-04	zinc finger protein 64 homolog (mouse) [Source:HGNC Symbol;Acc:15940] [ENST00000395979]
PPM1H	-4,88437	4,17E-04	Homo sapiens protein phosphatase, Mg2+/Mn2+ dependent, 1H (PPM1H), mRNA [NM_020700]
HIGD1C	-4,86297	4,20E-04	Homo sapiens HIG1 hypoxia inducible domain family, member 1C (HIGD1C), mRNA [NM_001109619]
LOC641365	-4,05226	4,20E-04	Homo sapiens uncharacterized LOC641365 (LOC641365), non-coding RNA [NR_037866]
FLJ40453	-4,67798	4,21E-04	Homo sapiens cDNA FLJ40453 fis, clone TESTI2041330, [AK097772]

ZNF217	-4,9224	4,21E-04	Homo sapiens zinc finger protein 217 (ZNF217), mRNA [NM_006526]
NDUFA9	1,398644	4,21E-04	Homo sapiens NADH dehydrogenase (ubiquinone) 1 alpha subcomplex, mRNA [NM_005002]
UTY	-4,61323	4,22E-04	Homo sapiens ubiquitously transcribed tetratricopeptide repeat gene, Y-linked (UTY), mRNA [NM_007125]
FOXI2	1,222512	4,22E-04	Homo sapiens forkhead box I2 (FOXI2), mRNA [NM_207426]
C14orf119	-4,78744	4,23E-04	Homo sapiens chromosome 14 open reading frame 119 (C14orf119), mRNA [NM_017924]
LOC283484	-3,76922	4,24E-04	Homo sapiens cDNA FLJ33542 fis, clone BRAMY2007753, [AK090861]
STEAP4	-4,76164	4,24E-04	Homo sapiens STEAP family member 4 (STEAP4), transcript variant 2, mRNA [NM_001205315]
CRISP2	-4,6548	4,25E-04	cysteine-rich secretory protein 2 [Source:HGNC Symbol;Acc:12024] [ENST00000211238]
CCDC147	-1,25683	4,26E-04	Homo sapiens coiled-coil domain containing 147 (CCDC147), mRNA [NM_001008723]
PADI4	-4,88083	4,27E-04	Homo sapiens peptidyl arginine deiminase, type IV (PADI4), mRNA [NM_012387]
MACROD2	-1,34877	4,27E-04	MACRO domain containing 2 [Source:HGNC Symbol;Acc:16126] [ENST00000462552]
LOC401220	-4,558	4,27E-04	Homo sapiens hypothetical gene supported by BC036933, mRNA (cDNA clone IMAGE:5243701), [BC036933]
RAD9B	-4,91439	4,29E-04	RAD9 homolog B (S, pombe) [Source:HGNC Symbol;Acc:21700] [ENST00000409461]
CD48	-4,84112	4,30E-04	Homo sapiens CD48 molecule (CD48), mRNA [NM_001778]
XKRY2	-4,9501	4,31E-04	Homo sapiens XK, Kell blood group complex subunit-related, Y-linked 2 (XKRY2), mRNA [NM_001002906]
LOC285456	-4,61924	4,31E-04	Homo sapiens uncharacterized LOC285456 (LOC285456), non-coding RNA [NR_026968]
OR6C4	-4,66059	4,31E-04	Homo sapiens olfactory receptor, family 6, subfamily C, member 4 (OR6C4), mRNA [NM_001005494]
DPPA3	-4,7131	4,32E-04	Homo sapiens developmental pluripotency associated 3 (DPPA3), mRNA [NM_199286]
PARP4	-4,71979	4,33E-04	Homo sapiens poly (ADP-ribose) polymerase family, member 4 (PARP4), mRNA [NM_006437]
CDRT7	-4,69572	4,34E-04	Homo sapiens CMT1A duplicated region transcript 7 (non-protein coding) (CDRT7), non-coding RNA [NR_033371]
PLCZ1	-4,81911	4,35E-04	Homo sapiens phospholipase C, zeta 1 (PLCZ1), mRNA [NM_033123]
EXOC6B	-4,70169	4,36E-04	Homo sapiens mRNA for KIAA0919 protein, partial cds, [AB023136]
RGS13	-5,02902	4,37E-04	Homo sapiens regulator of G-protein signaling 13 (RGS13), transcript variant 1, mRNA [NM_002927]
LOC100132396	-3,99681	4,38E-04	Homo sapiens zinc finger protein 705D-like (LOC100132396), mRNA [NM_001193630]
SCGB1C1	-2,0561	4,39E-04	Homo sapiens secretoglobin, family 1C, member 1 (SCGB1C1), mRNA [NM_145651]
LOC282980	-3,93369	4,39E-04	Homo sapiens uncharacterized LOC282980 (LOC282980), non-coding RNA [NR_040253]
VEGFA	2,208947	4,40E-04	Homo sapiens vascular endothelial growth factor A (VEGFA), transcript variant 1, mRNA [NM_001025366]
TSHR	-4,54204	4,40E-04	Homo sapiens thyroid stimulating hormone receptor (TSHR), transcript variant 2, mRNA [NM_001018036]
KRTAP19-4	-4,88459	4,42E-04	Homo sapiens keratin associated protein 19-4 (KRTAP19-4), mRNA [NM_181610]
KRT81	1,17658	4,42E-04	Homo sapiens keratin 81 (KRT81), mRNA [NM_002281]
TMEM156	-5,09553	4,43E-04	Homo sapiens transmembrane protein 156 (TMEM156), mRNA [NM_024943]

SCN7A	-4,63432	4,44E-04	Homo sapiens sodium channel, voltage-gated, type VII, alpha (SCN7A), mRNA [NM_002976]
WBP2NL	-4,83772	4,44E-04	Homo sapiens WBP2 N-terminal like (WBP2NL), mRNA [NM_152613]
C10orf71	-5,01719	4,44E-04	Homo sapiens chromosome 10 open reading frame 71 (C10orf71), transcript variant 1, mRNA [NM_001135196]
PCDHGA8	-4,79774	4,47E-04	Homo sapiens protocadherin gamma subfamily A, 8 (PCDHGA8), transcript variant 2, mRNA [NM_014004]
DYDC1	-4,77343	4,48E-04	Homo sapiens DPY30 domain containing 1 (DYDC1), mRNA [NM_138812]
C12orf63	-4,9122	4,49E-04	PREDICTED: Homo sapiens chromosome 12 open reading frame 63 (C12orf63), mRNA [XM_003118942]
FBRS	-1,15014	4,49E-04	Homo sapiens fibrosin (FBRS), mRNA [NM_001105079]
ITIH5	-4,88701	4,50E-04	Homo sapiens inter-alpha-trypsin inhibitor heavy chain family, member 5 (ITIH5), mRNA [NM_030569]
FTL	-1,64355	4,50E-04	Homo sapiens ferritin, light polypeptide (FTL), mRNA [NM_000146]
NECAB1	-4,82644	4,52E-04	Homo sapiens N-terminal EF-hand calcium binding protein 1 (NECAB1), mRNA [NM_022351]
GIGYF2	-1,23895	4,52E-04	Homo sapiens GRB10 interacting GYF protein 2 (GIGYF2), transcript variant 2, mRNA [NM_015575]
LOC100129427	-4,15519	4,53E-04	Homo sapiens uncharacterized LOC100129427 (LOC100129427), non-coding RNA [NR_033845]
HNF4G	-4,64394	4,53E-04	Homo sapiens hepatocyte nuclear factor 4, gamma (HNF4G), mRNA [NM_004133]
TTPA	-4,87992	4,55E-04	Homo sapiens tocopherol (alpha) transfer protein (TTPA), mRNA [NM_000370]
HRH4	-4,86606	4,55E-04	Homo sapiens histamine receptor H4 (HRH4), transcript variant 1, mRNA [NM_021624]
MYH4	-4,83467	4,56E-04	Homo sapiens myosin, heavy chain 4, skeletal muscle (MYH4), mRNA [NM_017533]
KIAA1524	-5,1679	4,57E-04	Homo sapiens KIAA1524 (KIAA1524), mRNA [NM_020890]
FHDC1	-4,15103	4,57E-04	Homo sapiens FH2 domain containing 1 (FHDC1), mRNA [NM_033393]
NPY1R	-4,52676	4,58E-04	Homo sapiens neuropeptide Y receptor Y1 (NPY1R), mRNA [NM_000909]
FAM55D	-4,71362	4,58E-04	Homo sapiens family with sequence similarity 55, member D (FAM55D), mRNA [NM_001077639]
TSPAN16	-4,81016	4,58E-04	Homo sapiens tetraspanin 16 (TSPAN16), mRNA [NM_012466]
TRHDE	-4,59133	4,59E-04	Homo sapiens thyrotropin-releasing hormone degrading enzyme (TRHDE), mRNA [NM_013381]
ANKS1B	-4,83701	4,60E-04	Homo sapiens ankyrin repeat and sterile alpha motif domain containing 1B (ANKS1B), mRNA [NM_181670]
SLC16A12	-3,82339	4,60E-04	Homo sapiens solute carrier family 16, member 12 (monocarboxylic acid transporter 12), mRNA [NM_213606]
KLRC1	-4,5361	4,62E-04	Homo sapiens killer cell lectin-like receptor subfamily C, member 1 (KLRC1), mRNA [NM_002259]
EEF1G	-1,77258	4,62E-04	Homo sapiens eukaryotic translation elongation factor 1 gamma (EEF1G), mRNA [NM_001404]
PNPLA1	-4,41978	4,64E-04	Homo sapiens patatin-like phospholipase domain containing 1 (PNPLA1), mRNA [NM_001145717]
ZNF136	-1,19551	4,66E-04	Homo sapiens zinc finger protein 136 (ZNF136), mRNA [NM_003437]
C1orf192	-4,80811	4,66E-04	Homo sapiens chromosome 1 open reading frame 192 (C1orf192), mRNA [NM_001013625]
HECW1	-3,80609	4,66E-04	Homo sapiens HECT, C2 and WW domain containing E3 ubiquitin protein ligase 1 (HECW1), mRNA [NM_015052]
IDO2	-4,53744	4,68E-04	Homo sapiens indoleamine 2,3-dioxygenase 2 (IDO2), mRNA [NM_194294]

DGKB	-4,81159	4,68E-04	Homo sapiens diacylglycerol kinase, beta 90kDa (DGKB), transcript variant 1, mRNA [NM_004080]
ZNF680	-4,79432	4,68E-04	Homo sapiens zinc finger protein 680 (ZNF680), transcript variant 1, mRNA [NM_178558]
PHF2P1	-4,56695	4,69E-04	Homo sapiens PHD finger protein 2 pseudogene 1 (PHF2P1), non-coding RNA [NR_002801]
TPH1	-3,76304	4,69E-04	Homo sapiens tryptophan hydroxylase 1 (TPH1), mRNA [NM_004179]
FRY	-4,20022	4,69E-04	Homo sapiens furry homolog (Drosophila) (FRY), mRNA [NM_023037]
NFIA	-1,36316	4,69E-04	Homo sapiens nuclear factor I/A (NFIA), transcript variant 2, mRNA [NM_005595]
BLNK	-4,62485	4,69E-04	Homo sapiens B-cell linker (BLNK), transcript variant 1, mRNA [NM_013314]
SEMA3E	-4,8881	4,69E-04	Homo sapiens sema domain, immunoglobulin domain (Ig), short basic domain, secreted 3E, mRNA [NM_012431]
FILIP1L	4,217307	4,71E-04	Homo sapiens filamin A interacting protein 1-like (FILIP1L), transcript variant 1, mRNA [NM_182909]
ALDOB	-3,92042	4,71E-04	Homo sapiens aldolase B, fructose-bisphosphate (ALDOB), mRNA [NM_000035]
TPD52L3	-4,55212	4,71E-04	Homo sapiens tumor protein D52-like 3 (TPD52L3), transcript variant 1, mRNA [NM_033516]
KCNIP1	-4,86862	4,73E-04	Homo sapiens Kv channel interacting protein 1 (KCNIP1), transcript variant 1, mRNA [NM_001034837]
TERF1	-4,85823	4,73E-04	Homo sapiens telomeric repeat binding factor (NIMA-interacting) 1 (TERF1), mRNA [NM_017489]
NPHP3-AS1	-4,70747	4,73E-04	Homo sapiens NPHP3 antisense RNA 1 (non-protein coding) (NPHP3-AS1), non-coding RNA [NR_002811]
ITGB1BP1	-3,90406	4,74E-04	integrin beta 1 binding protein 1 [Source:HGNC Symbol;Acc:23927] [ENST00000360635]
LOC100652763	-4,84992	4,74E-04	PREDICTED: Homo sapiens hypothetical protein LOC100652763 (LOC100652763), mRNA [XM_003403488]
PIEZO2	-4,82485	4,75E-04	Homo sapiens cDNA FLJ34907 fis, clone NT2RI2003392, [AK092226]
AGBL4	-4,78417	4,76E-04	ATP/GTP binding protein-like 4 [Source:HGNC Symbol;Acc:25892] [ENST00000371836]
RWDD3	-1,10118	4,76E-04	Homo sapiens RWD domain containing 3 (RWDD3), transcript variant 1, mRNA [NM_015485]
HSD17B3	-4,50562	4,77E-04	Homo sapiens hydroxysteroid (17-beta) dehydrogenase 3 (HSD17B3), mRNA [NM_000197]
POLL	1,087119	4,77E-04	Homo sapiens polymerase (DNA directed), lambda (POLL), transcript variant 1, mRNA [NM_001174084]
LOC285326	-5,11191	4,77E-04	Homo sapiens uncharacterized LOC285326 (LOC285326), non-coding RNA [NR_034055]
MUC19	-3,73661	4,77E-04	PREDICTED: Homo sapiens mucin-19-like (LOC100652946), mRNA [XM_003403524]
LOC389834	-4,841	4,78E-04	Homo sapiens ankyrin repeat domain 57 pseudogene (LOC389834), non-coding RNA [NR_027420]
C14orf39	-4,84088	4,78E-04	Homo sapiens chromosome 14 open reading frame 39 (C14orf39), mRNA [NM_174978]
BRCC3	-1,42523	4,79E-04	Homo sapiens BRCA1/BRCA2-containing complex, subunit 3 (BRCC3), transcript variant 1, mRNA [NM_024332]
UBE2E3	-4,93913	4,80E-04	ubiquitin-conjugating enzyme E2E 3 [Source:HGNC Symbol;Acc:12479] [ENST00000409513]
UBE2CBP	-4,80692	4,82E-04	ubiquitin-conjugating enzyme E2C binding protein [Source:HGNC Symbol;Acc:21381] [ENST00000369746]
KLF11	-4,93669	4,83E-04	Kruppel-like factor 11 [Source:HGNC Symbol;Acc:11811] [ENST00000401510]
TEC	-4,27849	4,83E-04	Homo sapiens tec protein tyrosine kinase (TEC), mRNA [NM_003215]
C3orf77	-5,19008	4,83E-04	Homo sapiens chromosome 3 open reading frame 77 (C3orf77), mRNA [NM_001145030]

CCDC144NL	-3,77416	4,85E-04	Homo sapiens coiled-coil domain containing 144 family, N-terminal like (CCDC144NL), mRNA [NM_001004306]
HLA-J	-1,48084	4,85E-04	Homo sapiens major histocompatibility complex, class I, J (pseudogene) (HLA-J), non-coding RNA [NR_024240]
TEKT1	-5,20015	4,86E-04	Homo sapiens tektin 1 (TEKT1), mRNA [NM_053285]
PTPMT1	1,235735	4,86E-04	Homo sapiens protein tyrosine phosphatase, mitochondrial 1 (PTPMT1), mRNA [NM_175732]
SEMG2	-5,12864	4,86E-04	Homo sapiens semenogelin II (SEMG2), mRNA [NM_003008]
ZNF740	-1,31173	4,87E-04	Homo sapiens zinc finger protein 740 (ZNF740), mRNA [NM_001004304]
OR5M9	-5,16251	4,87E-04	Homo sapiens olfactory receptor, family 5, subfamily M, member 9 (OR5M9), mRNA [NM_001004743]
HOXD3	-4,69511	4,88E-04	homeobox D3 [Source:HGNC Symbol;Acc:5137] [ENST00000459979]
LOC647020	-5,11734	4,89E-04	PREDICTED: Homo sapiens hypothetical protein LOC647020 (LOC647020), mRNA [XM_001719614]
HOXB7	-4,79196	4,89E-04	Homo sapiens homeobox B7 (HOXB7), mRNA [NM_004502]
C13orf26	-5,06191	4,92E-04	Homo sapiens chromosome 13 open reading frame 26 (C13orf26), mRNA [NM_152325]
C14orf165	-4,97863	4,92E-04	Homo sapiens chromosome 14 open reading frame 165 (C14orf165), non-coding RNA [NR_024081]
DCAF4L1	-4,54921	4,93E-04	Homo sapiens DDB1 and CUL4 associated factor 4-like 1 (DCAF4L1), mRNA [NM_001029955]
DACT2	-4,83591	4,93E-04	Homo sapiens dapper, antagonist of beta-catenin, homolog 2 (Xenopus laevis) (DACT2), mRNA [NM_214462]
RPL31	-1,54275	4,93E-04	Homo sapiens ribosomal protein L31 (RPL31), transcript variant 1, mRNA [NM_000993]
LOC401324	-4,67944	4,96E-04	Homo sapiens uncharacterized LOC401324 (LOC401324), non-coding RNA [NR_038864]
F13A1	-4,74146	4,98E-04	Homo sapiens coagulation factor XIII, A1 polypeptide (F13A1), mRNA [NM_000129]
ENAH	-3,84875	4,99E-04	enabled homolog (Drosophila) [Source:HGNC Symbol;Acc:18271] [ENST00000498108]
EFCAB3	-3,77107	5,04E-04	Homo sapiens EF-hand calcium binding domain 3 (EFCAB3), transcript variant 1, mRNA [NM_001144933]
ZNF479	-3,90415	5,06E-04	Homo sapiens zinc finger protein 479 (ZNF479), mRNA [NM_033273]
DKFZP547J0410	-4,64645	5,06E-04	Homo sapiens mRNA; cDNA DKFZp564J0482 (from clone DKFZp564J0482) [AL050263]
LOC100628314	-3,73137	5,07E-04	Homo sapiens, mRNA sequence [BQ013595]
CARD8	-1,23663	5,08E-04	Homo sapiens caspase recruitment domain family, member 8 (CARD8), transcript variant 2, mRNA [NM_014959]
DNAJA4	1,109032	5,10E-04	Homo sapiens DnaJ (Hsp40) homolog, subfamily A, member 4 (DNAJA4), transcript variant 1, mRNA [NM_018602]
JSRP1	-1,26803	5,10E-04	Homo sapiens junctional sarcoplasmic reticulum protein 1 (JSRP1), mRNA [NM_144616]
SLC30A5	-5,62576	5,12E-04	Homo sapiens solute carrier family 30 (zinc transporter), member 5 (SLC30A5), mRNA [NM_024055]
RGS5	-1,28784	5,13E-04	Homo sapiens regulator of G-protein signaling 5 (RGS5), transcript variant 1, mRNA [NM_003617]
FLJ41455	-3,75262	5,14E-04	Homo sapiens cDNA FLJ41455 fis, clone BRSTN2012284, [AK123449]
MAP7D2	-3,95364	5,17E-04	Homo sapiens MAP7 domain containing 2 (MAP7D2), transcript variant 2, mRNA [NM_152780]
RPL38	-1,94667	5,18E-04	Homo sapiens ribosomal protein L38 (RPL38), transcript variant 1, mRNA [NM_000999]
RPAP2	-1,30254	5,19E-04	Homo sapiens RNA polymerase II associated protein 2 (RPAP2), mRNA [NM_024813]

LOC100507605	-5,12316	5,19E-04	Homo sapiens cDNA clone IMAGE:4828005, [BC028422]
TM6SF1	-4,54189	5,19E-04	Homo sapiens transmembrane 6 superfamily member 1 (TM6SF1), transcript variant 1, mRNA [NM_023003]
GPR110	-3,88805	5,20E-04	Homo sapiens G protein-coupled receptor 110 (GPR110), transcript variant 2, mRNA [NM_025048]
PHF15	-5,10934	5,21E-04	Homo sapiens PHD finger protein 15 (PHF15), mRNA [NM_015288]
KRT40	-4,544	5,22E-04	Homo sapiens keratin 40 (KRT40), mRNA [NM_182497]
C9orf135	-5,05762	5,23E-04	Homo sapiens chromosome 9 open reading frame 135 (C9orf135), mRNA [NM_001010940]
HOGA1	-3,04942	5,27E-04	Homo sapiens 4-hydroxy-2-oxoglutarate aldolase 1 (HOGA1), mRNA [NM_138413]
LOC401177	-4,70166	5,29E-04	Homo sapiens uncharacterized LOC401177 (LOC401177), non-coding RNA [NR_033975]
TRAPPC2L	1,538958	5,32E-04	Homo sapiens trafficking protein particle complex 2-like (TRAPPC2L), mRNA [NM_016209]
LINC00207	-4,57663	5,33E-04	Homo sapiens long intergenic non-protein coding RNA 207 (LINC00207), non-coding RNA [NR_028409]
CNTRL	-1,86413	5,34E-04	centriolin [Source:HGNC Symbol;Acc:1858] [ENST00000373847]
SLC2A7	-4,54889	5,34E-04	Homo sapiens solute carrier family 2 (facilitated glucose transporter), member 7 (SLC2A7), mRNA [NM_207420]
CALM2	-1,83672	5,35E-04	Homo sapiens calmodulin 2 (phosphorylase kinase, delta) (CALM2), mRNA [NM_001743]
RHPN2	-1,14236	5,35E-04	Homo sapiens rhophilin, Rho GTPase binding protein 2 (RHPN2), mRNA [NM_033103]
EIF2B3	1,169739	5,37E-04	Homo sapiens eukaryotic translation initiation factor 2B, subunit 3 gamma, 58kDa (EIF2B3), mRNA [NM_020365]
ECSIT	1,258792	5,39E-04	Homo sapiens ECSIT homolog (Drosophila), nuclear gene encoding mitochondrial protein, mRNA [NM_016581]
ABCC2	-4,62774	5,40E-04	ATP-binding cassette, sub-family C (CFTR/MRP), member 2 [Source:HGNC Symbol;Acc:53] [ENST00000370434]
TPTE2P1	-4,61246	5,42E-04	Homo sapiens transmembrane phosphoinositide 3-phosphatase and tensin homolog 2 pseudogene 1, [NR_026730]
RPL12	-1,61117	5,42E-04	Homo sapiens ribosomal protein L12 (RPL12), mRNA [NM_000976]
LOC404266	-5,029	5,43E-04	Homo sapiens uncharacterized LOC404266 (LOC404266), transcript variant 1, non-coding RNA [NR_033201]
WHSC1	-4,61945	5,44E-04	Homo sapiens Wolf-Hirschhorn syndrome candidate 1 (WHSC1), transcript variant 7, mRNA [NM_133334]
KIAA1377	-4,76222	5,44E-04	Homo sapiens KIAA1377 (KIAA1377), mRNA [NM_020802]
CD226	-5,08403	5,45E-04	Homo sapiens CD226 molecule (CD226), mRNA [NM_006566]
NEUROD4	-3,70105	5,47E-04	Homo sapiens neurogenic differentiation 4 (NEUROD4), mRNA [NM_021191]
ADH6	-5,03312	5,48E-04	Homo sapiens alcohol dehydrogenase 6 (class V) (ADH6), transcript variant 1, mRNA [NM_001102470]
GVINP1	-4,96396	5,48E-04	Homo sapiens GTPase, very large interferon inducible pseudogene 1 (GVINP1), non-coding RNA [NR_003945]
RPL21P44	1,349061	5,49E-04	Homo sapiens ribosomal protein L21 pseudogene 44 (RPL21P44), non-coding RNA [NR_027153]
PCDHB5	-3,91467	5,50E-04	Homo sapiens protocadherin beta 5 (PCDHB5), mRNA [NM_015669]
TIGD4	-4,9963	5,50E-04	Homo sapiens tigger transposable element derived 4 (TIGD4), mRNA [NM_145720]
TFPT	1,259067	5,51E-04	Homo sapiens TCF3 (E2A) fusion partner (in childhood Leukemia) (TFPT), mRNA [NM_013342]

TTC23L	-3,92558	5,53E-04	Homo sapiens tetratricopeptide repeat domain 23-like (TTC23L), mRNA [NM_144725]
C17orf104	-4,47934	5,55E-04	chromosome 17 open reading frame 104 [Source:HGNC Symbol;Acc:26670] [ENST00000409464]
LOC100131298	-4,99386	5,56E-04	Homo sapiens clone FLB4228 PRO1095 mRNA, complete cds, [AF130053]
OR6B1	-4,60588	5,56E-04	Homo sapiens olfactory receptor, family 6, subfamily B, member 1 (OR6B1), mRNA [NM_001005281]
SIRT7	1,191698	5,59E-04	Homo sapiens sirtuin 7 (SIRT7), mRNA [NM_016538]
RNMTL1	1,210659	5,62E-04	Homo sapiens RNA methyltransferase like 1 (RNMTL1), mRNA [NM_018146]
LPHN3	-4,57996	5,65E-04	Homo sapiens latrophilin 3 (LPHN3), mRNA [NM_015236]
LINC00307	-3,84882	5,67E-04	Homo sapiens long intergenic non-protein coding RNA 307 (LINC00307), non-coding RNA [NR_038855]
CNPY3	1,225457	5,68E-04	Homo sapiens canopy 3 homolog (zebrafish) (CNPY3), mRNA [NM_006586]
KSR2	-4,17664	5,69E-04	Homo sapiens kinase suppressor of ras 2 (KSR2), mRNA [NM_173598]
AS3MT	-1,67785	5,69E-04	Homo sapiens arsenic (+3 oxidation state) methyltransferase (AS3MT), mRNA [NM_020682]
INPP5D	-1,20273	5,69E-04	Homo sapiens inositol polyphosphate-5-phosphatase, 145kDa (INPP5D), mRNA [NM_001017915]
LOC284630	-3,92757	5,70E-04	Homo sapiens cDNA FLJ39065 fis, clone NT2RP7014721, [AK096384]
CXorf51A	-4,65749	5,72E-04	Homo sapiens chromosome X open reading frame 51A (CXorf51A), mRNA [NM_001144064]
PCIF1	1,141843	5,72E-04	Homo sapiens PDX1 C-terminal inhibiting factor 1 (PCIF1), mRNA [NM_022104]
CNTNAP2	-4,97881	5,73E-04	Homo sapiens contactin associated protein-like 2 (CNTNAP2), mRNA [NM_014141]
RASL12	-3,94707	5,73E-04	Homo sapiens RAS-like, family 12 (RASL12), mRNA [NM_016563]
LILRB2	-4,62189	5,76E-04	Homo sapiens leukocyte immunoglobulin-like receptor, subfamily B, member 2 (LILRB2), mRNA [NM_005874]
PPP1R3A	-4,96762	5,76E-04	Homo sapiens protein phosphatase 1, regulatory subunit 3A (PPP1R3A), mRNA [NM_002711]
DFNB31	-1,63617	5,76E-04	deafness, autosomal recessive 31 [Source:HGNC Symbol;Acc:16361] [ENST00000374057]
LOC100130157	-4,60365	5,76E-04	PREDICTED: Homo sapiens hypothetical LOC100130157 (LOC100130157), miscRNA [XR_109613]
CDC42BPB	-1,3475	5,78E-04	Homo sapiens CDC42 binding protein kinase beta (DMPK-like) (CDC42BPB), mRNA [NM_006035]
RPL5	-1,9583	5,79E-04	Homo sapiens ribosomal protein L5 (RPL5), mRNA [NM_000969]
ITGA10	-1,3169	5,79E-04	Homo sapiens integrin, alpha 10 (ITGA10), mRNA [NM_003637]
LOC100128001	-3,70502	5,82E-04	Homo sapiens cDNA FLJ43732 fis, clone TESTI1000491, [AK125720]
FNBP1	-1,4402	5,83E-04	formin binding protein 1 [Source:HGNC Symbol;Acc:17069] [ENST00000355681]
CD37	-3,69363	5,85E-04	CD37 molecule [Source:HGNC Symbol;Acc:1666] [ENST00000391859]
LINC00324	-4,19787	5,85E-04	Homo sapiens long intergenic non-protein coding RNA 324 (LINC00324), non-coding RNA [NR_026951]
PSME4	-4,53438	5,86E-04	proteasome (prosome, macropain) activator subunit 4 [Source:HGNC Symbol;Acc:20635] [ENST00000488687]
C20orf202	-4,04506	5,88E-04	Homo sapiens chromosome 20 open reading frame 202 (C20orf202), mRNA [NM_001009612]
SFXN1	-3,90432	5,88E-04	Homo sapiens sideroflexin 1 (SFXN1), mRNA [NM_022754]

ABCA10	-3,82956	5,89E-04	Homo sapiens ATP-binding cassette, sub-family A (ABC1), member 10 (ABCA10), mRNA [NM_080282]
FABP9	-3,84781	5,91E-04	Homo sapiens fatty acid binding protein 9, testis (FABP9), mRNA [NM_001080526]
DAZ2	-4,53869	5,93E-04	Homo sapiens deleted in azoospermia 2 (DAZ2), transcript variant 2, mRNA [NM_001005785]
RBMX2	1,348491	5,93E-04	Homo sapiens RNA binding motif protein, X-linked 2 (RBMX2), mRNA [NM_016024]
OR52N2	-3,92152	5,95E-04	Homo sapiens olfactory receptor, family 52, subfamily N, member 2 (OR52N2), mRNA [NM_001005174]
PAF1	1,334445	5,95E-04	Homo sapiens Paf1, RNA polymerase II associated factor, homolog (S, cerevisiae) (PAF1), mRNA [NM_019088]
MRPS10	1,226666	5,96E-04	Homo sapiens mitochondrial ribosomal protein S10 (MRPS10), mRNA [NM_018141]
C10orf113	-4,63292	5,96E-04	Homo sapiens chromosome 10 open reading frame 113 (C10orf113), transcript variant 2, mRNA [NM_001177483]
LOC441242	-4,03638	5,98E-04	Homo sapiens uncharacterized LOC441242 (LOC441242), non-coding RNA [NR_038378]
CD109	-1,20987	6,00E-04	Homo sapiens CD109 molecule (CD109), transcript variant 1, mRNA [NM_133493]
JAKMIP2	-4,27294	6,01E-04	Homo sapiens janus kinase and microtubule interacting protein 2 (JAKMIP2), mRNA [NM_014790]
P4HB	-1,60974	6,02E-04	Homo sapiens prolyl 4-hydroxylase, beta polypeptide (P4HB), mRNA [NM_000918]
SAFB2	-1,20562	6,03E-04	Homo sapiens scaffold attachment factor B2 (SAFB2), mRNA [NM_014649]
FAM84B	-4,50575	6,03E-04	Homo sapiens family with sequence similarity 84, member B (FAM84B), mRNA [NM_174911]
TAT	-2,32262	6,04E-04	Homo sapiens tyrosine aminotransferase (TAT), nuclear gene encoding mitochondrial protein, mRNA [NM_000353]
IQUB	-4,39402	6,06E-04	Homo sapiens IQ motif and ubiquitin domain containing (IQUB), mRNA [NM_178827]
AKNAD1	-5,00579	6,07E-04	Homo sapiens AKNA domain containing 1 (AKNAD1), mRNA [NM_152763]
PAR5	-4,86993	6,08E-04	Homo sapiens Prader-Willi/Angelman syndrome-5 (PAR5), non-coding RNA [NR_022008]
BCL7A	-1,34794	6,08E-04	Homo sapiens B-cell CLL/lymphoma 7A (BCL7A), transcript variant 1, mRNA [NM_020993]
RASSF9	-3,73736	6,09E-04	Homo sapiens Ras association (RalGDS/AF-6) domain family (N-terminal) member 9 (RASSF9), mRNA [NM_005447]
LOC286370	-4,52547	6,10E-04	Homo sapiens uncharacterized LOC286370 (LOC286370), non-coding RNA [NR_038882]
TTTY21	-3,66734	6,11E-04	Homo sapiens testis-specific transcript, Y-linked 21 (non-protein coding) (TTTY21), non-coding RNA [NR_001535]
RNU105A	-1,12613	6,12E-04	Homo sapiens RNA, U105A small nucleolar (RNU105A), small nucleolar RNA [NR_004404]
BPNT1	-4,79513	6,14E-04	Homo sapiens 3'(2'), 5'-bisphosphate nucleotidase 1 (BPNT1), mRNA [NM_006085]
TCOF1	1,194456	6,14E-04	Homo sapiens Treacher Collins-Franceschetti syndrome 1 (TCOF1), transcript variant 4, mRNA [NM_001135243]
LOC100132147	-4,07015	6,17E-04	Homo sapiens cDNA clone IMAGE:4816083, partial cds, [BC036435]
CRYAA	-5,20219	6,18E-04	Homo sapiens crystallin, alpha A (CRYAA), mRNA [NM_000394]
CD44	-1,88426	6,20E-04	Homo sapiens CD44 molecule (Indian blood group) (CD44), transcript variant 1, mRNA [NM_000610]
C11orf16	-4,61073	6,22E-04	Homo sapiens chromosome 11 open reading frame 16 (C11orf16), mRNA [NM_020643]
ARHGAP36	-3,73948	6,23E-04	Homo sapiens Rho GTPase activating protein 36 (ARHGAP36), mRNA [NM_144967]
PSMC5	1,526539	6,23E-04	Homo sapiens proteasome (prosome, macropain) 26S subunit, ATPase, 5 (PSMC5), mRNA [NM_002805]

APOB	-4,54046	6,24E-04	Homo sapiens apolipoprotein B (including Ag(x) antigen) (APOB), mRNA [NM_000384]
RAMP2	-3,74348	6,27E-04	Homo sapiens receptor (G protein-coupled) activity modifying protein 2 (RAMP2), mRNA [NM_005854]
LOC646168	-4,94626	6,29E-04	Homo sapiens uncharacterized LOC646168 (LOC646168), non-coding RNA [NR_033843]
C20orf4	1,154887	6,29E-04	Homo sapiens chromosome 20 open reading frame 4 (C20orf4), mRNA [NM_015511]
LOC441493	-4,50476	6,29E-04	PREDICTED: Homo sapiens hypothetical LOC441493 (LOC441493), miscRNA [XR_113304]
C1orf173	-4,20351	6,30E-04	Homo sapiens chromosome 1 open reading frame 173 (C1orf173), mRNA [NM_001002912]
NKX2-4	-4,52781	6,32E-04	Homo sapiens NK2 homeobox 4 (NKX2-4), mRNA [NM_033176]
SNCAIP	-4,64573	6,33E-04	synuclein, alpha interacting protein [Source:HGNC Symbol;Acc:11139] [ENST00000395469]
PVR	1,39524	6,33E-04	Homo sapiens poliovirus receptor (PVR), transcript variant 1, mRNA [NM_006505]
LMOD3	-4,59992	6,34E-04	Homo sapiens leiomodin 3 (fetal) (LMOD3), mRNA [NM_198271]
CTSS	-4,52061	6,40E-04	Homo sapiens cathepsin S (CTSS), transcript variant 1, mRNA [NM_004079]
RPL23A	-1,43446	6,41E-04	Homo sapiens ribosomal protein L23a (RPL23A), mRNA [NM_000984]
MSTN	-4,80004	6,42E-04	Homo sapiens myostatin (MSTN), mRNA [NM_005259]
NRXN1	-4,58543	6,43E-04	Homo sapiens neurexin 1 (NRXN1), transcript variant alpha2, mRNA [NM_001135659]
ANKRD55	-3,7599	6,45E-04	Homo sapiens ankyrin repeat domain 55 (ANKRD55), mRNA [NM_024669]
TAPBP	1,343094	6,48E-04	Homo sapiens TAP binding protein (tapasin) (TAPBP), transcript variant 1, mRNA [NM_003190]
EEF1A1	1,3737	6,49E-04	Homo sapiens eukaryotic translation elongation factor 1 alpha 1 (EEF1A1), mRNA [NM_001402]
LOC100131096	-4,1151	6,50E-04	Homo sapiens uncharacterized LOC100131096 (LOC100131096), non-coding RNA [NR_040071]
LOC441167	-4,51483	6,51E-04	PREDICTED: Homo sapiens hCG1820801 (LOC441167), miscRNA [XR_110213]
CYP4B1	-3,56682	6,51E-04	Homo sapiens cytochrome P450, family 4, subfamily B, polypeptide 1 (CYP4B1), mRNA [NM_000779]
KRTAP10-11	-4,03239	6,51E-04	Homo sapiens keratin associated protein 10-11 (KRTAP10-11), mRNA [NM_198692]
FLYWCH2	-3,9127	6,53E-04	Homo sapiens FLYWCH family member 2 (FLYWCH2), transcript variant 1, mRNA [NM_138439]
WHSC1	1,179843	6,54E-04	Homo sapiens Wolf-Hirschhorn syndrome candidate 1 (WHSC1), transcript variant 1, mRNA [NM_133330]
ANKRD62P1-PARP4P3	-4,56499	6,55E-04	Homo sapiens ANKRD62P1-PARP4P3 readthrough (non-protein coding) (ANKRD62P1-PARP4P3), [NR_040115]
MAGEB3	-4,90183	6,55E-04	Homo sapiens melanoma antigen family B, 3 (MAGEB3), mRNA [NM_002365]
C10orf118	-5,02441	6,56E-04	chromosome 10 open reading frame 118 [Source:HGNC Symbol;Acc:24349] [ENST00000369285]
SLC6A1	-4,53358	6,58E-04	Homo sapiens solute carrier family 6 (neurotransmitter transporter, GABA), member 1, mRNA [NM_003042]
LOC285281	-4,64811	6,59E-04	Homo sapiens cDNA clone IMAGE:5264326, [BC035141]
C3orf80	-4,54299	6,61E-04	Homo sapiens chromosome 3 open reading frame 80 (C3orf80), mRNA [NM_001168214]
NHSL2	-3,80987	6,62E-04	Homo sapiens NHS-like 2 (NHSL2), mRNA [NM_001013627]

PDX1	-4,71634	6,63E-04	Homo sapiens pancreatic and duodenal homeobox 1 (PDX1), mRNA [NM_000209]
FLJ37035	-3,99491	6,64E-04	Homo sapiens uncharacterized LOC399821 (FLJ37035), non-coding RNA [NR_033847]
LOC646999	-1,26407	6,67E-04	Homo sapiens akirin 1 pseudogene (LOC646999), non-coding RNA [NR_024390]
NR3C2	-2,71364	6,68E-04	Homo sapiens nuclear receptor subfamily 3, group C, member 2 (NR3C2), transcript variant 1, mRNA [NM_000901]
CLDN10	-4,95407	6,69E-04	Homo sapiens claudin 10 (CLDN10), transcript variant a, mRNA [NM_182848]
C14orf48	-3,52236	6,69E-04	Homo sapiens chromosome 14 open reading frame 48 (C14orf48), transcript variant 3, [NR_024184]
RBMXL3	-4,70599	6,70E-04	Homo sapiens RNA binding motif protein, X-linked-like 3 (RBMXL3), mRNA [NM_001145346]
EPHA5	-2,62844	6,71E-04	Homo sapiens EPH receptor A5 (EPHA5), transcript variant 1, mRNA [NM_004439]
YWHAZ	-1,3594	6,73E-04	Homo sapiens tyrosine 3-monooxygenase/tryptophan 5-monooxygenase activation protein, mRNA [NM_145690]
MSL3	-3,81758	6,74E-04	Homo sapiens male-specific lethal 3 homolog (Drosophila) (MSL3), transcript variant 4, mRNA [NM_078628]
MCTP1	-3,79149	6,76E-04	Homo sapiens multiple C2 domains, transmembrane 1 (MCTP1), transcript variant L, mRNA [NM_024717]
RASSF10	-4,57661	6,76E-04	Homo sapiens Ras association (RalGDS/AF-6) domain family (N-terminal) member 10, mRNA [NM_001080521]
EIF1	-1,99218	6,77E-04	Homo sapiens eukaryotic translation initiation factor 1 (EIF1), mRNA [NM_005801]
PRKG1	-4,99855	6,84E-04	protein kinase, cGMP-dependent, type I [Source:HGNC Symbol;Acc:9414] [ENST00000373976]
ATP7A	-1,61795	6,86E-04	Homo sapiens ATPase, Cu++ transporting, alpha polypeptide (ATP7A), mRNA [NM_000052]
WDR88	-4,65903	6,87E-04	Homo sapiens WD repeat domain 88 (WDR88), mRNA [NM_173479]
LOC283332	-4,5803	6,87E-04	Homo sapiens uncharacterized LOC283332 (LOC283332), non-coding RNA [NR_026948]
CDK9	-1,25048	6,90E-04	Homo sapiens cyclin-dependent kinase 9 (CDK9), mRNA [NM_001261]
AR	-1,58975	6,91E-04	Homo sapiens androgen receptor (AR), transcript variant 1, mRNA [NM_000044]
FTL	-1,54151	6,91E-04	Homo sapiens ferritin, light polypeptide (FTL), mRNA [NM_000146]
PRB2	-3,78992	6,93E-04	Homo sapiens proline-rich protein BstNI subfamily 2 (PRB2), mRNA [NM_006248]
MYO3B	-4,5613	6,93E-04	Homo sapiens myosin IIIB (MYO3B), transcript variant 2, mRNA [NM_138995]
MGC45800	-3,80672	6,94E-04	Homo sapiens uncharacterized LOC90768 (MGC45800), non-coding RNA [NR_027107]
CCDC83	-3,57089	6,94E-04	Homo sapiens coiled-coil domain containing 83 (CCDC83), mRNA [NM_173556]
SCRT1	-3,65841	6,98E-04	Homo sapiens scratch homolog 1, zinc finger protein (Drosophila) (SCRT1), mRNA [NM_031309]
IFITM1	-2,00432	6,98E-04	Homo sapiens interferon induced transmembrane protein 1 (9-27) (IFITM1), mRNA [NM_003641]
HTR1D	-4,48375	6,98E-04	Homo sapiens 5-hydroxytryptamine (serotonin) receptor 1D (HTR1D), mRNA [NM_000864]
TLR10	-4,60123	6,99E-04	Homo sapiens toll-like receptor 10 (TLR10), transcript variant 1, mRNA [NM_030956]
FCRL3	-3,65533	7,01E-04	Homo sapiens Fc receptor-like 3 (FCRL3), mRNA [NM_052939]
PAGE2B	-4,72323	7,02E-04	Homo sapiens P antigen family, member 2B (PAGE2B), mRNA [NM_001015038]
C17orf57	-4,51848	7,02E-04	Homo sapiens chromosome 17 open reading frame 57 (C17orf57), transcript variant A, mRNA [NM_152347]

FLJ23865	-3,8481	7,02E-04	PREDICTED: Homo sapiens hypothetical protein FLJ23865 (FLJ23865), miscRNA [XR_109717]
MTUS1	-4,99589	7,03E-04	Homo sapiens microtubule associated tumor suppressor 1 (MTUS1), transcript variant 2, mRNA [NM_001001925]
MMP20	-3,72392	7,04E-04	Homo sapiens matrix metallopeptidase 20 (MMP20), mRNA [NM_004771]
ABI3BP	-2,00426	7,06E-04	Homo sapiens mRNA; cDNA DKFZp667H216 (from clone DKFZp667H216), [AL833204]
LOC414300	-3,92433	7,08E-04	Homo sapiens serine PI Kazal type 5-like 1, mRNA (cDNA clone IMAGE:4826141), [BC032033]
INF2	-1,20242	7,09E-04	Homo sapiens inverted formin, FH2 and WH2 domain containing (INF2), mRNA [NM_001031714]
GALNT13	-4,71249	7,12E-04	Homo sapiens [Source:HGNC Symbol;Acc:23242] [ENST00000409237]
ST13	-4,66688	7,13E-04	Homo sapiens suppression of tumorigenicity 13 (colon carcinoma) (Hsp70 interacting protein), mRNA [NM_003932]
CEP57	1,111334	7,16E-04	Homo sapiens centrosomal protein 57kDa (CEP57), transcript variant 1, mRNA [NM_014679]
PTPRC	-4,97322	7,17E-04	Homo sapiens protein tyrosine phosphatase, receptor type, C (PTPRC), transcript variant 4, mRNA [NM_080923]
PLIN4	-1,30356	7,17E-04	Homo sapiens perilipin 4 (PLIN4), mRNA [NM_001080400]
SSPN	-1,29616	7,18E-04	Homo sapiens sarcospan (Kras oncogene-associated gene) (SSPN), transcript variant 1, mRNA [NM_005086]
C18orf63	-4,49968	7,23E-04	Homo sapiens chromosome 18 open reading frame 63 (C18orf63), mRNA [NM_001174123]
SYT4	-4,13056	7,24E-04	Homo sapiens synaptotagmin IV (SYT4), mRNA [NM_020783]
ZHX3	-1,80139	7,24E-04	Homo sapiens zinc fingers and homeoboxes 3 (ZHX3), mRNA [NM_015035]
IL13	-4,2772	7,25E-04	Homo sapiens interleukin 13 (IL13), mRNA [NM_002188]
RRP9	1,273498	7,26E-04	Homo sapiens ribosomal RNA processing 9, homolog (yeast), mRNA [NM_004704]
KIAA1383	-4,51417	7,27E-04	Homo sapiens KIAA1383 (KIAA1383), mRNA [NM_019090]
GBA2	-1,34699	7,28E-04	Homo sapiens glucosidase, beta (bile acid) 2 (GBA2), mRNA [NM_020944]
TAAR3	-4,52486	7,30E-04	Homo sapiens trace amine associated receptor 3 (gene/pseudogene) (TAAR3), non-coding RNA [NR_028511]
SHARPIN	-1,19271	7,30E-04	Homo sapiens SHANK-associated RH domain interactor (SHARPIN), transcript variant 1, mRNA [NM_030974]
FAM19A4	-4,50709	7,32E-04	Homo sapiens family with sequence similarity 19 (chemokine (C-C motif)-like), member A4 , mRNA [NM_182522]
EEF1D	1,412653	7,32E-04	Homo sapiens eukaryotic translation elongation factor 1 delta, mRNA [NM_032378]
PTP4A2	-1,27438	7,32E-04	Homo sapiens protein tyrosine phosphatase type IVA, member 2 (PTP4A2), mRNA [NM_080391]
IFNK	-4,49109	7,33E-04	Homo sapiens interferon, kappa (IFNK), mRNA [NM_020124]
TUBB1	-3,18584	7,34E-04	Homo sapiens tubulin, beta 1 class VI (TUBB1), mRNA [NM_030773]
FGF5	-4,89506	7,36E-04	Homo sapiens fibroblast growth factor 5 (FGF5), transcript variant 1, mRNA [NM_004464]
C9orf85	1,105246	7,37E-04	Homo sapiens chromosome 9 open reading frame 85 (C9orf85), mRNA [NM_182505]
TRIOBP	-4,6141	7,38E-04	Homo sapiens TRIO and F-actin binding protein (TRIOBP), transcript variant 2, mRNA [NM_138632]
LOC283692	-4,54315	7,38E-04	Homo sapiens, clone IMAGE:5176336, mRNA, [BC035360]

SRRD	1,217807	7,40E-04	Homo sapiens SRR1 domain containing (SRRD), mRNA [NM_001013694]
CSTT	-4,63767	7,41E-04	Homo sapiens cystatin pseudogene (CSTT), non-coding RNA [NR_001279]
SNAI1	-1,4558	7,43E-04	Homo sapiens snail homolog 1 (Drosophila) (SNAI1), mRNA [NM_005985]
TCERG1	-1,17474	7,47E-04	Homo sapiens transcription elongation regulator 1 (TCERG1), transcript variant 1, mRNA [NM_006706]
LOC400891	-4,95951	7,49E-04	Homo sapiens chromosome 14 open reading frame 166B pseudogene (LOC400891), non-coding RNA [NR_027006]
HTR2C	-3,94248	7,50E-04	Homo sapiens 5-hydroxytryptamine (serotonin) receptor 2C (HTR2C), mRNA [NM_000868]
SCN9A	-4,44314	7,52E-04	Homo sapiens sodium channel, voltage-gated, type IX, alpha subunit (SCN9A), mRNA [NM_002977]
ABCC6	-2,22855	7,53E-04	Homo sapiens ATP-binding cassette, sub-family C (CFTR/MRP), member 6 (ABCC6), mRNA [NM_001079528]
TBC1D8B	-4,38599	7,55E-04	Homo sapiens TBC1 domain family, member 8B (with GRAM domain) (TBC1D8B), mRNA [NM_017752]
LOC285778	-4,82673	7,57E-04	Homo sapiens cDNA FLJ37615 fis, clone BRCOC2011996, [AK094934]
CT45A1	-4,31976	7,61E-04	Homo sapiens cancer/testis antigen family 45, member A1 (CT45A1), mRNA [NM_001017417]
ZNF367	-4,58438	7,63E-04	Homo sapiens zinc finger protein 367 (ZNF367), mRNA [NM_153695]
GCC2	-1,49386	7,68E-04	Homo sapiens GRIP and coiled-coil domain containing 2 (GCC2), transcript variant 1, mRNA [NM_181453]
TNFSF18	-3,55299	7,68E-04	Homo sapiens tumor necrosis factor (ligand) superfamily, member 18 (TNFSF18), mRNA [NM_005092]
AGXT2L2	-4,52443	7,69E-04	alanine-glyoxylate aminotransferase 2-like 2 [Source:HGNC Symbol;Acc:28249] [ENST00000476487]
ANO8	-4,85246	7,70E-04	Homo sapiens anoctamin 8 (ANO8), mRNA [NM_020959]
LGSN	-4,60068	7,71E-04	Homo sapiens lengsin, lens protein with glutamine synthetase domain (LGSN), mRNA [NM_016571]
FAM41AY1	-4,50989	7,71E-04	Homo sapiens family with sequence similarity 41, member A, Y-linked 1 (FAM41AY1), non-coding RNA [NR_028083]
LOC100133311	-3,93873	7,80E-04	Homo sapiens uncharacterized LOC100133311 (LOC100133311), transcript variant 1, non-coding RNA [NR_038831]
CUBN	-4,40691	7,81E-04	Homo sapiens cubilin (intrinsic factor-cobalamin receptor) (CUBN), mRNA [NM_001081]
PLAT	-1,3666	7,82E-04	Homo sapiens plasminogen activator, tissue (PLAT), transcript variant 1, mRNA [NM_000930]
C2orf90	-3,95738	7,84E-04	Homo sapiens cDNA FLJ46162 fis, clone TESTI4002520, [AK128042]
H1F0	1,49538	7,88E-04	Homo sapiens H1 histone family, member 0 (H1F0), mRNA [NM_005318]
POGZ	-1,35571	7,89E-04	Homo sapiens pogo transposable element with ZNF domain (POGZ), transcript variant 1, mRNA [NM_015100]
LOC100130468	-3,69814	7,91E-04	Homo sapiens cDNA FLJ44564 fis, clone UTERU3009259, [AK126528]
TFAM	-1,42806	7,92E-04	Homo sapiens transcription factor A, mitochondrial (TFAM), mRNA [NM_003201]
MFSD7	1,19282	7,92E-04	Homo sapiens major facilitator superfamily domain containing 7 (MFSD7), mRNA [NM_032219]
AKAP5	-4,69421	7,92E-04	Homo sapiens A kinase (PRKA) anchor protein 5 (AKAP5), mRNA [NM_004857]
TGS1	-1,31079	7,93E-04	Homo sapiens trimethylguanosine synthase 1 (TGS1), mRNA [NM_024831]
HAS2	-1,28651	7,94E-04	Homo sapiens hyaluronan synthase 2 (HAS2), mRNA [NM_005328]

TAS2R45	-3,44306	7,95E-04	Homo sapiens taste receptor, type 2, member 45 (TAS2R45), mRNA [NM_176886]
TMEM26	-3,73656	7,97E-04	Homo sapiens transmembrane protein 26 (TMEM26), mRNA [NM_178505]
NNT	-4,84965	7,97E-04	nicotinamide nucleotide transhydrogenase [Source:HGNC Symbol;Acc:7863] [ENST00000264663]
ITGB4	-1,33534	7,97E-04	Homo sapiens integrin, beta 4 (ITGB4), transcript variant 1, mRNA [NM_000213]
CSN3	-4,77532	7,99E-04	Homo sapiens casein kappa (CSN3), mRNA [NM_005212]
SCRN1	-4,66504	7,99E-04	secernin 1 [Source:HGNC Symbol;Acc:22192] [ENST00000409570]
B4GALT6	-4,62503	8,01E-04	Homo sapiens UDP-Gal:betaGlcNAc beta 1,4- galactosyltransferase, polypeptide 6 (B4GALT6), mRNA [NM_004775]
BCL3	-1,26339	8,02E-04	Homo sapiens B-cell CLL/lymphoma 3 (BCL3), mRNA [NM_005178]
EYS	-4,45248	8,03E-04	Homo sapiens eyes shut homolog (Drosophila) (EYS), transcript variant 1, mRNA [NM_001142800]
GLIPR1L1	-4,67957	8,03E-04	Homo sapiens GLI pathogenesis-related 1 like 1 (GLIPR1L1), mRNA [NM_152779]
HPGD	-4,6517	8,05E-04	Homo sapiens hydroxyprostaglandin dehydrogenase 15-(NAD) (HPGD), transcript variant 1, mRNA [NM_000860]
OR5K4	-4,44538	8,07E-04	Homo sapiens olfactory receptor, family 5, subfamily K, member 4 (OR5K4), mRNA [NM_001005517]
RBM39	1,220276	8,07E-04	Homo sapiens RNA binding motif protein 39 (RBM39), transcript variant 1, mRNA [NM_184234]
FAM155A	-1,33676	8,10E-04	Homo sapiens family with sequence similarity 155, member A (FAM155A), mRNA [NM_001080396]
STAG2	-1,50656	8,11E-04	Homo sapiens stromal antigen 2 (STAG2), transcript variant 1, mRNA [NM_001042749]
LOC148709	1,154619	8,12E-04	Homo sapiens actin pseudogene (LOC148709), non-coding RNA [NR_002929]
PDCD10	1,222188	8,12E-04	Homo sapiens programmed cell death 10 (PDCD10), transcript variant 1, mRNA [NM_007217]
RTL1	-3,77835	8,14E-04	Homo sapiens retrotransposon-like 1 (RTL1), mRNA [NM_001134888]
GPNMB	-4,6412	8,14E-04	Homo sapiens glycoprotein (transmembrane) nmb, mRNA (cDNA clone IMAGE:3345861), complete cds, [BC011595]
SLCO1C1	-3,56652	8,15E-04	Homo sapiens solute carrier organic anion transporter family, member 1C1 (SLCO1C1), mRNA [NM_017435]
RSBN1	-1,2114	8,15E-04	Homo sapiens round spermatid basic protein 1 (RSBN1), mRNA [NM_018364]
LOXHD1	-2,57155	8,15E-04	lipoxygenase homology domains 1 [Source:HGNC Symbol;Acc:26521] [ENST00000335730]
LOC729013	-1,17078	8,17E-04	Homo sapiens uncharacterized LOC729013 (LOC729013), non-coding RNA [NR_034137]
CYP19A1	-3,78882	8,18E-04	Homo sapiens cytochrome P450, family 19, subfamily A, polypeptide 1, mRNA, [BC035714]
LDHA	-1,42314	8,19E-04	Homo sapiens lactate dehydrogenase A (LDHA), transcript variant 1, mRNA [NM_005566]
RECQL	-4,50367	8,21E-04	Homo sapiens RecQ protein-like (DNA helicase Q1-like) (RECQL), transcript variant 1, mRNA [NM_002907]
GCHFR	1,23691	8,29E-04	Homo sapiens GTP cyclohydrolase I feedback regulator (GCHFR), mRNA [NM_005258]
CASK	-4,30338	8,29E-04	calcium/calmodulin-dependent serine protein kinase (MAGUK family) [Source:HGNC Symbol;Acc:1497] [ENST00000378154]
OR4N3P	-4,88785	8,30E-04	Homo sapiens olfactory receptor, family 4, subfamily N, member 3 pseudogene (OR4N3P), [NR_028067]
FLJ46320	-4,62408	8,32E-04	Homo sapiens cDNA FLJ46320 fis, clone TESTI4042846, [AK128193]

ZNF254	-1,17329	8,34E-04	Homo sapiens zinc finger protein 254 (ZNF254), mRNA [NM_203282]
GPATCH1	-1,18891	8,34E-04	Homo sapiens G patch domain containing 1 (GPATCH1), mRNA [NM_018025]
CTLA4	-4,55059	8,35E-04	Homo sapiens cytotoxic T-lymphocyte-associated protein 4 (CTLA4), transcript variant 1, mRNA [NM_005214]
LINC00320	-4,65461	8,37E-04	Homo sapiens long intergenic non-protein coding RNA 320 (LINC00320), non-coding RNA [NR_024090]
FUT9	-4,63617	8,40E-04	Homo sapiens fucosyltransferase 9 (alpha (1,3) fucosyltransferase) (FUT9), mRNA [NM_006581]
IFLTD1	-4,95475	8,40E-04	Homo sapiens intermediate filament tail domain containing 1 (IFLTD1), transcript variant 2, mRNA [NM_152590]
P2RY1	-4,50161	8,41E-04	Homo sapiens purinergic receptor P2Y, G-protein coupled, 1 (P2RY1), mRNA [NM_002563]
F5	-3,58842	8,42E-04	Homo sapiens coagulation factor V (proaccelerin, labile factor) (F5), mRNA [NM_000130]
ATP13A5	-4,56958	8,44E-04	Homo sapiens ATPase type 13A5 (ATP13A5), mRNA [NM_198505]
LOC285965	-4,61244	8,45E-04	Homo sapiens uncharacterized LOC285965 (LOC285965), non-coding RNA [NR_033897]
LOC649133	-3,45577	8,47E-04	Homo sapiens uncharacterized LOC649133 (LOC649133), non-coding RNA [NR_038829]
ST8SIA4	-4,9428	8,48E-04	Homo sapiens ST8 alpha-N-acetyl-neuraminide alpha-2,8-sialyltransferase 4 (ST8SIA4), mRNA [NM_175052]
CALCRL	-4,68323	8,48E-04	Homo sapiens calcitonin receptor-like (CALCRL), mRNA [NM_005795]
ZWINT	1,331279	8,50E-04	Homo sapiens ZW10 interactor (ZWINT), transcript variant 2, mRNA [NM_032997]
DDX50	-3,45972	8,50E-04	Homo sapiens DEAD (Asp-Glu-Ala-Asp) box polypeptide 50 (DDX50), mRNA [NM_024045]
LOC100129399	-3,63845	8,53E-04	Homo sapiens cDNA FLJ42017 fis, clone SPLEN2033153, [AK124011]
RAB7B	1,176352	8,54E-04	Homo sapiens RAB7B, member RAS oncogene family (RAB7B), transcript variant 1, mRNA [NM_177403]
DNAH12	-4,50056	8,55E-04	Homo sapiens dynein, axonemal, heavy chain 12 (DNAH12), transcript variant 1, mRNA [NM_178504]
KRTAP4-3	-4,49195	8,55E-04	Homo sapiens keratin associated protein 4-3 (KRTAP4-3), mRNA [NM_033187]
GLRB	-4,92862	8,57E-04	Homo sapiens glycine receptor, beta (GLRB), transcript variant 1, mRNA [NM_000824]
LOC647012	-4,88491	8,57E-04	Homo sapiens YY1 transcription factor pseudogene (LOC647012), non-coding RNA [NR_033658]
AGAP7	-3,97221	8,57E-04	Homo sapiens ArfGAP with GTPase domain, ankyrin repeat and PH domain 7 (AGAP7), mRNA [NM_001077685]
GLYCAM1	-4,49991	8,60E-04	Homo sapiens glycosylation dependent cell adhesion molecule 1 (pseudogene) (GLYCAM1),[NR_003039]
PIWIL1	-3,60297	8,61E-04	Homo sapiens piwi-like 1 (Drosophila) (PIWIL1), transcript variant 1, mRNA [NM_004764]
CLEC9A	-4,4986	8,61E-04	Homo sapiens C-type lectin domain family 9, member A (CLEC9A), mRNA [NM_207345]
STEAP2	-3,58238	8,63E-04	six transmembrane epithelial antigen of the prostate 2 [Source:HGNC Symbol;Acc:17885] [ENST00000402625]
ATF4	-1,45637	8,65E-04	Homo sapiens activating transcription factor 4 (tax-responsive enhancer element B67) (ATF4), mRNA [NM_001675]
PMVK	1,392821	8,74E-04	Homo sapiens phosphomevalonate kinase (PMVK), mRNA [NM_006556]
ANTXRL	-3,95264	8,76E-04	Homo sapiens anthrax toxin receptor-like (ANTXRL), non-coding RNA [NR_003601]
ADAMDEC1	-4,20823	8,77E-04	Homo sapiens ADAM-like, decysin 1 (ADAMDEC1), transcript variant 2, mRNA [NM_001145271]
FBXW7	-1,33436	8,78E-04	Homo sapiens F-box and WD repeat domain containing 7 (FBXW7), transcript variant 1, mRNA [NM_033632]

FBXO47	-3,91465	8,79E-04	Homo sapiens F-box protein 47 (FBXO47), mRNA [NM_001008777]
SKOR2	-4,92275	8,83E-04	SKI family transcriptional corepressor 2 [Source:HGNC Symbol;Acc:32695] [ENST00000425639]
C3orf79	-4,41129	8,87E-04	Homo sapiens chromosome 3 open reading frame 79 (C3orf79), mRNA [NM_001101337]
LOC339593	-4,47635	8,90E-04	Homo sapiens uncharacterized LOC339593 (LOC339593), non-coding RNA [NR_038972]
GMCL1P1	-4,49552	8,92E-04	Homo sapiens germ cell-less homolog 1 (Drosophila) pseudogene 1 (GMCL1P1), non-coding RNA [NR_003281]
EDN3	-4,83613	8,92E-04	Homo sapiens endothelin 3 (EDN3), transcript variant 2, mRNA [NM_207032]
GABRB2	-4,69834	8,92E-04	Homo sapiens gamma-aminobutyric acid (GABA) A receptor, beta 2 (GABRB2), mRNA [NM_021911]
AGTR2	-4,7136	9,01E-04	Homo sapiens angiotensin II receptor, type 2 (AGTR2), mRNA [NM_000686]
AFF1	-4,69205	9,01E-04	AF4/FMR2 family, member 1 [Source:HGNC Symbol;Acc:7135] [ENST00000504956]
CCDC73	-4,48576	9,01E-04	Homo sapiens coiled-coil domain containing 73 (CCDC73), mRNA [NM_001008391]
LOC100130197	-4,49476	9,03E-04	Homo sapiens uncharacterized LOC100130197 (LOC100130197), transcript variant 1, non-coding RNA [NR_038827]
C10orf40	-4,20924	9,06E-04	Homo sapiens chromosome 10 open reading frame 40 (C10orf40), non-coding RNA [NR_024340]
PLEKHA1	-1,49203	9,06E-04	Homo sapiens pleckstrin homology domain containing, family A member 1, mRNA [NM_001001974]
LOC646719	-4,05205	9,07E-04	PREDICTED: Homo sapiens hypothetical LOC646719 (LOC646719), miscRNA [XR_112726]
LOC100505876	-4,13837	9,07E-04	Homo sapiens uncharacterized LOC100505876 (LOC100505876), transcript variant 1, non-coding RNA [NR_037879]
LHX5	-4,38253	9,11E-04	Homo sapiens LIM homeobox 5 (LHX5), mRNA [NM_022363]
FLJ46120	-3,615	9,11E-04	Homo sapiens cDNA FLJ46120 fis, clone TESTI2038733, [AK128002]
MLL2	-1,50127	9,13E-04	Homo sapiens myeloid/lymphoid or mixed-lineage leukemia 2 (MLL2), mRNA [NM_003482]
ENTHD1	-4,81685	9,13E-04	Homo sapiens ENTH domain containing 1 (ENTHD1), mRNA [NM_152512]
P2RY14	-4,63912	9,14E-04	Homo sapiens purinergic receptor P2Y, G-protein coupled, 14 (P2RY14), transcript variant 2, mRNA [NM_014879]
CFHR2	-3,77142	9,14E-04	Homo sapiens complement factor H-related 2 (CFHR2), mRNA [NM_005666]
SYTL4	-3,36707	9,25E-04	synaptotagmin-like 4 [Source:HGNC Symbol;Acc:15588] [ENST00000372981]
PSAPL1	-3,6543	9,27E-04	Homo sapiens prosaposin-like 1 (gene/pseudogene) (PSAPL1), mRNA [NM_001085382]
ZHX3	-1,20173	9,30E-04	Homo sapiens zinc fingers and homeoboxes 3 (ZHX3), mRNA [NM_015035]
NTRK2	-3,58664	9,37E-04	Homo sapiens neurotrophic tyrosine kinase, receptor, type 2 (NTRK2), transcript variant d, mRNA [NM_001018065]
KLLN	-1,30417	9,40E-04	Homo sapiens killin, p53-regulated DNA replication inhibitor (KLLN), mRNA [NM_001126049]
BPIFB1	-3,60986	9,40E-04	Homo sapiens BPI fold containing family B, member 1 (BPIFB1), mRNA [NM_033197]
PKD1L1	-4,60072	9,41E-04	Homo sapiens polycystic kidney disease 1 like 1 (PKD1L1), mRNA [NM_138295]
OR2J3	-1,96421	9,41E-04	Homo sapiens olfactory receptor, family 2, subfamily J, member 3 (OR2J3), mRNA [NM_001005216]
NFE2L2	-4,6532	9,46E-04	Homo sapiens nuclear factor (erythroid-derived 2)-like 2 (NFE2L2), transcript variant 1, mRNA [NM_006164]

CRP	-3,6861	9,54E-04	Homo sapiens C-reactive protein, pentraxin-related (CRP), mRNA [NM_000567]
LPGAT1	-1,23862	9,54E-04	Homo sapiens lysophosphatidylglycerol acyltransferase 1 (LPGAT1), mRNA [NM_014873]
GRN	-1,66727	9,54E-04	Homo sapiens granulin (GRN), mRNA [NM_002087]
TPCN1	-1,25625	9,55E-04	Homo sapiens two pore segment channel 1 (TPCN1), transcript variant 2, mRNA [NM_017901]
COL1A2	-1,66709	9,60E-04	Homo sapiens collagen, type I, alpha 2 (COL1A2), mRNA [NM_000089]
LOC100133311	-4,49459	9,60E-04	Homo sapiens uncharacterized LOC100133311 (LOC100133311), transcript variant 1, non-coding RNA [NR_038831]
DCTN3	1,292679	9,65E-04	Homo sapiens dynactin 3 (p22) (DCTN3), transcript variant 1, mRNA [NM_007234]
FLJ26086	-3,83578	9,67E-04	Homo sapiens cDNA FLJ26086 fis, clone RCT05358, [AK129597]
PYHIN1	-4,60929	9,67E-04	Homo sapiens pyrin and HIN domain family, member 1 (PYHIN1), transcript variant 4, mRNA [NM_198930]
BOC	-1,3297	9,67E-04	Homo sapiens Boc homolog (mouse) (BOC), mRNA [NM_033254]
AKAP6	-4,59657	9,73E-04	Homo sapiens A kinase (PRKA) anchor protein 6 (AKAP6), mRNA [NM_004274]
FAM201A	-4,88599	9,77E-04	Homo sapiens family with sequence similarity 201, member A (FAM201A), non-coding RNA [NR_027294]
WDR69	-3,75582	9,77E-04	Homo sapiens WD repeat domain 69 (WDR69), mRNA [NM_178821]
TPTE2P3	-4,5992	9,80E-04	Homo sapiens transmembrane phosphoinositide 3-phosphatase and tensin homolog 2 pseudogene 3, [NR_002793]
KLK12	-4,40179	9,84E-04	Homo sapiens kallikrein-related peptidase 12 (KLK12), transcript variant 2, mRNA [NM_145894]
KIAA0825	-4,87344	9,88E-04	Homo sapiens KIAA0825 (KIAA0825), transcript variant 2, mRNA [NM_173665]
PVRL3	-1,30568	9,88E-04	Homo sapiens poliovirus receptor-related 3 (PVRL3), transcript variant 1, mRNA [NM_015480]
MARVELD3	-4,45876	9,90E-04	Homo sapiens MARVEL domain containing 3 (MARVELD3), transcript variant 1, mRNA [NM_001017967]
KIAA0090	-1,0346	9,90E-04	Homo sapiens KIAA0090 (KIAA0090), mRNA [NM_015047]
MRPS17	-1,21351	9,90E-04	Homo sapiens mitochondrial ribosomal protein S17 (MRPS17), mRNA [NM_015969]
NRXN1	-4,64234	9,94E-04	Homo sapiens neurexin 1 (NRXN1), transcript variant alpha2, mRNA [NM_001135659]
POF1B	-4,24861	9,97E-04	Homo sapiens premature ovarian failure, 1B (POF1B), mRNA [NM_024921]
PKD2L2	-3,57896	1,00E-03	polycystic kidney disease 2-like 2 [Source:HGNC Symbol;Acc:9012] [ENST00000350250]

Table S3. Nucleotide primer sequences used to amplify *SMC1B* gene.

**Exons**	**Forward primer sequence (5'-3')**	**Reverse primer sequence (5'-3')**	**Size (bp)**
1	TCCGCGGGCGCTTGATAACG	CAGACGCCCACACCCCACAG	241
2	TTCACTTCGATTGGTGGCCT	AGGTTCTGTTACTTGCTTCTG	440
3-4	GGTGGATTTTGAGGTATATCTG	CCAAGCCCATACCTAGTGAT	547
5	GAAACC CTCGCCAAATTATGC	CCACACCTGGCCTATACTA	555
6	ACCTTGCTCAACCTTTGGCA	CCCCACAATTTACATTGAGG	540
7	GGGAAGGTGG TCGATTATGA	GGAGATTCATTCTGGAGACAG	370
8	GATTCAGGAGTTGAACCCTG	TTGAGGCTACCGTAAGCCAT	373
9	TCTATTCCCCTGCAATGCCA	GTTCATGTCAAGCTTCTCCC	543
10	TTGGTAAGGC TAGTCCCTCT	CCTAACTGGCATCCAAGCTA	538
11	GATCTTTTGCTG CCATGCGT	CACTGAAGCCATAACCACCT	442
12	CCTAGA ATTGCTTTGG GCCT	CTGGAAGCTGTGTATCAAACTG	571
13	GTGCAGGTGTGGTG GTAT	TGACAGAGTGAGAGACTCTG	383
14	AG ATTACAGCCACCATGCCT	GGCACTGTTACCTATCAACC	451
15	GGTG GGATTAGTTT TTGGCC	CGGCCTAGCTGTTTCTTGAT	336
16	AGTTGGGTGAC AGCTACTTGA	TCACTAACAGGACCCTTCAG	374
17	CTCGGGATAACTGGTTTGGT	AGACCAAGAGCAACAAAGGG	359
18	GAA CTTACAGCACTCACAGG	GGGGCTACAGAACAAGAGAA	368
19	TTGGAGTGCCATGAACCACT	GTGTGGCAGAGACCCACAATT	364
20	AG AGAAGGAGTGGATGCACA	GACAACAGAGGGGAGATTGT	360
21	AAAGAACCTCCTCAGTGGTC	TGTGGCAGTGGTTATTTCCC	312
22	CCTGGGTATG TTAGGAGAGT	ATCTTCTGTAGGAAGGTCCC	436
23	GGTCACAT ACATGTGGACTG	GCCTAGACCAAGGACTGATT	352
24	CTTGTC ACCTAAACTG ACC	GTCTAGGAAAGCACCAGTAG	380
25	GACTCA CTAATGGCTC TTAC	TTGCTCCAGAAGTCTCCTGT	265

Table S4. Polymorphisms identified in *SMC1B* gene.

Exon	Polymorphism	Amino acid change
5	c.702G>A	p.K234K
5	c.854+75A>G	
6	c.1047T>A	p.S349R
9	c.1417T>G	p.F473V
10	c.1887A>C	p.A629A
10	c.1731+30A>G	
11	c.1755A>G	p.L585L
14	c.2209T>C	p.L737L
14	c.2243G>A	p.C748Y
14	c.2273G>A	p.R758Q
14	c.2251A>C	p.L751L
14	c.2295T>C	p.F765F
12	c.2058+12T>C	
16	c.2562+7G>A	
20	c.3037T>C	p.L1013L
21	c.3148C>T	p.L1050L
21	c.3165C>G	p.F1055L
22	c.3417T>C	p.A1139A
25	c.3708+36G>C	

Table S5. Primer sequences used for RT-qPCR in human fibroblasts and mouse tissues.

Gene	Forward primer sequence (5’-3’)	Reverse primer sequence (5’-3’)
SMC1B	GGCCTCAGTACTTAAAGCC	AAATCAGCCAGCTCTGTCTC
HPRT	AGCCAGACTTTGTTGGATTTG	TACTAAGCAGATGGCCACAGA

Gene	Forward primer sequence (5’-3’)	Reverse primer sequence (5’-3’)
Smc1b	GGACGGGAATTTAAGTGTTG	CATGCTCTATCCAAATCAGC
Actin	AATCGTGCGTGACATCAAAG	AAGGAAGGCTGGAAAAGAGC

Table S6. Primers sequences used for RT-qPCR for validating dysregulated genes after siRNA against *SMC1B*

**Gene**	**Forward primer sequence (5'-3')**	**Reverse primer sequence (5'-3')**
HBB	GAAGGCTCATGGCAAGAAAG	CACTGGTGGGGTGAATTCTT
PCDHA1	CTGATGCAGACATTGGTGCT	CCCATCAGTGGCAGTCAGTA
COL11A1	TTGGTGTTGAGGTTGGGAGA	ATTGCTACCCGATGCCACTT
PROM1	AAACTAGCCTGCGGTCATCT	GTCAAGTTCTGCATCCACGG
VEGFA	ATCCAATCGAGACCCTGGTG	ATCTCTCCTATGTGCTGGCC
GLDN	TCCCCACAAGCAGAATCCAT	TGTAAACAACGTGCCCACAG
PCDHB5	AAAATCCCAGAGAGCACCCA	TCTGCCATCTCCGCGATTAT
PCDHB6	CACGATTTAGACACCGGCAG	CCAACGGTTTGTCTAGCACC
PCDHB19P	CCCATCCGCGAAAATTCTCC	TTCGGCATGAACGGAACATC
HOXD3	CAGCCTCCTGGTCTGAACTC	ATCCAGGGGAAGATCTGCTT
HOXD12	CGCTTCCCCCTATCTCCTAC	TTCGGGCGCATAGAACTTAG
HOXB2	T TTTAGCCGTTCGCTTAGAGG	CGGATAGCTGGAGACAGGAG
HOXB7	TAATGCTGTCTTTGTGGACTGT	AACACGCGAGTGGTAGGTTT

Table S7. Primers used for validation of SMC1B binding sites by RT-qPCR.

Primer	Forward primer sequence (5'-3')	Reverse primer sequence (5'-3')	coordinates
HOXD3	TATCAGGGTTCGGCTGCTTT	GAATACAGGGCCAAACGGTC	chr2:177030106+177030227
HOXD12	CTGTGCTCAAACGCTCTCTG	GGCCACTTACCAGTTCCTCT	chr2:176961944+176962073
HOXB2	CAGAGCCAAACATGGTGCTT	GCGTGCAAAGTTTGATTGGG	chr17:46624862+46624985
HOXB7	TTCGCATCCAGGGGTAGATC	TTCCTTCAACATGCACTGCG	chr17:46687887+46688032
PCDHB5	GGAAGCAACCAAGATGTCGT	TCCTCAATGTCTTTTCATGGGT	chr5:140512460+140512582
PCDHB6	TACAGGTACAGGTTGGGCAT	TTTTCTACTTGTGGCATCAGGT	chr5:140526845+140526944
PCDHB19P	ATGATGTTCCGTTCATGCCG	TGGCTGTGTTGGTGATGTTG	chr5:140620648+140620758
LMF1	TTCCTACAAAGACAACGGCG	CCGGAACAAGAAAGGACGTC	chr16:911700-911859
ELP4	AGCCAGACTTTGTTGGATTTG	TACTAAGCAGATGGCCACAGA	chr11:31531397-31531561
TIMELESS	GGATGATCTGCTTGCGTGTT	AAGGACGTGGAAGGTGAGAG	chr12:56815732-56815902
NEGATIVE1	TACAGGTACAGGTTGGGCAT	TTTTCTACTTGTGGCATCAGGT	chr5:140618351-140618463
NEGATIVE 2	CTGTCACTTCTTTGGCTGGG	ATTCTCTATTGGGGCTGGGG	chr11:8642689-8642807
NEGATIVE 3	CAACAGGACAGGGACCAAAT	CCAGGGCTCCTTCTAGCTTT	chr9:116184241-116184471

Supplementary Figure Legends

Supplementary Figure S1. Analysis of *Smc1b* transcript in mouse tissues. (**A**) testis. (**B**) brain. (**C**) spleen.

Supplementary Figure S2. SMC1B expression in nuclear mouse tiusses. (**A**) The expression of Smc1b in somatic cells was also confirmed by using a published antibody. (**B**) Immunoblot of nuclear and **(C)** cytosolic extracts from a variety of mouse tissues probed with a SMC1B antibody.

Supplementary Figure S3. (**A**) Samples from the flow-through deriving from experiments described in Figure 1C and (**B**) in Figure 1D were electrophoresed and visualized by Western blot using anti-SMC1A, SMC1B, SMC3 and RAD21 antibodies.

Supplementary Figure S4. Effects of *SMC1B* depletion in human primary fibroblasts. Flow cytofluorimetric analysis of cell-cycle phases in *SMC1B* downregulated cells.

Supplementary Figure S5. Physical interaction of ATM with SMC1A and SMC1B. SMC1B did not interact with ATM. Total protein extract from human primary fibroblasts irradiated with 10 Gy was co-IPed with an antibody anti-ATM 1 h after exposure. No signal was detected from SMC1B, whereas SMC1A was found to interact physically with ATM.

Supplementary Figure S6. ChIP-seq data validation by RT-qPCR. Each sample was run in duplicate and repeated at least three times. Corresponding primers of the selected genes are described in Table S7. Input was determined relative to three genomic regions (chr5:140618351-140618463; chr9:116184241-116184471; chr11:8642689-8642807) that did not bind SMC1B.

Supplementary Figure S7. Genomic-wide distribution of SMC1B. (**A**) Genomic binding of SMC1B at *HOXB7* locus on chromosome 17 and (**B**) *HOXD3* on chromosome 2.

A

Seq 158 testis = *Smc1b*

>158_TE~1 sequence exported from chromatogram file

GCTTAGTACACTGATAATATCGACACATTAAAAACCCAATCCAGAAGGGCAAAGAAGATATTGATAACCTAAAAAAGA
CCGAAGAAGAATGTCTGAAAATTGTTGAGGAACTCATGGTGAAGCAAGAGCAAATTAAGGAAGTGCTTGCCACACAG
AGTTCCAACATTGAAAAAATTCACATACAAATTGAAGAGGAACGCAAGAAGGTTTTGGCTGTTGATAGGGAAGTTGG
AAAATTACAGAAGGAAGTTGTAATCCTTCAGGGTTCTTTGGAACAGAAACTGCTAGAGAAACATAACTTGCTGCTAGA
TTGCAAAGTTCAAGACATTGACATAAGTCTTGTGCTGGGGTCATA

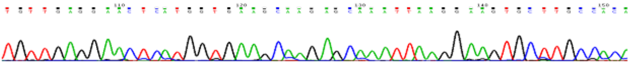

```
Smc1b          TATTCAGCTCGAATATAGTCGAAATCAGCTTAAGAAGAAACTGAATAATATCGACACATT
Seq158         ---------------------------GCTTAGTAC---ACTGA-TAATATCGACACATT
                                          *****  *    ***** ***************

Smc1b          AAAAACCACAATCCAGAAGGGCAAAGAAGATATTGATAACCTAAAAAAGACCGAAGAAGA
Seq158         AAAAACC-CAATCCAGAAGGGCAAAGAAGATATTGATAACCTAAAAAAGACCGAAGAAGA
               ******* ****************************************************

Smc1b          ATGTCTGAAAATTGTTGAGGAACTCATGGTGAAGCAAGAGCAAATTAAGGAAGTGCTTGC
Seq158         ATGTCTGAAAATTGTTGAGGAACTCATGGTGAAGCAAGAGCAAATTAAGGAAGTGCTTGC
               ************************************************************

Smc1b          CACACAGAGTTCCAACATTGAAAAAATTCACATACAAATTGAAGAGGAACGCAAGAAGGT
Seq158         CACACAGAGTTCCAACATTGAAAAAATTCACATACAAATTGAAGAGGAACGCAAGAAGGT
               ************************************************************

Smc1b          TTTGGCTGTTGATAGGGAAGTTGGAAAATTACAGAAGGAAGTTGTAATCATTCAGGGTTC
Seq158         TTTGGCTGTTGATAGGGAAGTTGGAAAATTACAGAAGGAAGTTGTAATCATTCAGGGTTC
               ************************************************************

Smc1b          TTTGGAACAGAAACTGCTAGAGAAACATAACTTGCTGCTAGATTGCAAAGTTCAAGACAT
Seq158         TTTGGAACAGAAACTGCTAGAGAAACATAACTTGCTGCTAGATTGCAAAGTTCAAGACAT
               ************************************************************

Smc1b          TGACATAAGTCTTGTGCTGGGGTCAT-
Seq158         TGACATAAGTCTTGTGCTGGGGTCATA
               **************************
```

B

Seq 160 brain = *Smc1b*

>160_CE~1 sequence exported from chromatogram file

GACTGATAATATCGACACATTAAAAACCCAATCCAGAAGGGCAAAGAAGATATTGATA
ACCTAAAAAAGACCGAAGAAGAATGTCTGAAAATTGTTGAGGAACTCATGGTGAAGC
AAGAGCAAATTAAGGAAGTGCTTGCCACACAGAGTTCCAACATTGAAAAAATTCACAT
ACAAATTGAAGAGGAACGCAAGAAGGTTTTGGCTGTTGATAGGGAAGTTGGAAAATTA
CAGAAGGAAGTTGTAATCATTCAGGGTTCTTTGGAACAGAAACTGCTAGAGAAACATA
ACTTGCTGCTAGATTGCAAAGTTCAAGACATTGACATAAGTCTTGTGCTGGGGTCATA

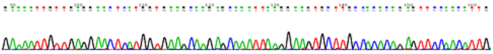

```
Smc1b          TATTCAGCTCGAATATAGTCGAAATCAGCTTAAGAAGAAACTGAATAATATCGACACATT
Seq160         --------------------------------------GACTGA-TAATATCGACACATT
                                                       ***** ***************

Smc1b          AAAAACCACAATCCAGAAGGGCAAAGAAGATATTGATAACCTAAAAAAGACCGAAGAAGA
Seq160         AAAAACCACAATCCAGAAGGGCAAAGAAGATATTGATAACCTAAAAAAGACCGAAGAAGA
               ************************************************************

Smc1b          ATGTCTGAAAATTGTTGAGGAACTCATGGTGAAGCAAGAGCAAATTAAGGAAGTGCTTGC
Seq160         ATGTCTGAAAATTGTTGAGGAACTCATGGTGAAGCAAGAGCAAATTAAGGAAGTGCTTGC
               ************************************************************

Smc1b          CACACAGAGTTCCAACATTGAAAAAATTCACATACAAATTGAAGAGGAACGCAAGAAGGT
Seq160         CACACAGAGTTCCAACATTGAAAAAATTCACATACAAATTGAAGAGGAACGCAAGAAGGT
               ************************************************************

Smc1b          TTTGGCTGTTGATAGGGAAGTTGGAAAATTACAGAAGGAAGTTGTAATCATTCAGGGTTC
Seq160         TTTGGCTGTTGATAGGGAAGTTGGAAAATTACAGAAGGAAGTTGTAATCATTCAGGGTTC
               ************************************************************

Smc1b          TTTGGAACAGAAACTGCTAGAGAAACATAACTTGCTGCTAGATTGCAAAGTTCAAGACAT
Seq160         TTTGGAACAGAAACTGCTAGAGAAACATAACTTGCTGCTAGATTGCAAAGTTCAAGACAT
               ************************************************************

Smc1b          TGACATAAGTCTTGTGCTGGGGTCAT-
Seq160         TGACATAAGTCTTGTGCTGGGGTCATA
               **************************
```

C

Seq 159 spleen = *Smc1b*

>159_MI~1 sequence exported from chromatogram file

AACCTGAATAATATCGACACATTAAAAACCACAATCCGAAGGGCAAAGAAGATATTG
ATAACCTAAAAAAGACCGAAGAAGAATGTCTGAAAATTGTTGAGGAACTCATGGTGA
AGCAAGAGCAAATTAAGGAAGTGCTTGCCACACAGAGTTCCAACATTGAAAAAATTCA
CATACAAATTGAAGAGGAACGCAAGAAGGTTTTGGCTGTTGATAGGGAAGTTGGAAA
ATTACAGAAGGAAGTTGTAATCATTCAGGGTTCTTTGGAACAGAAACTGCTAGAGAAA
CATAACTTGCTGCTAGATTGCAAAGTTCAAGACATTGACATAAGTCTTGTGCTGGGGTC
ATA

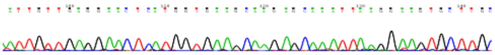

```
Smc1b          TATTCAGCTCGAATATAGTCGAAATCAGCTTAAGAAGAAACTGAATAATATCGACACATT
Seq159         -------------------------------------AACCTGAATAATATCGACACATT
                                                    ** ********************

Smc1b          AAAAACCACAATCCAGAAGGGCAAAGAAGATATTGATAACCTAAAAAAGACCGAAGAAGA
Seq159         AAAAACCACAATCCAGAAGGGCAAAGAAGATATTGATAACCTAAAAAAGACCGAAGAAGA
               ************************************************************

Smc1b          ATGTCTGAAAATTGTTGAGGAACTCATGGTGAAGCAAGAGCAAATTAAGGAAGTGCTTGC
Seq159         ATGTCTGAAAATTGTTGAGGAACTCATGGTGAAGCAAGAGCAAATTAAGGAAGTGCTTGC
               ************************************************************

Smc1b          CACACAGAGTTCCAACATTGAAAAAATTCACATACAAATTGAAGAGGAACGCAAGAAGGT
Seq159         CACACAGAGTTCCAACATTGAAAAAATTCACATACAAATTGAAGAGGAACGCAAGAAGGT
               ************************************************************

Smc1b          TTTGGCTGTTGATAGGGAAGTTGGAAAATTACAGAAGGAAGTTGTAATCATTCAGGGTTC
Seq159         TTTGGCTGTTGATAGGGAAGTTGGAAAATTACAGAAGGAAGTTGTAATCATTCAGGGTTC
               ************************************************************

Smc1b          TTTGGAACAGAAACTGCTAGAGAAACATAACTTGCTGCTAGATTGCAAAGTTCAAGACAT
Seq159         TTTGGAACAGAAACTGCTAGAGAAACATAACTTGCTGCTAGATTGCAAAGTTCAAGACAT
               ************************************************************

Smc1b          TGACATAAGTCTTGTGCTGGGGTCAT-
Seq159         TGACATAAGTCTTGTGCTGGGGTCATA
               **************************
```

Figure S1

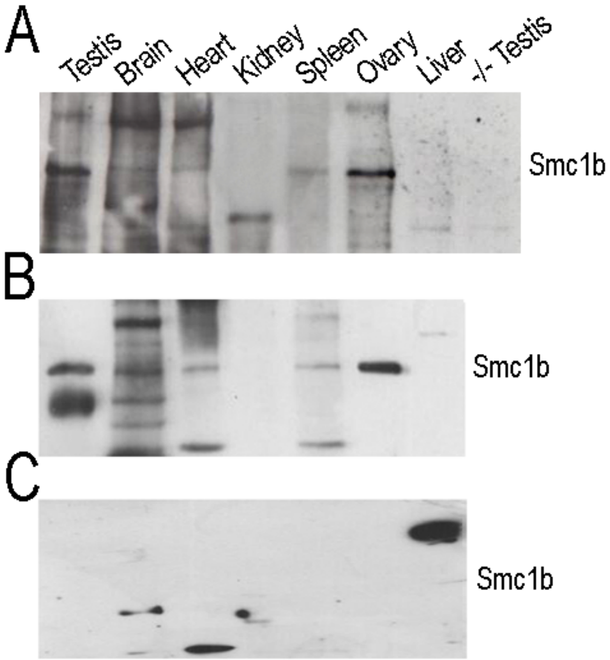
A
Testis
Brain
Heart
Kidney
Spleen
Ovary
Liver
-/- Testis
Smc1b
B
Smc1b
C
Smc1b

Figure S2

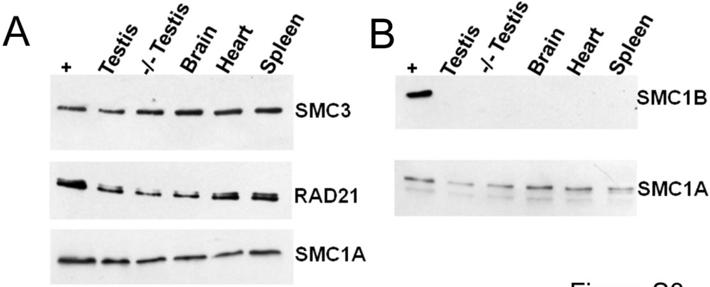
A
+
Testis
-/- Testis
Brain
Heart
Spleen
SMC3
RAD21
SMC1A
B
+
Testis
-/- Testis
Brain
Heart
Spleen
SMC1B
SMC1A

Figure S3

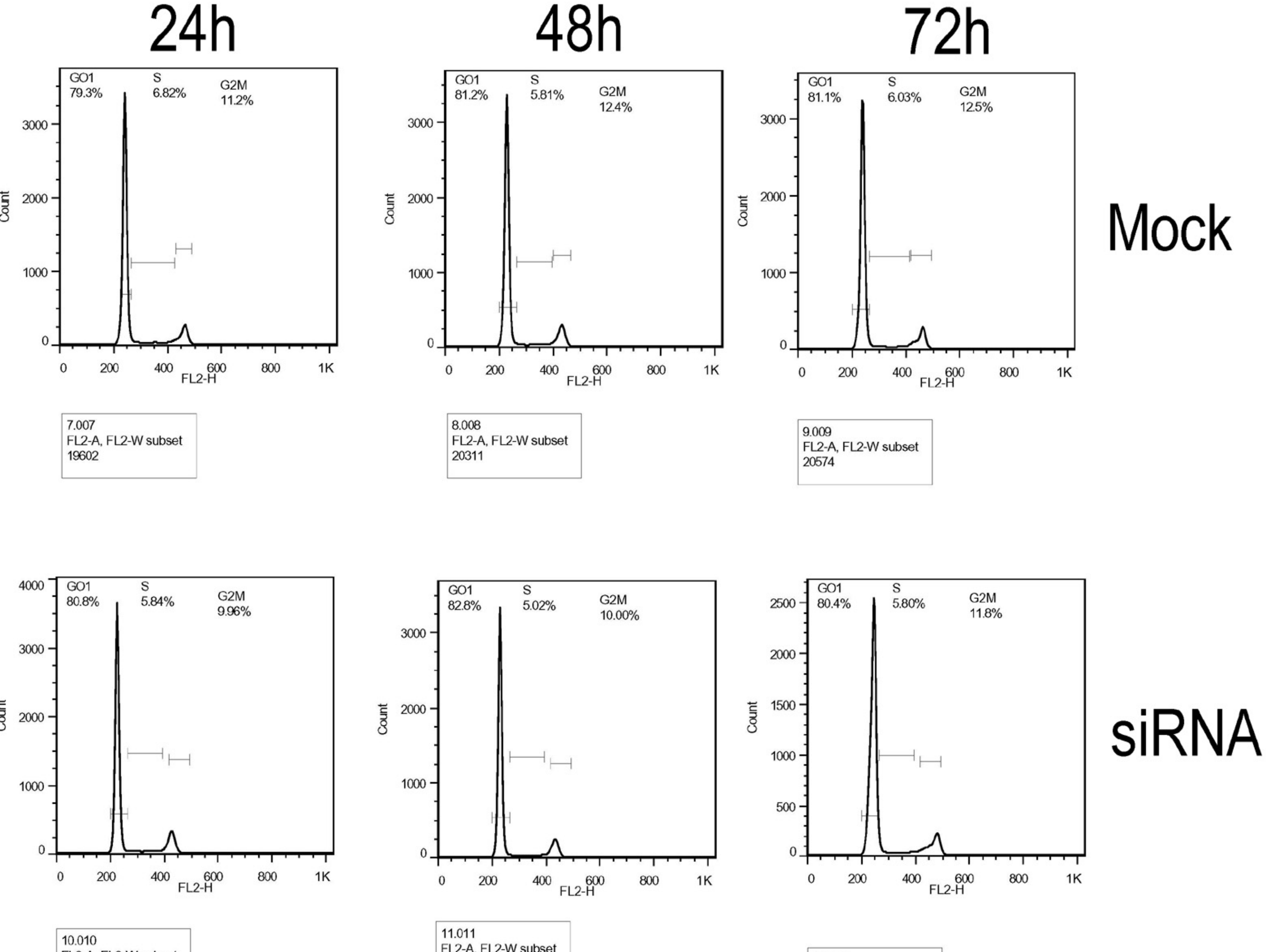

24h
48h
72h
Mock
siRNA
GO1
79.3%
S
6.82%
G2M
11.2%
7.007
FL2-A, FL2-W subset
19602
GO1
81.2%
S
5.81%
G2M
12.4%
8.008
FL2-A, FL2-W subset
20311
GO1
81.1%
S
6.03%
G2M
12.5%
9.009
FL2-A, FL2-W subset
20574
GO1
80.8%
S
5.84%
G2M
9.96%
10.010
FL2-A, FL2-W subset
21019
GO1
82.8%
S
5.02%
G2M
10.00%
11.011
FL2-A, FL2-W subset
17626
GO1
80.4%
S
5.80%
G2M
11.8%
12.012
FL2-A, FL2-W subset
20212
Count
FL2-H

Figure S4

Figure S5

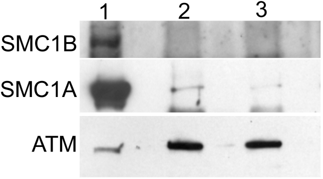

Figure S6

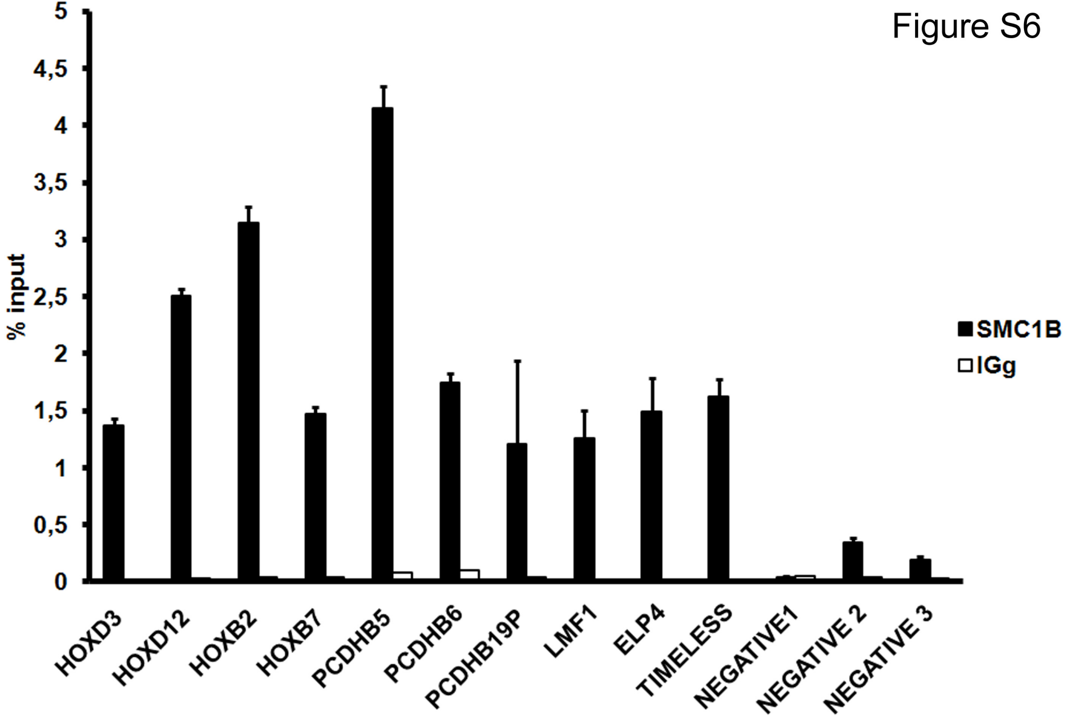

5
4,5
4
3,5
3
2,5
2
1,5
1
0,5
0
% input
SMC1B
IGg
HOXD3
HOXD12
HOXB2
HOXB7
PCDHB5
PCDHB6
PCDHB19P
LMF1
ELP4
TIMELESS
NEGATIVE1
NEGATIVE 2
NEGATIVE 3

Figure S7

A

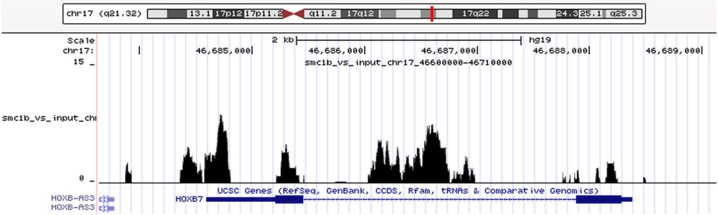

chr17 (q21.32)
Scale
chr17:
2 kb
hg19
46,685,000
46,686,000
46,687,000
46,688,000
46,689,000
smc1b_vs_input_chr17_46600000-46710000
UCSC Genes (RefSeq, GenBank, CCDS, Rfam, tRNAs & Comparative Genomics)
HOXB-AS3
HOXB7

B

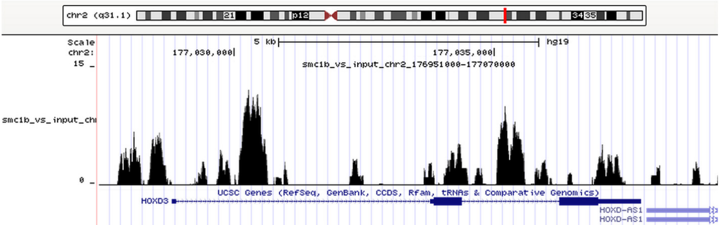

chr2 (q31.1)
Scale
chr2:
5 kb
hg19
177,030,000
177,035,000
smc1b_vs_input_chr2_176951000-177070000
UCSC Genes (RefSeq, GenBank, CCDS, Rfam, tRNAs & Comparative Genomics)
HOXD3
HOXD-AS1
